# Co-Develop-IT! Unifying Methodological Guideline for the Co-Design, Development, and Evaluation of Individually Tailored Technology-Enhanced Training and Rehabilitation Concepts: Consensus Development Study and Tutorial

**DOI:** 10.2196/84163

**Published:** 2026-05-22

**Authors:** Patrick Manser, Lotte ES Hardeman, Andreas Wallin, David Moulaee Conradsson, Breiffni Leavy, Franziska Albrecht, Melvyn Roerdink, Jean-Jacques Temprado, Eling D de Bruin, Erika Franzén

**Affiliations:** 1 Division of Physiotherapy, Department of Neurobiology, Care Sciences, and Society, Karolinska Institutet, Alfred Nobels Allé 23, Huddinge, 14183, Sweden, 46 709237445; 2 Department of Human Movement Sciences, Faculty of Behavioural and Movement Sciences, Vrije Universiteit Amsterdam, Amsterdam, The Netherlands; 3 Medical Unit Allied Health Professionals, Women's Health and Allied Health Professionals Theme, Karolinska University Hospital, Stockholm, Sweden; 4 Research and Development Unit, Stockholm Sjukhem Foundation, Stockholm, Sweden; 5 Department of Nutrition and Movement Sciences, NUTRIM Institute of Nutrition and Translational Research in Metabolism, Faculty of Health, Medicine and Life Sciences, Maastricht University, Maastricht, The Netherlands; 6 Department of Nutrition and Movement Sciences, MHeNs Institute of Mental Health and Neurosciences, Faculty of Health, Medicine and Life Sciences, Maastricht University, Maastricht, The Netherlands; 7 Institut des Sciences du Mouvement (ISM), UMR 7287, CNRS, Aix Marseille Université, Marseille, France; 8 Motor Control and Learning Group, Institute for Biomechanics, Department of Health Sciences and Technology, ETH Zurich, Zurich, Switzerland; 9 Department of Health, OST, Eastern Switzerland University of Applied Sciences, St Gallen, Switzerland

**Keywords:** community participation, digital health, disease prevention, exergaming, health promotion, participatory research, patient participation, rehabilitation, user-centered design

## Abstract

**Background:**

Applying digital health technologies (DHTs) for health promotion and disease prevention is recommended by official bodies such as the World Health Organization. User-centered co-design with systematic patient and public involvement is considered best practice for developing such complex interventions. Although well-established methodological guides and frameworks are available, an important gap is that they are either holistic but generic, offering minimal operational guidance, or context-specific and operational, but focusing only on subphases of establishing DHT-enhanced interventions.

**Objective:**

This paper presents a unifying consensus-based methodological guideline directed toward multidisciplinary expert teams coordinating projects on individually tailored DHTs. It delineates best practices with operational guidance for each step along the full lifecycle of DHT-enhanced training and rehabilitation concepts—from contextualization, through codevelopment, and evaluation to implementation.

**Methods:**

The Co-Develop-IT guideline was cocreated through a structured expert consensus process that integrated, refined, and expanded on well-established existing guides and frameworks to delineate holistic and context-specific, yet flexible enough, best practices. The process consisted of biweekly 90-minute hybrid meetings between August 2024 and February 2025, in combination with written elaboration, feedback, and revisions between meetings to gradually develop a consensus on best practice recommendations.

**Results:**

The Co-Develop-IT guideline consists of 8 iterative phases. It is applicable to any type of end users, exercise types, intended contexts of use (eg, primary health care, community health services, and telemedicine), and overarching goals (eg, health promotion and primary through tertiary disease prevention, including rehabilitation). The Co-Develop-IT guideline introduces 5 distinct preparatory contextual research phases preceding generative codevelopment. These phases are dedicated to the structured establishment of a more robust foundation to better tailor and steer codevelopment efforts toward successful implementation. In 2 application examples, we provide proof of concept that the resulting guideline fulfills its primary purpose of providing comprehensive, context-specific, and operational, yet flexible enough best practice recommendations.

**Conclusions:**

The unifying Co-Develop-IT guideline provides comprehensive best practices with actionable operational guidance for establishing an appropriate balance between scientific theories and frameworks and the real-world needs of interest-holders in the establishment of individually tailored DHT-enhanced training and rehabilitation concepts. Applying Co-Develop-IT contributes to overcoming the lingering evidence-to-practice gap by consistently establishing a shared mission with relevant interest-holders and ensuring that all codevelopment steps are directed toward addressing an unmet need in (clinical) practice—ultimately promoting the practical application and impact of purpose-developed DHTs.

## Introduction

Innovations in digital health technologies (DHTs)—such as exergames or electronic and mobile health apps—have attracted significant interest as powerful tools to advance health promotion and disease prevention (primary through tertiary prevention; including rehabilitation) [1-4]. On a system’s level, DHTs stand out as scalable and cost-effective tools that can contribute to health system improvements via hybrid intervention delivery in a well-controlled environment [2,4-6]. On the individual level, such DHTs have been mainly used to promote engagement and adherence [2,3]. Recent research, however, shows that their benefits go beyond fun and engagement, as they can be designed to provide superior adherence to relevant principles of behavior change, neuroscience, and exercise science compared to conventional exercise and rehabilitation modalities [2].

User-centered methodologies, which continuously involve end users and other multidisciplinary interest-holders in a participatory process, are considered best practice for designing and evaluating complex interventions such as DHT-enhanced interventions. Several guides and frameworks have been published in recent years to methodologically guide these procedures. However, an important gap in the existing guides and frameworks is that they are either holistic but generic, offering minimal operational guidance, or context-specific and operational, but focusing only on subphases of establishing DHT-enhanced interventions.

The UK Medical Research Council’s (MRC) guidance for complex interventions [7] is the most frequently used methodological guide for rehabilitation interventions for older adults [8]. It holistically covers intervention development, evaluation, and implementation with core elements to be considered at each phase. These core elements provide valuable guidance for navigating the challenges of the development and evaluation of complex interventions but may lead to difficulties in applying recommendations due to minimal operational guidance [9]. Furthermore, the MRC guidance applies to all complex interventions (ie, all interventions with several interacting components, from public health interventions, over new surgical techniques, to neurorehabilitation) [7]. Consequently, this broad guidance has often been complemented with more specific frameworks to better fit project-specific contexts [8].

In the broad field of electronic health system development, user-centered design [10] has been the most frequently applied framework [11]. More specific guidance for health care contexts is provided by the generative co-design framework for health care innovation [12]. For DHTs specifically, the nonadoption, abandonment, scale-up, spread, and sustainability (NASSS) framework [13], the Multidisciplinary Iterative Design of Exergames (MIDE) framework [14], or its refined methodology from the “Brain-IT” project [15] provide guidance on how to structure the conceptualization, co-design, and evaluation process. These key frameworks represent important steps to advance research methodologies. However, they only cover 1 or multiple subphases relevant to the establishment of DHT-enhanced interventions, leading to a fragmented landscape of frameworks with varying recommendations. Therefore, the research community would benefit from a unifying methodological guideline that delineates more context-specific, yet flexible enough, best practices [8].

Our aim was to cocreate and present a unifying consensus-based methodological guideline directed toward multidisciplinary expert teams coordinating projects on individually tailored DHTs. It delineates best-practice recommendations and operational guidance for each step along the full lifecycle of individually tailored DHT-enhanced training concepts—from contextualization through codevelopment and evaluation to implementation.

## Methods

### Overview of the Consensus Process

The Co-Develop-IT guideline was cocreated through an expert consensus process. It consisted of biweekly 90-minute hybrid (in-person+videoconference) meetings beginning in August 2024. As per the nominal group technique [16], each consensus group member was asked to critically reflect on and note down their ideas before each meeting for the topic to be discussed. These ideas were shared at the start of each meeting in a brainstorming phase, followed by clarifications of the proposed ideas where necessary [17]. Similar to round 4 of a Delphi approach [18], the consensus group discussed the relevance of proposed items, but with a focus on giving deviating ideas room for justification and discussion to ensure no elements are overlooked. Finally, the consensus group consolidated proposed ideas by cocreating checklist items and discussing the order and overall structure of the best-practice recommendations.

After each meeting, the first author prepared meeting minutes, synthesized the outcomes, and drafted the corresponding sections of the guideline, checklist, and explanation and elaboration sections. All authors provided written elaboration, feedback, and revisions between meetings. Drafts were version-controlled, and between-meeting changes were ratified at the subsequent session to finalize content.

For each step (steps 1 to 3; “Co-Ccreation of Co-Develop-IT” section), this process continued until (1) saturation (no new items emerging across 2 consecutive meetings) and (2) consensus on a finalized Co-Develop-IT guideline package, which was achieved in February 2025 and is presented in the Results section. Consensus was defined as unanimous agreement among the consensus group or, where this could not be achieved and conflicting recommendations remained, a supermajority decision (threshold of ≥80%) during a cocreation meeting.

### Disciplinary and Professional Background of the Consensus Group

We recognize that the Co-Develop-IT guideline is shaped by the authors’ disciplinary and professional backgrounds. To provide a nuanced and integrative guideline, the consensus group was carefully selected to represent experts with hands-on experience in applying the methodological guides and frameworks we sought to combine, refine, and expand. The group includes researchers focused on the contextualization and codevelopment of conventional (8 of 10 authors [PM, AW, DMC, BL, MR, JJT, EDB, and EF]) and DHT-enhanced (7 authors [PM, LEHS, DMC, BL, MR, JJT, and EDB]) training and rehabilitation interventions, efficacy or effectiveness (all authors), and mechanistic (6 authors [PM, AW, FA, JJT, EDB, and EF]) evaluations, and implementation in clinical practice (7 authors [PM, LEHS, AW, DMC, BL, MR, and EDB]). In total, 5 authors (PM, DMC, MR, JJT, and EDB) bring experience with public-private partnerships as well as commercialization and related regulatory efforts (eg, medical device and other certifications) relevant to sustainable projects on DHTs. We also bring dual appointments bridging academic research with (1) clinical vocational practice (3 authors [LEHS, BL, and EF]) and (2) leadership roles in technology development companies (2 authors [MR and JJT]). The educational backgrounds span health sciences and technology, physiotherapy, movement sciences, neuroimaging, and neuropsychology.

### Cocreation of Co-Develop-IT

#### Step 1: Identification of Guides and Frameworks to Build On

To ensure that we provide a unifying methodological guideline that appropriately builds on and expands previous research, we started with a critical discussion on which guides and frameworks were useful to guide previous projects [19,20]. Consensus was reached to build on the MIDE framework [14] and its refined methodology from the “Brain-IT” project [15] and unify and expand it with the MRC guidance for complex interventions [7], the generative co-design framework for health care innovation [12], as well as the NASSS [13] and the Practical Planning for Implementation and Scale-Up (PRACTIS) [21] frameworks.

#### Step 2: Identification of Focus Areas for a Unifying Methodological Guideline

We critically exchanged methodological learnings from our projects. For example, despite Brain-IT’s success in maximizing efficacy and user experience [19,22,23], preparatory focus groups for follow-up projects identified gaps in telemedicine workflow implementability that were overlooked during multidisciplinary codevelopment since only design requirements but not implementation requirements were systematically considered. Combined with the identification of limitations of existing guides and frameworks, we defined the following focus areas for the cocreation of our unifying methodological guideline:

- provide clearer recommendations to harmonize the interests and roles of different contributors—particularly across research, industry, health care, and end users;
- clarify the level of involvement of different interest-holders;
- guide additional methodological steps for the validation of DHT components and to facilitate the implementation, scalability, and sustainability of the solutions to be developed; and
- harmonize the interpretation and implementation of the recommended methodological steps and establish a common terminology between different contributors, who often refer to similar issues with different wordings and theoretical references.

#### Step 3: Cocreation of Unifying Best Practices With Operational Guidance

Cocreation of the Co-Develop-IT guideline package was achieved by focusing our discussions and the consensus-finding process on how well-established guides and frameworks can be integrated, revised, and, most importantly, be expanded to address the identified focus areas for improvement. The process followed the chronological nature of the relevant phases for the establishment of DHT-enhanced training or rehabilitation interventions, but went through several iterative loops to refine best practice recommendations in each phase until consensus was established on a finalized Co-Develop-IT guideline package as per the general consensus handling process defined in the Overview of the Consensus Process section.

### Proof of Concept for Co-Develop-IT

To verify whether the resulting guideline fulfills its primary purpose of providing comprehensive, context-specific, and operational, yet flexible enough best practice recommendations, it was applied to guide the methodological development of 2 ongoing projects*—*“Park-MOVE” (Karolinska Institutet, Sweden) and “Better Together” (Vrije Universiteit Amsterdam, The Netherlands). The application examples are provided to complement the Co-Develop-IT guideline package for additional operational guidance with project-specific examples.

### Ethical Considerations

Given the cocreation of this unifying methodological guideline was done within the author team and we completed no experiments involving human subjects as participants, no ethics approval was necessary. Ethics approval for studies as part of the “Park-MOVE” and “Better Together” application examples was provided by the Swedish Ethical Review Authority (2024-06688-01) and the Research Ethics Committees United, The Netherlands (R22.076, NL82441.100.22), respectively.

## Results

### Overview

The *Co-Develop-IT guideline package* consists of the following complementary elements:

1. Co-Develop-IT core guideline (see “The Co-Develop-IT Guideline” section),
2. explanation and elaboration sections of the Co-Develop-IT core guideline (see “Explanation and Elaboration of the Co-Develop-IT Guideline” ),
3. Co-Develop-IT checklist (Supplementary File 1 in Multimedia Appendix 1), and
4. explanations and elaborations of each checklist item with guiding examples (Supplementary File 2 in Multimedia Appendix 1).

#### For Which Contexts Are the Co-Develop-IT Guideline Intended?

The Co-Develop-IT guideline was developed for the context of individually tailored DHT-enhanced training and rehabilitation concepts that are purposefully codeveloped with adequate theoretical underpinnings.

With “DHT-enhanced training or rehabilitation concepts,” we refer to:

- a conceptual guideline that delineates all algorithmic decision trees regarding the structure, content, tailoring, delivery, supervision, and monitoring of a DHT-enhanced exercise, training, or rehabilitation program (in adherence with the definitions from Manser et al [2] and Herold et al [24]), combined with:
- a purpose-developed DHT to implement and deliver the exercises, training, or rehabilitation program according to this concept.

For the sake of terminological simplicity, we will refer to “DHT-enhanced training concepts” from here on out.

With “individually tailored” training, we refer to integrating both “personalization” and “individualized progression,” understood as follows in the context of this guideline:

- Personalization refers to the systematic selection of the type, content, components, and characteristics of a training or rehabilitation program for a specific person based on their (clinical) characteristics, performance, or preferences at baseline.
- Individualized progression refers to the gradual and systematic adaptation of training dose and stimuli to an individual in response to their training or rehabilitation participation to maintain a training load deemed sufficient to induce targeted health- or disease-related adaptations and/or to promote user experience, engagement, and adherence.

#### Who Is the Co-Develop-IT Guideline Intended For?

The Co-Develop-IT guideline is directed toward multidisciplinary expert teams coordinating projects that involve the contextualization, codevelopment, evaluation, and implementation of individually tailored DHTs and DHT-enhanced training concepts. It is applicable for any types of end users, exercise types, intended contexts of use (eg, primary health care, community health services, and digital health), and overarching goals (eg, health promotion and disease prevention [primary through tertiary prevention; including rehabilitation]).

#### How Is the Co-Develop-IT Guideline Intended to Be Used?

To facilitate and support the effective implementation of the conceptually discussed Co-Develop-IT core guideline, we provide the Co-Develop-IT checklist (Supplementary File 1 in Multimedia Appendix 1) that details the operational steps to be performed and documented in projects following this guideline. We recommend that the checklist along with its item-specific explanation and elaboration sections (Supplementary File 2 in Multimedia Appendix 1) be considered the core instruments to work with when implementing the guideline.

#### What Are Key Elements of the Co-Develop-IT Checklist?

Apart from step-by-step instructions for the procedures to be completed and documented when adhering to the Co-Develop-IT checklist, it defines the following “contextual requirements” for each item: (1) the minimum level of interest-holder involvement according to the participation choice points defined by Vaughn and Jacquez [25], (2) which key interest-holders should be responsible for each item, and (3) item relevance when adhering and reporting a project according to the Co-Develop-IT guideline.

#### How Does the Co-Develop-IT Guideline Balance Rigor With Practical Flexibility?

The guideline’s cocreation was centered around finding a good balance between methodological rigor and practical feasibility, which was confirmed successful in 2 application examples. Accordingly, “recommended” and “beneficial” items are provided in the Co-Develop-IT checklist. While every project would benefit from adhering to all items, we acknowledge that, depending on the resources available, projects might not be able to do so. Therefore, we offer flexibility by the opportunity to choose a nuanced approach between the “beneficial” best practice approach and a short-track option that only adheres to the “recommended” items.

### The Co-Develop-IT Guideline

**Figure 1.**
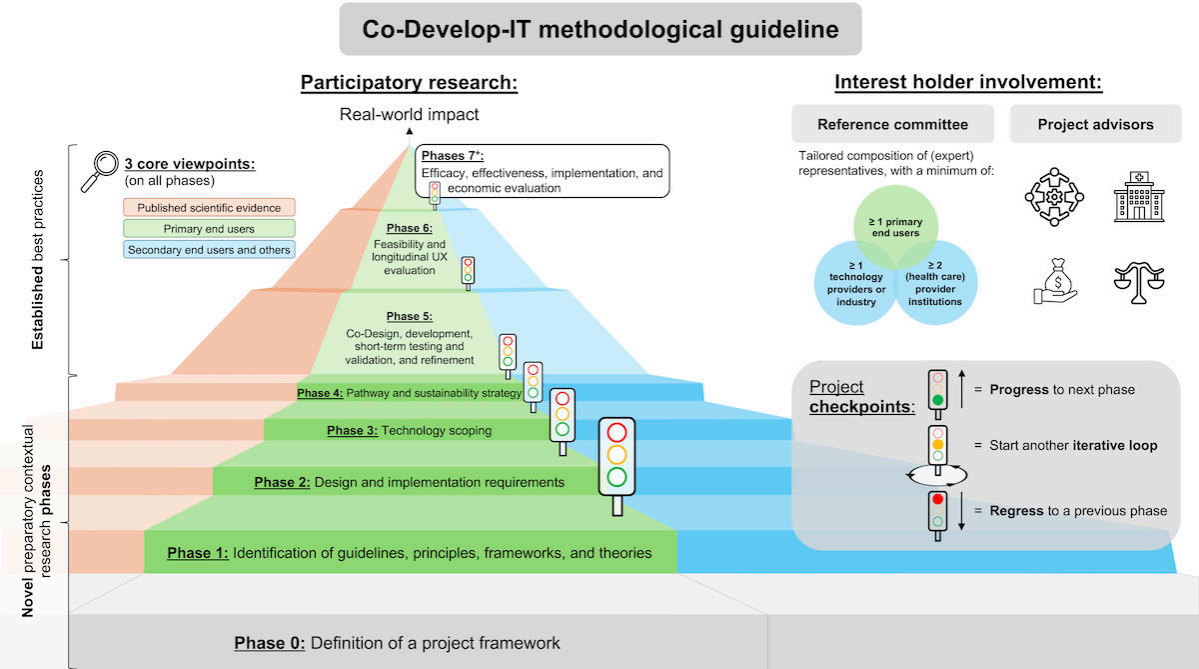
The Co-Develop-IT guideline. This figure illustrates an overview of the key components and phases of the Co-Develop-IT guideline, which systematically guides multidisciplinary expert teams coordinating projects on individually tailored technology-enhanced training and rehabilitation concepts and related digital health technologies toward making a real-world impact. It is applicable to any type of end users, exercise types, intended contexts of use (eg, primary health care, community health services, and telemedicine), and overarching goals (eg, health promotion and primary through tertiary disease prevention; including rehabilitation). UX: user experience.

The Co-Develop-IT guideline is structured in 8 phases. An overview of its key structures and iterative steps that systematically guide projects toward making a real-world impact is illustrated in Figure 1.

Importantly, while both the guideline and each of the phases that build on each other may be perceived as presented rather linearly, they represent iterative project steps, meaning that a project moves up and down the phases—which may each involve multiple iterative loops—depending on goal achievement and project progress. A core component of the guideline is the establishment of project checkpoints with a traffic light system–based assessment framework with clear benchmarks and progression criteria to adequately guide project progression, as well as the number of required iterations within and across phases. Thereby, a systematic and transparent approach to assess goal achievement is established, leading to iteratively looping a phase (=amber light) until the criteria to progress to the next phase (=green light) or regress to a previous phase to establish a more robust foundation (=red light) are met. Of course, this is also applicable to subprojects.

We acknowledge that details of specific steps and their order within a phase might not be applicable to every subproject and might run to a certain extent in parallel. If necessary, details of specific steps or their order may be adapted. However, the order of completing each phase must be followed, as each phase builds on the findings of the previous phases.

Of note, all findings derived from projects adhering to the Co-Develop-IT guideline should be reported following the appropriate checklists provided on the EQUATOR (Enhancing the Quality and Transparency of Health Research) network [26] to ensure reproducibility, transparency, and comparability to other research.

### Explanation and Elaboration of the Co-Develop-IT Guideline

#### Phase 0: Definition of Project Framework

##### Overview

The initial phase of the process of applying the Co-Develop-IT guideline is dedicated (1) to defining nonnegotiable key elements of the project (ie, phase 0.1); (2) to defining requirements on interest-holder involvement, resources, and the regulatory environment for the project (ie, phase 0.2); (3) to establishing a project consortium including all relevant interest-holders and defining their roles and responsibilities throughout the project for effective collaboration and accountability (ie, phase 0.3); and (4) to collaboratively agreeing on specific project checkpoints and a preliminary project time plan (ie, phase 0.4).

These steps are to establish a collaboratively agreed-upon framework within which the project should take place.

##### Phase 0.1: Define Overall Context and Goals of the Project

Initiate the project by outlining its:

- overarching goals and broader intended context (Co-Develop-IT checklist 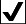-item 3; eg, which structures or functions the training should impact and the type and multidisciplinary expertise of involved institutions),
- target populations (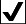-item 4; eg, individuals at risk for or living with a specific disease or condition as primary end users, along with health care professionals and relatives or supporters as secondary end users),
- intervention type (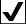-item 5; eg, DHT-enhanced multidomain [physical, motor, and cognitive] training), and
- targeted outcome domain (-item 6; eg, health-related quality of life).

Outlining these aspects allows delineating a clear path to follow, sets clear boundaries for the freedom of the subsequent codevelopment process toward fulfilling a project’s larger purpose, and guides the identification of relevant interest-holders to be recruited (phase 0.2). Therefore, make sure these definitions are backed on a clearly defined theoretical rationale for how the development of an individually tailored DHT-enhanced training concept can address a specific problem or knowledge gap (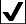-items 1 and 2). Leave sufficient room for innovations and novel ideas that may arise during the codevelopment process. To do so, no specifics such as exercise or training variables, specific DHTs to be used to implement the training, or any design elements of the DHTs and the training are to be defined at this stage. Once a project consortium is established (phase 0.4), these initial definitions are to be refined and agreed on collaboratively with all relevant interest-holders ( 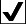-item 14)—thereby providing a structured approach to ensure that the overall project context and goals are backed by both a theoretical rationale and real-world end user and public needs.

##### Phase 0.2: Define Requirements on Interest-Holder Involvement, Resources, and Regulatory Environment for the Project

In the second subphase, identify:

- contextual requirements and required multidisciplinary interest-holders (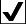-item 7),
- required resources for each interest-holder (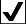-item 8; eg, time, staff, funding, expertise, facilities, and data resources), and
- regulatory requirements (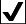-item 9) to successfully complete the project (eg, institutional, ethics, and good clinical practice regulations and requirements for collaboration agreements).

Table 1 provides a list of potential interest-holders to be considered to ensure that the project is grounded in a realistic and competent environment.

**Table 1.** Definitions and key role of multidisciplinary interest-holders to be considered to be involved in research projects in the co-design, development, and evaluation of individually tailored digital health technology (DHT)–enhanced training concepts.

Interest-holder	Definition and role
Research institutions	Academic or research institutions that provide the scientific and methodological expertise necessary for the project.
Health care or other provider institutions	Health care facilities or other provider organizations that facilitate access to the target population, provide clinical insights and support, and implement the resulting DHT-enhanced training concept.
Technology developers	Companies or teams responsible for the development of DHTs (ie, software, hardware, or both).
Technology providers	Companies or teams responsible for supplying the technological infrastructure (software, hardware, computing power, etc) and providing support for the implementation of the DHT-enhanced training concept (eg, installation of devices and ongoing technological maintenance and support for DHTs).
Legal offices	Legal experts who advise the core project team to ensure that all aspects of the project comply with relevant regulations and ethical standards and support and guide efforts toward getting the final DHT-enhanced training concept patented, certified as a medical device, and commercialized.
Additional interest-holders	Depending on the project’s scope, additional interest-holders might include patient advocacy groups, funding bodies, policymakers, and end-user representatives.

Moreover, include legal experts at this preparatory stage to outline the steps required to patent the final training concept or obtain medical device certification for the DHTs (ie, software and hardware). This will ensure that all relevant aspects are considered during the project. This may include adhering to standards set by regulatory bodies such as the Food and Drug Administration in the United States or the European Medicines Agency in Europe. The recently published Common European Classification Grid for digital medical devices is helpful to facilitate the definition of the taxonomy and evidence requirements for assessment of DHTs and offers the possibility to have a common reference on a European level against which national classifications and evidence requirements can be mapped [27]. Key considerations include device classification, safety and efficacy testing, cost-effectiveness evaluation, data security, labeling, and postmarket surveillance.

##### Phase 0.3: Establish a Project Consortium and Define Each Interest-Holder’s Roles Throughout the Project

In the third subphase, recruit interest-holders to establish a project consortium based on the drafted project goals and context from subphase 0.1 and the requirements for a complete project consortium defined in phase 0.2. Ensure that at least 1 interest-holder representing each target population (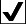-item 4) and multidisciplinary context (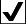-item 7) is involved.

Establishing a project consortium entails setting up a project reference committee ( 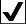-item 10b; recommended item) and including project advisors (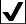-item 10c; beneficial item) to continuously guide the core project team (-item 10a; recommended item). In the case of multinational projects, establish a project reference team and recruit project advisors for each country involved to ensure that local and sociocultural nuances are appropriately considered. Tailor the project consortium’s constellation to the specific research project, adhering to the minimum key interest-holders and responsibilities listed in Table 2. Of note, the involvement of members of the project consortia might change throughout the conduct of a project, depending on the project-specific requirements, needs, or relevance in different phases.

**Table 2.** Recommendations on key interest-holders and responsibilities of core groups to be involved in research projects following the Co-Develop-IT guideline.

Core groups	Recommended members	Responsibilities	Level of involvement^a^
Core project team	The core project team typically consists of the team who initiated the project. As a minimum requirement, this project team includes: the project coordinators or the local principal investigators of the project and their research teams ( 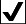 -item 7a), interest-holders from the health care or other provider institutions ( 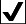 -item 7b), and if the technology development is not done within the research teams the technology developers ( 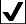 -item 7c).	To coordinate and monitor the successful conduct of the project.	Minimum condition: “involving” level Ideal condition: “collaborating” or “empowering” level
Project reference committees	While the constellation of each of the project reference committees should be tailored to the specific research project, we recommend that the project reference committees should include: ≥1 (expert) representative of the primary end users (eg, patient advocacy groups or end-user representatives; 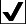 -item 7f), ≥1 (expert) representative of the technology providers or industry ( 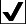 -item 7c), and ≥2 (expert) representatives of the health care or other provider institutions ( 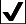 -item 7b): 1 on the organizational level ( 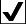 -item 4e) and 1 on the individual level ( 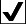 -item 4c).	At the beginning of the project: To collaboratively work out and define specific subgoals and progression criteria ( 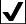 -item 15) with the project core team. To advise the project core team when working out a preliminary time plan ( 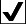 -item 16). In later stages of the project: To guide important decisions on the project plan and execution. To predetermine, together with the core project team, the weighting of how input from different interest-holders should be prioritized when making design decisions in phase 5 ( 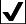 -item 28). To determine, together with the core project team, how to prioritize and weight the contribution between the 3 core viewpoints when making specific design decisions in the case of potential incongruent findings or conflicting results (eg, phase 2 [ 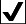 -items 19‐23] or phase 5 [ 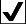 -item 34]).	Minimum condition: “involving” level Ideal condition: “collaborating” or “empowering” level
Project advisors	The project advisors may include representatives for each of all remaining defined levels under 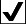 -items 4 and 10 and may primarily consist of: communities and systems in which the training concept should be implemented ( 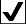 -item 4e), legal offices ( 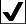 -item 7c), and additional interest-holders, such as funding bodies or policymakers ( 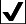 -item 7d) depending on the project-specific requirements and as defined under 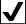 -item 7.	To provide nonbinding strategic advice to the core project team on: the project plan and execution, including regulatory and ethical compliance, how to deal with potential incongruent findings or conflicting results between the 3 core viewpoints, to take and refine measures to comply with recommendations on diversity and inclusion, anticipation and reflection, openness and transparency, responsiveness, and adaptive change, and to support commercialization, patenting, or medical device certification of the DHT^b^-enhanced training concept.	Minimum condition: “consulting” role on the individual level

a Level of participatory research involvement according to the participation choice points outlined by Vaughn and Jacquez [25].

b DHT: digital health technology.

To ensure project feasibility and mitigate potential risks of noncompletion, comprehensive resource mapping and comparing the available resources for all interest-holders (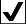-item 11) with the required resources (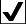-item 8) helps to identify any shortcomings to be addressed to support smooth project execution and completion (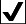-item 12). Critically reflect on any relevant additional project-specific resources and risks to ensure thorough risk mitigation.

Establish formal collaboration agreements that delineate the responsibilities, rights, obligations, and degree of involvement of each collaborator (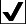-item 13) to facilitate the management of expectations and ensure accountability. Regularly revisit and update the collaboration agreements as needed to reflect any changes in the project scope or interest-holder roles.

##### Phase 0.4: Collaboratively Agree on Specific Subgoals and Progression Criteria as well as a Preliminary Time Plan for the Project

Finally, collaboratively refine and agree on the specific goals and subgoals of the project (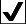-items 14 and 15a; as drafted in phase 0.1) and define project checkpoints (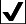-item 15) that are aligned with the overall goals of the project and its phases—both based on a consensus process with all interest-holders of the project consortium (ie, core project team and reference committee and [optionally] project advisors). Each project checkpoint requires a traffic light system–based assessment framework with clear benchmarks (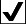-item 15c) and progression criteria (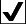
- item 15d) to ensure transparency in the assessment of agreed-upon goals (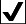-item 15b). The project checkpoints are usually to be positioned toward the end of each phase to inform whether the project can progress to the next phase (=green light), requires further iterative loops with refinements before proceeding to the next phase (=amber light), or requires the establishment of a more robust foundation by regressing to a previous phase (=red light).

Descriptive examples are provided in the “Phase 5.6: Iterative Refinements” section, Supplementary File 2 in Multimedia Appendix 1, and in previous literature of the author team in relation to feasibility and user experience testing in preparation for a randomized controlled trial [23,28]. For transparency in the interpretation of project results and progression, we recommended that the traffic light system–based assessment framework be published (as part of a project protocol) or at a minimum publicly preregistered (eg, on the Open Science Framework) prior to the start of data collection.

Collaboratively agreeing on these goals and checkpoints establishes a unified direction and commitment to the project’s success and helps prevent the emergence of misunderstandings or conflicts at a later stage of the project. To support these efforts, adopt techniques such as empathetic target group analysis [29] to foster a better understanding of the diverse needs and perspectives among interest-holders before starting the co-design or cocreation process [30,31]. Moreover, have the added value and expectations of each partner in collaboratively achieving these goals communicated to transparently foster fruitful collaboration.

Finally, a major pitfall in DHT-related projects with public-private partnerships is the potential misalignment in expected rates of progress and output per unit of time in public-private partnerships, given that public research institutions may be perceived by the industry as slow-moving “oil tankers”, whereas the industry may be seen by academia as agile “speedboats” rushing toward marketable outputs. Therefore, we recommend collaboratively developing and agreeing on a preliminary project time plan (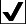-item 16) to align interest-holder expectations and update this time plan regularly to harmonize expectations between partners.

### Phase 1: Identification of Guidelines, Principles, Frameworks, and Theories

Phase 1 is dedicated to identifying guidelines and evidence-based recommendations for the overall goals of the project (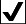-item 17) along with principles, frameworks, or theories (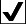-item 18). This phase is key to defining a robust backbone and guiding future steps of projects, particularly decisions on the design, characteristics, and content of the DHT-enhanced training concept to be developed. A few broadly applicable examples that the consensus group recommends following are included in Textbox 1.

Textbox 1. **Examples of broadly applicable guidelines, principles, frameworks, and theories the consensus group recommends to follow.**

**Guidelines and evidence-based recommendations**

- Clinical or best practice guidelines for a specific target population
- Consensus statements, such as the global consensus for optimal exercise recommendations for enhancing healthy longevity in older adults [32]

**Principles**

- General training principles [24,33]
- Neuroplasticity principles [34,35]
- Principles for neurorehabilitation (ie, that integrate motor learning and brain plasticity mechanisms) [36]

**Frameworks**

- A behavior change framework applicable to physical activity or training [37]
- Guided plasticity facilitation framework [38] for motor-cognitive training
- The adaptive capacity model [39]
- The Beyond “Just” Fun of Exergames framework [2]

**Theories**

- Theory of effort minimization in physical activity [40,41]
- Optimizing performance through intrinsic motivation and attention for learning theory of motor learning [42]
- A behavior change theory supporting initiation and consolidation of physical activity behavior changes [43]

### Phase 2: Determine Design and Implementation Requirements

The overarching aim of phase 2 is to delineate a comprehensive set of design and implementation requirements for the DHT-enhanced training concept. This encompasses a range of considerations to be adhered to throughout the subsequent codevelopment phases, including:

- generating user models (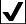-item 19),
- defining requirements for core components of individually tailored training concepts (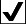-item 20),
- defining environmental requirements (eg, training equipment, space requirements, and connectivity such as Wi-Fi or Bluetooth; 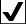-item 21), and
- defining hardware (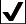-item 22) and software (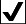-item 23) requirements for the DHTs.

Elaboration of the design and implementation requirements should be built on phase 1 by integrating identified guidelines and evidence-based recommendations (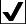-item 17) and taking into account relevant principles, frameworks, and theories (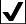 item 18). Consider the set of core questions provided in Supplementary File 5 in Multimedia Appendix 1 along with the checklist facilitating DHT adoption provided by Hamasaki et al [44] and a comprehensive (early) health technology assessment (eg, see exemplary question in Table 2 of Grutters et al [45]) to explicitly evaluate the potential value of a health technology [45] to ensure that all potentially relevant aspects are considered. All decisions are to be made by integrating the findings from three core viewpoints: (1) a synthesis of published scientific evidence, together with the findings from performing qualitative research on the perspectives of (2) the intended primary end users, and (3) the intended secondary end users and other relevant interest-holders.

The current state of the scientific evidence should be derived from conducting an umbrella review, meta-analysis, systematic, scoping, or narrative review based on the established levels of evidence [46]. If such high-level evidence is available in recent scientific literature, a narrative synthesis of the current state of evidence suffices. The perspectives of intended primary and secondary end users and all other relevant interest-holders should be derived from conducting qualitative research (based on, eg, semistructured interviews, focus groups, or a [modified] Delphi approach [18]). Refer to 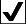-item 29 on how to set the stage to facilitate reflective dialog and productive workshops and apply these recommendations also to the qualitative research components in this phase (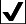-items 19‐23).

At this stage of a project, we recommend conducting qualitative research with primary end users separately from the remaining interest-holders. While all interest-holders are later on recommended to codevelop solutions together, this structured approach builds the foundation for productive codevelopment. Specifically, it ensures that primary end users—who might be hesitant to share their insight in the presence of field experts on DHTs or exercise and rehabilitation approaches—get their own platform to express all their insights fully and in the absence of potential power dynamics that might limit their participation [47]. By aligning data collection on design and implementation requirements to reflect the 3 core viewpoints from which insights are to be combined ensures that no important insights are missed, particularly regarding barriers to and facilitators of sustained adoption, including considerations for training and associated learning curve when using DHTs and establishing a supportive organizational culture [48,49]. Furthermore, it fosters a better understanding of the diverse needs and perspectives among different interest-holder groups and facilitates that all those perspectives are presented to empower all codevelopment participants in phase 5 to contribute fully [30,31].

To provide a more nuanced understanding of how inclusivity and accessibility can be ensured for all relevant intended end users, consider examining the current state of evidence and interview or focus group transcripts through the lens of critical discourse analysis [50]. These insights should be aligned with broader frameworks for sustainable innovation and ethical design in digital health [51].

Based on the project checkpoints, phase 2 evaluations should be repeated iteratively (=amber light; eg, due to insufficient coverage of relevant themes as per feedback from the project reference committee) until the green light criteria for project progression are met, at which point, the project can progress to phase 3. If red light criteria are met (eg, due to a strong mismatch between defined project goals [ 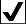-item 14] and “real-world” needs as per findings from the qualitative studies), it is suggested that the project requires a more robust foundation to be established by returning to phase 2, as per the progression criteria defined in phase 0 (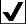-item 15).

### Phase 3: Technology Scoping

The third phase focuses on identifying (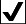-item 24a) and critically appraising (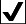-item 24b) existing (partial) DHT solutions in both research and the market that align with the project’s overall goals (phase 0). Apply techniques such as trend analysis and the Strengths, Weaknesses, Opportunities, and Threats matrix [14] to gain a comprehensive understanding of the current state of knowledge and to identify areas for further improvement. Moreover, assess each identified solution against the design and implementation requirements defined in phase 2 (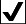-item 24c). Understanding the strengths, limitations, and areas for improvement of current solutions in relation to the design and implementation requirements is essential for building a robust foundation for advancing the field with credible, innovative, and evidence-based DHT-enhanced training concepts. Finally, identify and analyze emerging trends (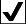-item 24d) to ensure that the project identifies and integrates relevant technological advancements that may be relevant to better fulfill the design and implementation requirements. This is especially important for rapidly evolving technological opportunities, such as augmented or mixed reality, artificial intelligence, bio- or neurofeedback, and brain-computer interfaces [2].

### Phase 4: Define Pathway and Sustainability Strategy

The aim of phase 4 is to make a strategic decision on the project’s development path ( 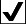-item 25) and establish a sustainability strategy for the DHT-enhanced training concepts to be (further) developed (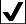-item 26). Specifically, on the basis of the findings of phases 1‐3, determine whether the project strives to develop a novel DHT-enhanced training concept from scratch (path 1) or further develops and builds on existing solutions (path 2).

A purpose-developed software builds the heart of every DHT, whereas the conceptual decisions and algorithmic decision trees provided in a training concept build the heart of a DHT-enhanced training concept [2]. Therefore, to maximize scalability and transferability to other application scenarios or use cases, focus on developing training concepts and software to implement the training while relying on (1) well-established, off-the-shelf hardware and (2) ensuring the software is universally applicable with different hardware peripherals. This adheres to the “training (instead of product) first” approach [52] and facilitates the sustainability strategy, as it reduces the complexity of providing necessary equipment and improves scalability.

In this regard, under 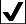-item 26, a sustainability strategy is to be developed to ensure that the solution (DHT-enhanced training concept) to be (further) developed will be made available to the intended target populations and remain available after the project’s completion. This strategy should address its long-term availability, which could be provision and maintenance of open-access to software, developing a business plan and outlining a plan to commercialize the solution, or transferring intellectual property rights to an existing company. Depending on this, mechanisms for ongoing maintenance and support, scalability to reach a broader audience or adapt to different contexts, and identifying resources to support the long-term sustainability of solutions are to be worked out. This step is crucial to ensure that the project’s outcomes have a lasting impact and continue to benefit the target populations in the long term.

With these extensive preparatory contextual research steps from phases 1 to 4, a robust conceptual foundation for the targeted codevelopment toward successful implementation phases of the project is laid.

### Phase 5: Co-Design, Development, Short-Term Testing and Validation, and Refinement

#### Overview

Phase 5 consists of multiple iterative cycles of (1) co-designing and developing prototypes of the DHT-enhanced training concept (phases 5.2 to 5.4), (2) short-term user experience and safety testing and validation of components of the DHT-enhanced training concept (phase 5.5), and (3) refining these prototypes by further co-design and development (phase 5.6).

This iterative cycle is to be repeated until an “acceptable” solution (=all components of the DHT-enhanced training concepts have been successfully validated, have a good user experience, and are safe) is achieved. The result of this phase is an “original” DHT-enhanced training concept that enters the next phases of longitudinal evaluations.

#### Phase 5.1: Build a Framework for the Co-Design

First, identify congruent aspects from previous project phases (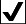-item 27a) and agree on which of the remaining aspects for the DHT-enhanced training concepts require co-design procedures (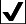-item 27b). Subsequently, work out and define, together with the project reference committees, the weighting of how inputs from different interest-holders are to be prioritized when collaboratively making design decisions (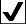-item 28). For example, it might be agreed that design decisions on the graphical user interface should primarily be driven by primary and secondary end users, whereas algorithms for individualized tailoring of the training should mainly rely on scientific theories with input from researchers and health care professionals. Clear definitions of each interest-holder’s role and expected contributions help streamline and guide the complex task of integrating various, potentially conflicting perspectives and viewpoints from different interest-holders—increasing the likelihood of successfully generating conceptual prototypes that fulfill all the design and implementation requirements defined in phase 2 of the project and all involved contributors can agree on.

#### Phase 5.2: Co-Design Workshops

The diversity in background, education levels, and experiences of interest-holders can be expected to lead to power dynamics that influence or limit the interactions during the co-design process [47]. Therefore, a stepwise process is recommended to set the stage for a well-functioning collaborative effort.

Specifically, start the co-design workshops with a short presentation to share the rationale and overall goals of the project (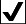-item 29a) and preparatory findings from earlier phases of the project (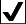-item 29b), and clarify the planned procedures (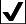-item 29c) and expectations (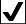-item 29d) with all co-design workshop participants. With respect to the latter, transparently share the weighting and prioritization of input from different interest-holders (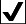-item 28) so that all participants have a clear understanding of their expected contributions while mitigating potential power imbalances. Finally, invest time in establishing mutual trust, empathy, and comfort with all participants (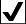-item 29e). This step is critical to foster a better understanding of the diverse needs and perspectives among interest-holders and facilitate reflective dialogue before starting the co-design or cocreation process [30,31]. Use techniques such as empathetic target group analysis [29] to achieve this aim.

If the project chooses path 2—to further develop existing DHT-enhanced training concepts (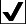-item 27)—present these solutions to all workshop participants to provide them with a clear understanding of what the project builds on and what preparatory steps led to the chosen starting point (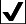-item 30). The participants should also be given the opportunity to try these existing DHTs to help them identify opportunities for improvements that can be addressed in generative co-design workshops.

Finally, the “generative” part of co-design is performed (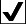-item 31). During the co-design process, use techniques such as 6-3-5 brainwriting [53] to ensure that all interest-holders can express their ideas without judgment [30,31]. To facilitate collaborative idea generation, use different paper- and pencil-based, embodied, or technology-supported prototyping techniques. Simple methods may include, but are not limited to, empathy mapping [29,54], user journey mapping [55], bodystorming [56,57], collaborative sketching and drawings for the rapid development of initial concepts and designs [14], or—when building on and further developing existing solutions—the cognitive walkthrough method [58]. Technology-enhanced prototyping may include the use of middleware interfacing software tools that allow for the rapid development of initial concepts and designs to the creation of prototypes of the proposed concepts (eg, game mechanics and theme) in game engines. Such tools allow early identification of possible modifications that must be performed to facilitate user interaction (eg, removing the need to press buttons) and are particularly useful for the development of gamified DHTs [14].

#### Phases 5.3 and 5.4: Data Synthesis and Development

Analyze data generated during the “generative” part of co-design, such as audio (and video) recordings combined with generated sketches and drawings or virtual conceptual prototypes, using qualitative research methodologies (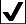-item 32), such as qualitative content analysis [59-61]. Use the frameworks defined in phase 1 to guide the interpretation of findings. For example, the Beyond “Just” Fun of Exergames framework could be useful in this stage to ensure appropriate theoretical backing of DHT designs to relevant principles in motor-cognitive learning, neurorehabilitation, and behavior change [2].

In the next step, present the results from each workshop to the project reference committee to collaboratively integrate the findings and rank-order complementary solutions. It is likely that there are incongruent findings or conflicting results between different workshops and complementary solutions for the same aspects of the DHT-enhanced training concepts from different workshops. In this case, present these results to the project reference committees and collaboratively make consensus-guided decisions on how to integrate the findings into decisions on the design of the DHT-enhanced training concepts. Ideally, consider the advice of project advisors as well.

Based on the derived rank-ordering (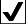-item 33) and considering resource availability (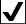-item 11), derive a list of DHT development tasks (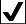-item 34) to establish testable prototypes of the DHT-enhanced training concept (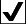-item 35).

#### Phase 5.5: Short-Term Testing and Validation

The first prototypic components of the DHT-enhanced training concepts are then thoroughly tested on safety and user experience (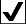-item 36), and relevant components of the DHT-enhanced training concept are validated (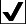-item 37).

User experience refers to a complex characteristic that results from the perception of many distinct quality aspects of a product [62]. The relevance of these quality aspects varies between different contexts and products under evaluation [63-65]. It has been defined as a person’s perceptions and responses resulting from the use and anticipated use of a product, system, or service [66]. For a comprehensive user experience assessment, combine the advantages of both quantitative and qualitative research methodologies. Consider Campbell’s [67] step-by-step instructions and best practices.

For quantitative user experience evaluations, research has often relied on questionnaires for specific subconstructs of user experience, such as usability, which is a prominently evaluated subconstruct in the field of DHTs [65]. For a more comprehensive user experience assessment, we recommend the validated modular framework introduced by Schrepp and Thomaschewski [62]. This framework contains several scales that measure different user experience aspects and allows the construction of customized questionnaires tailored to the specific research question and context of use in a uniform format. While the shortcoming of such a modular questionnaire is the lack of standardized benchmarks, its application is mainly recommended when multiple measurements of the same product are compared over time [62], matching the iterative process proposed by the Co-Develop-IT guideline. This scale may be complemented with other—more specific—scales for certain new use cases and product types to provide a more comprehensive assessment (eg, see item-specific explanation and elaboration statement) [62]. For qualitative user experience evaluations, obtain data complementary to quantitative evaluations to inform further developments and refinements. Specifically, collect specific suggestions for improvements in the components of the DHT-enhanced training concepts to optimize the user experience via semistructured interviews with primary and secondary end users.

Assess the safety of components of the DHT-enhanced training concepts and interaction with DHTs in relation to the characteristics and capabilities of the targeted end users. Consider the perspectives of both primary and secondary end users. We recommend having trained professionals closely observing play-test sessions to assess safety (eg, risk of falls or injuries) along with qualitative research with primary and secondary end users to assess perceived safety and collect suggestions for improvements.

Thoroughly validate the relevant components of the DHT-enhanced training concepts according to the requirements defined under 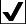-item 15b. This may include but is not limited to software algorithms for player movement detection [68], (game) performance metrics [69-73], (gamified) assessments [74], or mechanisms for individualized tailoring [69].

At the end of this subphase, check whether the quality criteria defined under 15c are reached (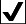-item 38), share the findings with all co-design workshop participants (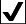-item 39), and progress according to the mechanism outlined under 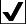-item 15d.

#### Phase 5.6: Iterative Refinements

Iteratively repeat subphases 5.1 to 5.5 until an *“*acceptable” solution is achieved (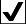-item 40) according to the specific subgoals and quality criteria defined under 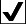-items 15a and 15c. For example, the criteria for an *“*acceptable” (=green light) solution could be (1) session adherence and compliance ≥70%, (2) mean user experience score ≥6 [75], (3) mean system usability score ≥70% [76], (4) mean exergame enjoyment score ≥75% [77], and (5) ≥70% of (a) primary end users (eg, individuals with mild neurocognitive disorder) and (b) health care professionals perceive the DHT-enhanced training program as (i) accessible, (ii) useful, and (iii) are willing to use it in practice.

Details on recommended assessments and more elaborate examples with cutoffs for a green, amber, and red checkpoint light are provided under -item 15 and its corresponding explanation and elaboration section. Once a green light has been achieved, progress with longitudinal evaluations. Otherwise, reiterate this phase (in case of an amber light) or return to a previous phase (in case of a red light) to establish a more robust foundation for co-design as per your defined progression criteria ( 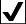-item 15).

### Phase 6: Feasibility and Longitudinal User Experience Evaluation

For the phases introduced thus far, the Co-Develop-IT checklist provides best practice recommendations that are to a large extent novel because they expand existing methodological guides and frameworks with context-specific guidance for DHT-enhanced training. Subsequent phases 6 and 7+, covering longitudinal evaluations and implementation, are more generally applicable and well covered by established guidance. Therefore, we provide (1) recommendations which established procedures to follow and (2) which additional elements to consider to make these guides and frameworks better tailored to DHT-enhanced training.

In phase 6, test the feasibility of the full DHT-enhanced training concept and the study procedures for subsequent full-scale trials, along with a more in-depth investigation of the user experience and safety of the full DHT-enhanced training concept (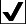-items 41 and 42). This phase is again iterative, meaning that it is repeated according to prespecified progression criteria until a solution with acceptable feasibility and user experience among primary and secondary end users is achieved (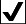-item 43).

Evaluate the feasibility of the study procedures and the DHT-enhanced training along with its user experience. We recommend doing these evaluations on the basis of the conceptual framework of Eldridge et al [78], following the terminology and recommendations of the MRC guidance [7], and considering mixed methods evaluations of both feasibility and user experience following O’Cathain et al [79] and Campbell [67]. Like in phase 5, collect complementary qualitative data that provide broader contextual information and obtain data on specific suggestions for improving the feasibility and user experience from both primary and secondary end users. Extend the abovementioned methodological guidance by the following items (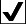-item 42):

First, a major pitfall in feasibility research is the overreliance on narrative interpretations, which may obscure unfavorable outcomes to prematurely progress to efficacy evaluations. To achieve a more transparent, objective, and reproducible approach, work with the project reference committee to establish a traffic light system–based assessment framework with predetermined progression criteria (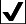-item 42a). This step should be finalized and publicly documented prior to data collection, building upon the foundational requirements detailed in 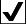-item 15.

Second, in agreement with the MIDE framework [14], systematically evaluate and report on technology performance (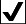-item 42b; eg, downtime, type and frequency of occurrence of technical problems, and effectiveness of supportive strategies for end users) to provide a robust basis for further iterative DHT refinements.

### Phases 7^+^: Efficacy, Effectiveness, Implementation, and Economic Evaluation

Finally, perform efficacy, effectiveness, implementation, and economic evaluations of the resulting DHT-enhanced training concept, depending on the context of the project (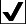-item 44). In any context, we advocate for moving beyond efficacy evaluations to address the lingering evidence-to-practice gap—which is in line with the recommendations by Owoeye et al [80]. This could be achieved by effectiveness and implementation studies following efficacy trials or with hybrid effectiveness-implementation designs [81-83]. Moreover, assess the DHT-enhanced training concept’s value (eg, risk-benefit profile and cost-effectiveness) for health care systems to provide data that allow informed decisions on the uptake of such approaches in future clinical practice guidelines.

For these evaluations, we recommend following the UK MRC guidance [7] for efficacy and effectiveness terminology and evaluations, the PRACTIS guide [21] for implementation evaluations, and the World Health Organization guide [84] for cost-effectiveness analyses (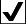-item 44). Like in phase 6, expand the abovementioned guides to accurately reflect the context of evaluations of individually tailored DHT-enhanced training concepts (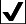-item 45) as follows.

First, while randomized controlled trials are considered the gold standard for evaluating efficacy and effectiveness [85], consider alternative study designs that may better accommodate the individualized and adaptive nature of tailored DHT-enhanced training [86] (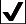-item 45a). Nahum-Shani et al [87] provide a pragmatic framework for selecting such alternative designs that are particularly relevant when interventions are multicomponent or tailored and when there are research questions regarding the timing, sequencing, or responsiveness of different intervention components. For example, a factorial design is a type of randomized trial in which 2 or more independent variables are manipulated simultaneously, allowing researchers to assess both the main effects of each factor and potential interactions between them. This makes it an efficient approach for studying multiple intervention components within a single trial. Furthermore, the sequential multiple assignment randomized trial design includes multiple randomizations, enabling participants to be rerandomized based on their response or adherence to the initial treatment, which is particularly useful when behaviors or conditions evolve slowly over time. In contrast, microrandomized trials involve frequent, often daily, randomizations to assess the short-term effects of brief interventions—such as prompts or notifications—on rapidly changing outcomes. Microrandomized trials are valuable for real-time optimization and adaptation of DHT-enhanced training. Despite the promise of these alternative approaches in addressing the need for tailoring and adaptability in digital health, they remain underused in health promotion and disease prevention interventions [87].

Second, continue with a mixed methods approach also at this stage of the project. Specifically, collect complementary participant-reported outcomes and qualitative data on perceived efficacy or effectiveness and acceptance of the implementation of primary and secondary end users and providers, as well as suggestions on how these could be (further) improved (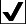-item 45b). These evaluations can provide insights not ascertainable through physiological, laboratory, clinician-reported, observer-reported, or performance outcomes alone and may thereby bring additional value and optimize the impact on clinical practice and health policy [88].

## Discussion

### Principal Findings

This methodological guideline development study gradually cocreated unifying consensus-based best practice recommendations with operational guidance for multidisciplinary expert teams coordinating projects on individually tailored DHTs and DHT-enhanced training concepts. It consists of 8 phases and is applicable to any type of end users, exercise types, intended contexts of use, and overarching goals.

### Strengths and Limitations

This project invested considerable time and effort in collaboratively working out a consensus on best practice recommendations—which was driven by a project team that spanned 5 research groups across 4 countries with 10 researchers representing a range of career stages, multidisciplinary backgrounds, and research interests. Nevertheless, we acknowledge that the key limitations of our approach were (1) the reliance on group decision-making and (2) consensus-finding procedures involving a relatively small number of researchers. Given the project’s scope, we believe that our deliberate choice to work intensively in a small group of experts is well-suited to our research aims. While a larger number of consensus group participants could have been included through more structured techniques such as the (modified) Delphi method [18], such an approach shows high reliance on researcher bias in formulating initial, preset questionnaires and relies on an often predetermined, limited number of rounds that build on elements agreed on by a supermajority only. In contrast, our process included a high frequency (biweekly) of meetings that enabled creative brainstorming and in-depth critical discussions over more than half a year to gradually propose, discuss, and develop a consensus on new best practice recommendations—which was necessary given the comprehensiveness of this work. Furthermore, the literature suggests that 10‐15 participants may suffice when the background of such a consensus process is homogeneous [18], which applies to our research goal and methods, thereby providing justification for the number of involved experts.

Nevertheless, we recognize inherent limitations of group decision-making, including the potential influence of dominant individuals, the risk of tangential discussions, and group pressure for compromise. These limitations could be mitigated through alternative methods that incorporate anonymity, controlled feedback, and statistical aggregation of responses [18]. Therefore, we advocate for future iterations to collaboratively consolidate this guideline through broader expert, patient, and public involvement. A (modified) Delphi method [18]—which is recommended [89,90] but underused [91] in the related field of the development of health research reporting guidelines—could be particularly valuable in this regard.

In addition to the above limitations, we acknowledge that all involved researchers are affiliated with European universities. Broader expert involvement with more diverse geographical, sociocultural, and institutional backgrounds—particularly from low- and middle-income countries, nonacademic sectors, and underrepresented communities—would strengthen the consolidation of such broadly applicable best practice recommendations. Nevertheless, the diversity of our consensus group (see “Disciplinary and Professional Background of the Consensus Group" section) sparked numerous critical discussions around the terminology used in different contexts, which in turn facilitated the development of a harmonized terminology that enhances the guideline’s applicability across the various interest-holders it is intended to serve.

### Comparison With Prior Work

The Co-Develop-IT guideline is an extension of the MIDE framework [14] and its refined methodology from the “Brain-IT” project [15]. The guideline integrates and expands the MRC guidance [7] and the Generative Co-Design Framework for Healthcare Innovation [12] and aspects to inform the design of new DHTs from the NASSS framework [13] and the PRACTIS guide [21]. Given that the Co-Develop-IT guideline integrated and refined these established methodological guides and frameworks, it can be seen in part as a unified approach combining the advantages of previous guides and frameworks that are expanded with refined recommendations specifically for the context of individually tailored DHT-enhanced training. However, the main novelty of this guideline lies in the introduction of multiple aspects to advance current best practices in the field.

First, the Co-Develop-IT guideline provides 5 distinct preparatory contextual research phases preceding generative codevelopment. These phases are dedicated to the structured establishment of a more robust foundation to better tailor and steer codevelopment efforts toward successful implementation. One of the key merits of the Co-Develop-IT guideline is that implementation requirements for DHT-enhanced training are considered alongside its design requirements prior to launching and continuously during the codevelopment and evaluation process. Such extensive preparatory contextual research steps extend the currently conventional approach and establish a solid foundation that facilitates the transfer of the DHT-enhanced training concept into real-world applications and clinical practice.

Second, the Co-Develop-IT guideline supports harmonizing the interests of different contributors (particularly between research, industry, health care, and end users). This is done through definitions of minimum requirements for interest-holder involvement in each project step and recommendations for systematically defining their specific roles and responsibilities.

Finally, the Co-Develop-IT guideline provides a corresponding checklist with an explanation and elaboration section to operationally guide effective implementation, which has been confirmed effective and practically feasible in 2 application examples.

### Implications for Research

While collaborative research practices are generally considered best practice, with patient and public involvement nowadays a routine section on most grant proposals, concerns have been raised about whether the costs of collaborative research practices outweigh the benefits for health research [92]. Specifically, Oliver et al [92] provided an overview of the potential risks and costs of coproduction and related participatory research practices such as co-design. Such risks and costs include, but are not limited to, increased time and resource demands, emotional and professional burdens on both researchers and interest-holders, challenges in managing conflicting expectations, risks to academic credibility, and potential dilution of research quality due to compromises made during collaboration. To overcome these risks, they recommend “[...] clarifying exactly which outcomes are required for whom for any particular piece of research [and] selecting strategies specifically designed to enable these outcomes to be achieved, and properly evaluated” [92]. Furthermore, they called for a cautious approach to codevelopment in the absence of strong evidence supporting its process and impact [92]. While there is now moderate-quality evidence that cocreation is effective by resulting in interventions that have the intended effect in the context of the secondary prevention of noncommunicable diseases [93], recent research agrees that standardized definitions and reporting frameworks for codevelopment are needed to facilitate comparisons, and ensure consistency [93,94].

The Co-Develop-IT guideline provides unifying best practice recommendations for implementing, among others, the earlier-mentioned recommendations. Specifically, it entails collaboratively—with all relevant interest-holders—(1) establishing project checkpoints consisting of a traffic light system–based assessment framework with quantitative thresholds and clear progression criteria, and (2) agreeing on the weighting of how inputs from different interest-holders should be prioritized. Furthermore, the Co-Develop-IT checklist guides standardized reporting and defines the minimum level of participatory research involvement for each item. It thereby provides operational guidance on which project steps, which interest-holders should be involved, to what extent, to ensure successful goal achievement. These strategies support projects in finding a good balance between the benefits and increased costs of collaborative research practices. Ultimately, adopting these recommendations will help advance the field to get the most out of such codevelopment projects for advancing health research that is disseminated and implemented in practical settings.

### Implications for Practice

Credible DHT-enhanced interventions and related DHTs should be evidence-based and grounded in health research that provides data to support whether these solutions achieve their intended outcomes. However, a recent review revealed a significant gap between commercial offerings and scientific validation of DHT-enhanced lifestyle interventions in the example of dementia prevention. Specifically, all reviewed studies focused on proxy measures of dementia prevention, and none demonstrated any change in the targeted condition or risk factors [95]. This observation is likely to be transferable to other use cases and highlights the need for rigorous empirical validation—rather than solely equivalence claims—to provide evidence to support the often-advertised claims on clinical effectiveness of such DHT-enhanced interventions.

Following the Co-Develop-IT guideline in future projects will result in the (1) codevelopment of well-grounded DHTs and DHT-enhanced training concepts that are well aligned with all interest-holders’ requirements, (2) provision of robust data whether the overarching goals of the specific DHT-enhanced training concept are supported by appropriate scientific evidence, and (3) proof-of-concept for the success of implementation of the resulting DHT-enhanced training concepts. Thereby, the Co-Develop-IT guideline systematically guides projects toward making a real-world impact.

### Conclusions

The Co-Develop-IT guideline provides comprehensive best practices with actionable operational guidance for establishing an appropriate balance between scientific theories and frameworks and the real-world needs of interest-holders in the establishment of individually tailored DHT-enhanced training or rehabilitation concepts. Given that this guideline integrated and refined previous methodological guides and frameworks [7,12-15,21], it can be seen as a unified approach combining the advantages of previous guides and frameworks that are expanded with refined recommendations specifically for the context of individually tailored DHT-enhanced training. Its main novelty lies in guiding the structured establishment of a more robust conceptual foundation through extensive preparatory contextual research phases, aimed at better targeting tailored codevelopment efforts toward successful implementation.

Applying Co-Develop-IT contributes to overcoming the lingering evidence-to-practice gap by consistently establishing a shared mission with relevant interest-holders and ensuring that all codevelopment steps are directed toward addressing an unmet need in (clinical) practice—ultimately promoting the practical application and impact of purpose-developed DHTs. We are providing a platform on the Open Science Framework [96] to encourage and guide future initiatives to consolidate the Co-Develop-IT guideline based on critical discussions about experiences in their application in different projects and contexts. These efforts will help the field strike a better balance between maximizing the benefits and mitigating the increased resource demands of collaborative research practices—ultimately maximizing its real-world impact.

## Supplementary material

10.2196/84163 Multimedia Appendix 1 The supplementary file provides the following elements from the Co-Develop-IT guideline package: (1) Co-Develop-IT checklist (Supplementary File 1), (2) explanations and elaborations of each checklist item with guiding examples (Supplementary File 2), (3) application example of the Co-Develop-IT guideline in the “Park-MOVE” project (Supplementary File 3), and (4) application example of the Co-Develop-IT guideline in the “Better Together” project (Supplementary File 4).

## Abbreviations

DHT: digital health technology
EQUATOR: Enhancing the Quality and Transparency of Health Research
MIDE: Multidisciplinary Iterative Design of Exergames
MRC: Medical Research Council
NASSS: nonadoption, abandonment, scale-up, spread, and sustainability
PRACTIS: Practical Planning for Implementation and Scale-Up
