# Supplementary material for: Co-Develop-IT! Unifying Methodological Guideline for the Co-Design, Development, and Evaluation of Individually Tailored Technology-Enhanced Training and Rehabilitation Concepts: Consensus Development Study and Tutorial
Source: J Med Internet Res. 2026 May 22;28:e84163. doi: 10.2196/84163 (PMC13197039; doi:10.2196/84163)
Supplement: Multimedia Appendix 1 [file jmir-v28-e84163-s001.pdf]

Supplementary Files to Publication:

Co-Develop-IT! Unifying Methodological Guideline for the **Co**-design, **Develop**ment, and Evaluation of **I**ndividually Tailored Technology-Enhanced **T**raining and Rehabilitation Concepts: Consensus Development Study and Tutorial

J Med Internet Res 2026;28:e84163; doi: 10.2196/84163

Overview

This supplementary file provides the following elements from the *Co-Develop-IT guideline package*:

1. *Co-Develop-IT Checklist* (Supplementary File 1)
2. Explanations and elaborations of each checklist item with guiding examples (Supplementary File 2)
3. Application example of the *Co-Develop-IT guideline* in the *'Park-MOVE'* project (Supplementary File 3)
4. Application example of the *Co-Develop-IT guideline* in the *'Better Together'* project (Supplementary File 4)

We recommend that the *Co-Develop-IT checklist* along with its *item-specific explanation and elaboration sections* be considered the core instruments to work with when implementing the guideline. The two application examples demonstrate the guidelines' practical applicability, relevance, and flexibility and can be used as further practical guidance when implementing the *Co-Develop-IT guideline*.

Table of Contents:

Supplementary File 1: *Co-Develop-IT Checklist* 2

Supplementary File 2: Item-Specific Explanation and Elaboration with Guiding Examples 9

2.1 Overview 9
2.2 Phase 0: Definition of Project Framework 9
2.3 Phase 1: Identification of Guidelines, Principles, Frameworks, and Theories 20
2.4 Phase 2: Determine Design and Implementation Requirements 21
2.5 Phase 3: Technology Scoping 25
2.6 Phase 4: Define Pathway & Sustainability Strategy 25
2.7 Phase 5: Co-Design, Development, Testing, and Refinement 27
2.8 Phase 6: Feasibility and Longitudinal User Experience Evaluation 33
2.9 Phases 7⁺: Efficacy, Effectiveness, Implementation, and Economic Evaluation 33

Supplementary File 3 - First Application Example: Project *'Park-MOVE'*: **35**

3.1 Overview 35
3.2 Phase 0: Definition of Project Framework 35
3.3 Phase 1: Identification of Guidelines, Principles, Frameworks, and Theories 38
3.4 Phase 2: Determine Design and Implementation Requirements 38
3.5 Prospective Phases 40
3.6 Reflection on the Use of the Co-Develop-IT Guideline in the 'Park-Move' Project 40

Supplementary File 4 - Second Application Example: Project *'Better Together'* **42**

4.1 Previous Projects Leading To 'Better Together' 42
4.2 Phase 0: Definition of The Project Framework 43
4.3 Phase 1: Identification of Guidelines, Principles, Frameworks, and Theories 45
4.4 Phase 2: Determine Design and Implementation Requirements 45
4.5 Reflection on the Use of the Co-Develop-IT Guideline in the 'Better Together' Project 45

Supplementary File 5 - Key Questions to be Considered under Checklist Items 20 - 23 **46**

References **50**

Supplementary File 1: *Co-Develop-IT Checklist*

Table 1 presents the *Co-Develop-IT Checklist*.

Table 1: *Co-Develop-IT Checklist*

(1) The minimum level of participatory research involvement was defined according to the participation choice points outlined by Vaughn and Farrah (2020) [1]. The definitions of each level have been harmonized with context of this guideline as follows:

"inform" (level 1):	Information is provided to interest-holders; usually associated with traditional research outreach to the community, but can be more participatory if interest-holders ask to be informed about particular topics.
"consult" (level 2):	Input is obtained from interest-holders; requires interest-holders to provide feedback that the researchers consider when making research decisions. This input is usually obtained with traditional qualitative research methodologies such as focus groups, individual interviews, or a (modified) Delphi approach, via consulting with individual project advisors, or via informal provision of feedback from relevant interest-holders.
"involve" (level 3):	The researchers work directly with interest-holders; requires interest-holders to provide feedback throughout the research process, for example by active membership in an advisory board.
"collaborate" (level 4):	Interest-holders are partners in research and development process; requires shared decision making and co-leadership from non-academic collaborators, which reflects the project reference committee as well as the core research team in the context of this guideline.
"empower" (level 5):	Interest-holders lead research decision making; requires that non-academic collaborators trained to be co-researchers truly lead decision making throughout the research and development process.

<table>
<tr><th rowspan="2">Item:</th><th rowspan="2">Criterion:</th><th colspan="3">Contextual Requirements:</th></tr>
<tr><th>Item <u>Relevance</u> with "Short-Track" Option</th><th><u>Minimum level of participatory research involvement</u> (1)</th><th><u>Responsible interest-holder(s) for decision making</u></th></tr>
<tr><td colspan="5">Phase 0: Definition of Project Framework

<u>Aims of this phase:</u>
I. To define non-negotiable key elements of a project, such as the goal(s), target population(s), and the overall multidisciplinary context. These non-negotiable key elements provide a framework within which the co-design and development work takes place and set clear boundaries on the freedom of this process towards fulfilling a projects' bigger purpose (i.e., to iteratively conceptualize, co-design, develop, test, refine, evaluate, and implement an individually tailored digital health technology (DHT)-enhanced training or rehabilitation concept to maintain and/or improve a specific outcome domain in a specific target population and context).
II. To define requirements on interest-holder involvement, resources, and regulatory environment for the project.
III. To establish a project consortium including all relevant interest-holders and define their roles and responsibilities throughout the project to facilitate effective collaboration and accountability.
IV. To collaboratively agree on project checkpoints and a preliminary time plan for the project.</td></tr>
<tr><td colspan="5"><u>Phase 0.1: Define Overall Context and Goals of the Project:</u></td></tr>
<tr><td>1</td><td>Define a specific problem and knowledge gap and critically assess the relevance of addressing that problem or knowledge gap.</td><td>recommended</td><td>Level 1: inform</td><td>Principal Investigator</td></tr>
<tr><td>2</td><td>Describe the rationale for how the development of an individually tailored DHT-enhanced training or rehabilitation concept addresses the problem or knowledge gap defined in ☑-item 1.</td><td>recommended</td><td>Level 1: inform</td><td>Principal Investigator</td></tr>
<tr><td>3</td><td>Critically reflect on and make an informed decision to define the intended context of use and overarching goal of the DHT-enhanced training or rehabilitation concept to be developed.</td><td>recommended</td><td>Level 1: inform</td><td>Principal Investigator</td></tr>
<tr><td>4</td><td>Critically reflect on the relevance of all potential intended target population(s). Make an informed decision which of these target populations are of relevance to address the intended overarching goal in its context of use (☑-item 3). Consider:

4a: primary end-users (i.e., persons performing the training or rehabilitation),
4b: secondary end-users (i.e., persons supporting the delivery of the training or rehabilitation),</td><td>

recommended

beneficial</td><td>Level 1: inform</td><td>Principal Investigator</td></tr>
</table>

	4c: primary providers (i.e., persons that prescribe and/or give access the training or rehabilitation concept),<br>4d: secondary providers (i.e., company(ies), institution(s), or other party(ies) providing access to the DHTs used to deliver the training or rehabilitation),<br>4e: organizations implementing the DHT-enhanced training or rehabilitation concept, and<br>4f: communities and/or systems in which the DHT-enhanced training or rehabilitation concept should be implemented on a broader scale.	recommended<br>beneficial<br>beneficial<br>beneficial		
5	Critically reflect on and make an informed decision to define the intended **intervention type**, i.e., the type of training or rehabilitation.	recommended	Level 1: inform	Principal Investigator
6	Critically reflect on and make an informed decision to define the intended **outcome domain(s)**.	recommended	Level 1: inform	Principal Investigator
Phase 0.2: Define Requirements on Interest-holder Involvement, Resources, and Regulatory Environment for the Project:				
7	Critically reflect on and make an informed decision to define requirements for the settings and conditions under which the DHT-enhanced training or rehabilitation concept should be co-developed and tested. Identify all **required multidisciplinary interest-holders** to successfully complete the project. Consider:<br><br>7a: research institution(s),<br>7b: (healthcare) provider institution(s),<br>7c: technology developer(s),<br>7d: technology provider(s),<br>7e: legal office(s),<br>7f: additional interest-holders	 <br><br><br><br>recommended<br>recommended<br>beneficial<br>beneficial<br>beneficial<br>beneficial	Level 1: inform	Principal Investigator
8	Describe the **required resources** relevant to the overall project goals for each of the required interest-holders defined in ☑-item 7. Consider:<br><br>8a: Time<br>8b: Staff<br>8c: Funding<br>8d: Expertise<br>8e: Facilities<br>8f: Data	 <br><br>recommended<br>recommended<br>recommended<br>recommended<br>beneficial<br>beneficial	Level 1: inform	Principal Investigator
9	Synthesize key **regulatory requirements** to be followed in the project. Consider:<br><br>9a: Institutional Regulations<br>9b: Ethics and Good Clinical Practice<br>9c: Collaboration(s)<br>9d: Medical Device Regulations	 <br><br>beneficial<br>beneficial<br>beneficial<br>beneficial	Level 1: inform	Principal Investigator
Phase 0.3: Establish a Project Consortium and Define each Interest-holders' Roles and Responsibilities Throughout the Project:				
10	Check whether at least one **interest-holder representing each target population** (as defined in ☑-item 4) **and multidisciplinary context** (as defined in ☑-item 7) is available to participate in the:<br><br>10a core project team;<br>10b project reference committee; or as<br>10c project advisors (with consulting function on individual level)	 <br><br><br>recommended<br>recommended<br>beneficial	Level 3: involve	Principal Investigator
11	Describe the **available resources** for all interest-holders defined under ☑-item 10. Consider:<br><br>11a: Time<br>11b: Staff<br>11c: Funding<br>11d: Expertise<br>11e: Facilities<br>11f: Data	 <br><br>recommended<br>recommended<br>recommended<br>recommended<br>beneficial<br>beneficial	Level 3: involve	Principal Investigator

12	To mitigate potential risks of project non-completion, **check and verify** that the project team (as defined under ☑-item 10) and its available resources (as defined under ☑-item 11) meet all **contextual** (as defined under ☑-item 7) and **resource-dependent** (as defined under ☑-item 8) **requirements**. If not, recruit additional interest-holders to ensure that all requirements are met.	recommended	Level 3:<br>involve	Principal Investigator
13	Establish **collaboration agreements**, that define, among other aspects, the roles of each interest-holder, including but not limited to:<br><br>✓ degree of involvement<br>✓ responsibilities<br>✓ rights<br>✓ obligations	beneficial	Level 3:<br>involve	Principal Investigator
<u>Phase 0.4: Collaboratively Agree on Project Checkpoints as well as a Preliminary Time Plan for the Project.</u>				
14	Discuss and **refine the overall goals of the project** (as defined under ☑-items 1 - 6) until consensus **agreement** is achieved **with all interest-holders of the project consortium.**	recommended	Level 4:<br>collaborate	Core project team<br><br>AND<br><br>project reference committee<br><br>AND (optionally)<br><br>project advisors
15	Collaboratively work out project checkpoints that **define**:<br><br>15a: **project-specific subgoals** for each phase to fulfill the overall project goals (☑-item 14) aligned with the phase-specific aims defined by the *Co-Develop-IT Guideline*;<br>15b: how **achievement** of these goals (☑-item 15a) is **assessed**;<br>15c: **quality criteria** for the judgement whether the goals for each project phase (as defined under ☑-item 15a) are, considering their assessment (as defined under ☑-item 15b), achieved;<br>15d: specific **progression criteria** for all goals in each project phase (as defined under ☑-item 15a) – considering their assessment (as defined under ☑-item 15b) and quality criteria (defined under ☑-item 16c) as the target for progression to the next phase.	 <br>recommended (15a)<br>recommended (15b)<br>recommended (15c)<br>recommended (15d)	Level 4:<br>collaborate	Core project team<br><br>AND<br><br>project reference committee<br><br>AND (optionally)<br><br>project advisors
16	Collaboratively work out and **agree on a preliminary project time plan**.	beneficial	Level 4:<br>collaborate	Core project team
**Phase 1: Identification of Guidelines, Principles, Frameworks, and Theories**<br><br><u>Aim of this phase:</u> To identify any existing **guidelines or evidence-based recommendations** for the overall goals of the project and **principles**, **frameworks**, and **theories** that the project can build upon.				
17	Review and/or synthesize the current state of the evidence to **identify** any current **guidelines or evidence-based recommendations** that may be applicable in addressing the stated goals within the context of the project framework defined in Phase 0.	recommended	Level 1:<br>inform	Core project team
18	Review and/or synthesize the current state of evidence to **identify** and describe any **principles, frameworks, and theories** that might be of relevance for the project. Critically reflect on the relevance of the principles, frameworks, and theories identified and make an informed decision which of these will be followed to form the backbone for the development of the DHT-enhanced training or rehabilitation concept.	beneficial	Level 1:<br>inform	Core project team
**Phase 2: Determine Design and Implementation Requirements**<br><br><u>Aim of this phase:</u> To **elaborate a set of design and implementation requirements** for the DHT-enhanced training or rehabilitation concept to be developed, based on the guidelines or evidence-based recommendations (☑-item 17) and taking into account the principles, frameworks, and/or theories (☑-item 18). To do so, integrate the findings from **three core viewpoints**:<br>(1) A synthesis of **published scientific evidence**;<br>together with the findings from performing qualitative research on the perspectives of:<br>(2) the intended **primary end-users**, and<br>(3) the intended **secondary end-users and other relevant interest-holders**.				

19	Generate **user models** for:<br><br>19a: primary end-users; and<br>19b: secondary end-users.	 <br>recommended<br>beneficial	Level 3:<br>involve	Core project team
20	Define **requirements for core components of the DHT-enhanced training or rehabilitation concept**; considering:<br><br>20a: training or rehabilitation location and time<br>20b: training or rehabilitation components (i.e., exercise and training variables);<br>20c: personalization of the training or rehabilitation;<br>20d: individualized progression of the training or rehabilitation.<br>20e: safety;<br>20f: familiarization with the training or rehabilitation concept and DHTs;<br>20g: instructions;<br>20h feedback and rewards;<br>20i: supervision and guidance;	 <br>recommended<br>recommended<br>beneficial<br>recommended<br>recommended<br>beneficial<br>beneficial<br>beneficial<br>beneficial	Level 3:<br>involve	Core project team
21	Define **hardware requirements** for DHTs to implement the training or rehabilitation concept. Elaborate design requirements to:<br><br>21a: promote accessibility by all target populations;<br>21b: promote user experience by all target populations;<br>21c: mitigate or deal with potential technical problems.	 <br>recommended<br>recommended<br>recommended	Level 3:<br>involve	Core project team
22	Define **environmental requirements for training or rehabilitation locations**. Consider:<br><br>22a: training or rehabilitation equipment;<br>22b: space requirements;<br>22c: connectivity (e.g., local area network (LAN), Wi-Fi, Bluetooth);	 <br>recommended<br>beneficial<br>beneficial	Level 3:<br>involve	Core project team
23	Define **software requirements** for DHTs to implement the training or rehabilitation concept. Elaborate design requirements to:<br><br>23a: allow implementation of defined intervention type;<br>23b: promote accessibility by all target populations;<br>23c: promote user experience by all target populations;<br>23d: mitigate or deal with potential technical problems;<br>23e: provide instructions;<br>23f: provide feedback and rewards;<br>23g: promote personalization of training or rehabilitation;<br>23h: promote individualized progression of training or rehabilitation;<br>23i: allow monitoring adherence and training or rehabilitation fidelity.	 <br>recommended<br>recommended<br>recommended<br>beneficial<br>beneficial<br>recommended<br>beneficial<br>recommended<br>beneficial	Level 3:<br>involve	Core project team
**Phase 3: Technology Scoping**<br><br>Aim of this phase: To (i) **identify and critically appraise current (partial) solutions** that are available in research and on the market that align with the overall goals of the project (as determined in Phase 0); (ii) assess each identified solution against the design and implementation requirements defined in Phase 2 in order to gain a **comprehensive understanding of the current state of knowledge** and, in particular, to identify trends and areas for further improvement.				
24	24a: **Identify** any existing (partial) solutions (i.e., training or rehabilitation concepts, DHTs) available in research and the market, and document their key features and functionalities.<br>24b: **Critically appraise** all identified solutions (☑-item 24a) using techniques such as the SWOT (Strengths, Weaknesses, Opportunities, and Threats) matrix.<br>24c: Assess the extent to which each identified solution (☑-item 24a) **meets the design and implementation requirements** defined in Phase 2, and highlight any gaps or areas where these solutions fall short.<br>24d: Conduct a trend analysis based on the findings from ☑-items 24a to 24c. **Identify emerging trends** in the research field and technological sector, analyze which developments may be relevant to better fulfill the design and implementation requirements, and critically reflect on insights into the direction of future research and technological advancements.	recommended<br><br><br>beneficial<br><br><br>recommended<br><br><br><br>beneficial	Level 4:<br>collaborate	Core project team

**Phase 4: Define Pathway & Sustainability Strategy**<br><br>Aim of this phase: First, based on the findings of phases 1 to 3, decide whether the project will take the **path** of (i) developing a novel DHT-enhanced training or rehabilitation concept from scratch or (ii) building on and further developing existing solutions. Second, work out a **sustainability strategy**.				
25	Based on integrating the findings from Phases 0 - 3, clarify the project **starting point and choice of path:**<br><br>(i) develop a DHT-enhanced training or rehabilitation concept from scratch (→ follow **Path 1**), or<br>(ii) further develop existing DHT-enhanced training or rehabilitation concept and/or DHTs (→ follow **Path 2**).<br><br>In the latter case, clearly define a corresponding starting point for the project by elaborating and critically reflecting any preparatory steps that led to the starting point.	recommended	Level 1:<br>inform	Core project team
26	Develop a **sustainability strategy** to ensure that the solution (DHT-enhanced training or rehabilitation concept) to be (further) developed will be made available to the intended target populations (defined under ☑-item 4) and remain available after the project's completion	recommended	Level 4:<br>collaborate	Core project team
**Phase 5: Co-Design, Development, Short-Term Testing, and Refinement**<br><br>Aim of this phase: To iteratively (i) **co-design and develop prototypes** of the DHT-enhanced training or rehabilitation concept, (ii) perform **short term user experience and safety testing and validation** of prototypic components of DHT-enhanced training or rehabilitation concept, (iii) **refine** these prototypes through further co-design and development, and (iv) iteratively repeat these steps until an **acceptable solution** (= all components of the DHT-enhanced training or rehabilitation concept have been successfully validated and have acceptable user experience and good safety) is achieved.<br><br>Path 1:<br>**Develop** DHT-enhanced training or rehabilitation concept **from scratch**:<br>Path 2:<br>**Further develop** existing DHT-enhanced training or rehabilitation concept(s) and/or DHTs:				
Phase 5.1: Build a Framework for the Co-Design:				
27	Integrate the findings from the previous phases to develop a framework of:<br><br>27a already **congruent aspects** for the DHT-enhanced training or rehabilitation concept; and<br>27b define which remaining **aspects** for the DHT-enhanced training or rehabilitation concept **require co-design**.	 <br>recommended<br><br>recommended	Level 3:<br>involve	Core project team
28	For each of the aspects defined under ☑-item 27b, collaboratively work out and **define** the **weighting of** how **input from different interest-holders** should be prioritized. This is particularly relevant when making design decisions in the case of potential incongruent findings or conflicting results between the three core viewpoints from which findings should be integrated.	beneficial	Level 4:<br>collaborate	Core project team<br><br>AND<br><br>project reference committee<br><br>AND (optionally) project advisors
Phase 5.2: Co-Design Workshops:				
29	Set the stage to **facilitate reflective dialogue** and **productive workshops**.<br><br>29a Share the rationale and overall goals of the project with all co-design workshop participants;<br>29b Share preparatory findings of the project with all co-design workshop participants with a specific focus on the elaborated design and implementation requirements;<br>29c Clarify the planned procedures within the co-design workshops<br>29d Clarify the expectations of the co-design workshops and the role and expectations of each contributing workshop participant;<br>29e Establishing trust, empathy and comfort with all participants to share their personal viewpoints in the co-design group.	 <br>recommended<br><br>recommended<br><br><br>recommended<br>beneficial<br><br>beneficial	Level 4:<br>collaborate	Core project team

30	Present **existing (parts of) solutions** that the project builds on (as defined under ☑-item 27).	Path 1:<br>not applicable<br><br>Path 2:<br>recommended	Level 1:<br>inform	Core project team
31	For all aspects defined under ☑-item 27, **co-design conceptual prototypes** of the DHT-enhanced training or rehabilitation concept**.** Consider the weighting of how input from different interest-holders should influence design decisions (as defined under ☑-item 28).	recommended	Level 3:<br>involve	Core project team
Phase 5.3: Data Synthesis:				
32	**Analyze the data** obtained from the workshops and integrate the findings from all co-design workshops.	recommended	Level 1:<br>inform	Core project team
33	**Integrate the findings** from all co-design workshops and **rank-order complementary solutions** for the same aspects of the DHT-enhanced training or rehabilitation concept from the different workshops.<br><br>Comment on Responsibility: In case of (i) incongruent findings or conflicting results between different workshops as well as (ii) when complementary solutions for the same aspects of the DHT-enhanced training or rehabilitation concept from different workshops are available; the core project team present these results to the project reference committee and they make consensus-guided decisions on how to integrate these observed results into project-specific decisions on the design of the DHT-enhanced training or rehabilitation concept. Ideally, advice from project advisors is also considered.	beneficial	Level 4:<br>collaborate	Core project team<br><br>AND<br><br>project reference committee<br><br>AND (optionally) project advisors
34	Derive a list of DHT development tasks to be completed.	recommended	Level 1:<br>inform	Core project team
Phase 5.4: Development:				
35	**Develop testable prototypes** of the DHT-enhanced training or rehabilitation concept.	recommended	Level 4:<br>collaborate	Core project team
Phase 5.5: Short-Term Testing and Validation:				
36	Perform **short-term user experience and safety testing** on components of the DHT-enhanced training or rehabilitation concept according to the requirements defined under ☑-item 15b.	recommended	Level 3:<br>involve	Core project team
37	**Validate** the **relevant components** of the DHT-enhanced training or rehabilitation concept according to the requirements defined under ☑-item 15b.	beneficial	Level 1:<br>inform	Core project team
38	**Check** whether the **quality criteria** defined under 15c have been reached.	recommended	Level 1:<br>inform	Core project team
Phase 5.6: Iterative Refinements:				
39	**Share the outcomes** from short-term testing with all co-design workshop participants.	recommended	Level 1:<br>inform	Core project team
40	**Iteratively** repeat the procedures described under ☑-items 30 to 39 according to the specific subgoals and progression criteria defined under ☑-item 15a and 15d **until an acceptable solution** (= all components of the DHT-enhanced training or rehabilitation concept have been successfully validated and have acceptable user experience and good safety levels) **is achieved**.	recommended	Level 3:<br>involve	Core project team

Phase 6: Iterative Feasibility and Longitudinal User Experience Evaluation

Aim of this phase: To iteratively **test the feasibility** of the full DHT-enhanced training concept and the study procedures for subsequent full-scale trials, along with a more in-depth investigation of the **user experience and safety** of the full DHT-enhanced training concept. This phase is again iterative, meaning that it is to be repeated according to prespecified progression criteria until a solution with acceptable feasibility and user experience among primary and secondary end-users is achieved.

41	Evaluate **feasibility** of the study procedures and the DHT-enhanced training or rehabilitation along with its **user experience**. We recommend to do these evaluations on the basis of the conceptual framework of Eldridge et al. (2016) [2], following the terminology and	recommended	Level 3:<br>involve	Core project team

	recommendations of the Medical Research Council guidance [3], and considering mixed-method evaluations of both feasibility and user experience following O'Cathain et al. (2015) [4] and Campbell (2024) [5].			
42	Extend the above-mentioned guidelines by the following ☑-items to accurately reflect the context of evaluations of individually tailored DHT-enhanced training or rehabilitation concepts:<br><br>42a: Define and publish a traffic light system-based assessment framework with clear benchmarks and predetermined progression criteria before starting data collection (relates to ☑-item 15).<br>42b: Expand on evaluations of and reporting on technology performance (e.g., downtime, type and frequency of occurrence of technical problems, effectiveness of strategies to help end-users deal with these, etc.).	beneficial	Level 3:<br>involve	Core project team<br><br>AND<br><br>project reference committee<br><br>AND (optionally) project advisors
43	**Iteratively repeat** the procedures described under ☑-items 41 and 42 according to the specific subgoals and progression criteria defined under ☑-item 15a and 15d until an acceptable solution (= all predetermined criteria for *"acceptable"* feasibility and user experience are met) is achieved.	recommended	Level 3:<br>involve	Core project team
**Phases 7⁺: Efficacy, Effectiveness, Implementation, and Economic Evaluation**<br><br>Aim of this phase: To evaluate the efficacy, (cost-)effectiveness and implementation of the resulting DHT-enhanced training or rehabilitation concept.				
44	For all following procedures in phases 7^+, we recommend following the UK Medical Research Council guidance [3] for efficacy and effectiveness evaluations, the PRACTical planning for Implementation and Scale-up guide [6] for implementation evaluations, and the World Health Organization guide [7] for cost-effectiveness analyses.	recommended	Level 3:<br>involve	Core project team
45	Expand the above-mentioned guidelines to accurately reflect the context of evaluations of individually tailored DHT-enhanced training or rehabilitation concepts:<br><br>45a: While randomized controlled trials are considered the gold standard for evaluating efficacy and effectiveness [8], consider **alternative study designs** that may better accommodate the individualized and adaptive nature of tailored DHT-enhanced training or rehabilitation [9].<br>45b: Continue with **mixed methods approach**. Collect complementary participant-reported outcomes and qualitative data on perceived efficacy/effectiveness and acceptance of the implementation of primary and secondary end-users, as well as suggestions on how these could be (further) improved.	beneficial	Level 3:<br>involve	Core project team

Supplementary File 2: Explanation and Elaboration of each Item of the *Co-Develop-IT Checklist* with Guiding Examples

2.1 Overview

Below, we provide item-specific explanations and elaborations, along with illustrative examples. These examples will cover different target populations, intended contexts, and application scenarios to demonstrate the relevance and specifics of each item in various project scopes. However, examples only serve for illustration purposes and do not limit the applicability of the *Co-Develop-IT guideline*, which covers any type(s) of end-user(s), exercise type(s), intended context(s) of (e.g., primary healthcare, community health services, digital health), and overarching goal(s) (e.g., health promotion, primary through tertiary disease prevention; including rehabilitation).

2.2 Phase 0: Definition of Project Framework

The initial phase of the process of applying the *Co-Develop-IT guideline* is dedicated to:

i. defining *non-negotiable key elements* of the project (i.e., Phase 0.1);
ii. defining *requirements on interest-holder involvement*, resources, and the regulatory environment for the project (i.e., Phase 0.2);
iii. *establishing a project consortium* including all relevant interest-holders and *defining their roles and responsibilities* throughout the project for effective collaboration and accountability (i.e., Phase 0.3); and,
iv. collaboratively agreeing on specific *subgoals and progression criteria* as well as a preliminary project time plan (i.e., Phase 0.4).

These steps are to establish a collaboratively agreed-upon framework within which the project should take place.

Phase 0.1: Define Overall Goals of the Project:

The first subphase relates to initiating the project by outlining its:

- ✓ *overarching goals and broader intended context* (*Co-Develop-IT checklist* ☑-item 3; e.g., which structures or functions the training or rehabilitation should impact, type and multidisciplinary expertise of involved institutions),
- ✓ *target population(s)* (☑-item 4; e.g., individuals at risk for or living with a specific disease or condition as primary end-users along with healthcare professionals and relatives/supporters as secondary end-users,
- ✓ *intervention type* (☑-item 5; e.g., DHT-enhanced multidomain (physical, motor, and cognitive) training or rehabilitation), and
- ✓ *targeted outcome domain* (☑-item 6; e.g., health-related quality of life).

This step lies in the responsibility of the Principal Investigator together with their (research) team of the project.

While the *Co-Develop-IT checklist* defines the minimum level of participatory research involvement as *"inform"* at this stage, it later mandates that the items of this section be discussed and refined involving all interest-holders of the project consortium until a consensus agreement is achieved (☑-item 14). However, to identify and recruit relevant interest-holders to be part of the project consortium (Phase 0.2), a broader definition of the project's

context and goals is needed. Therefore, at this stage, the items require the drafting of the overall goals of the project, which are to be refined with relevant interest-holders once a project consortium has been established. This structured approach of interest-holder involvement in the establishment of a research plan ensures that the appropriate interest-holders are identified and involved in the project and that the overall project goals are in line with patient and public needs.

This step is critical to delineate a clear path to follow and set boundaries for the freedom of the subsequent process toward fulfilling a project's larger purpose (i.e., to iteratively co-design, develop, test, refine, and evaluate an individually tailored DHT-enhanced training or rehabilitation concept to maintain and/or improve a specific outcome domain in a specific target population and context). Therefore, make sure these definitions are backed on a clearly defined theoretical rationale for how the development of an individually tailored DHT-enhanced training or rehabilitation concept can address a specific problem or knowledge gap (☑-items 1 and 2).

However, it is equally important to leave sufficient room for innovations and novel ideas that may arise during the co-design process to be integrated. To do so, no specifics such as exercise or training variables, specific DHTs to be used to implement the training, or any design elements of the DHTs and the training or rehabilitation are to be defined at this stage. Once a project consortium is established (phase 0.4), these initial definitions are to be refined and agreed on collaboratively with all relevant interest-holders gap (☑-item 14) – thereby providing a structured approach to ensure that the overall project context and goals are backed both by both a theoretical rationale and real-world patient and public needs.

Item 1: Problem and Knowledge Gap:

As in every research project, it is central to define a specific problem or knowledge gap to ensure that the project's goals are grounded in contributing to extending existing scientific knowledge and/or developing solutions that address an unmet need.

A *problem* might, for example, be that the majority of individuals with metabolic disorders (including obesity and diabetes) fail to meet general physical activity recommendations for the maintenance of health [10-13] despite (i) the evidence recommending physical activity or training or rehabilitation as a potential disease-modifying intervention in metabolic disorders (including obesity and diabetes) [14-16]; and (ii) the urgent need for effective measures to mitigate the challenges for aging societies and healthcare systems posed by the persistent increase in the prevalence and burden of metabolic disorders [17].

A *knowledge gap* could be whether a purpose-developed and individually tailored training or rehabilitation concept focusing on the ecological validity of the training or rehabilitation provides superior efficacy when directly compared to a one-size-fits-all approach or conventional exercise(s) for improving brain health in individuals with neurocognitive disorders [18].

Clearly identifying such problems or knowledge gaps ensures that the research is focused and relevant, grounded in contributing to extending existing (scientific) knowledge and/or developing solutions that specifically address an unmet need, ultimately aiming to produce meaningful and impactful outcomes. In addition, given the significant investment in resources required to complete a project in accordance with the *Co-Develop-IT guideline*, we

recommend a critical assessment of the relevance of addressing the identified problem or knowledge gap to ensure appropriate resource allocation.

Item 2: Rational for Individually Tailored DHT-enhanced Training or Rehabilitation Concept:

A clear rationale for how the development of an individually tailored DHT-enhanced training or rehabilitation concept could potentially address the problem or knowledge gap defined in item 1 is critically important to ensure that the project objectives are well-founded. This rationale should articulate the theoretical and empirical basis, proposed mechanisms of action, and anticipated benefits for how an individually tailored DHT-enhanced training or rehabilitation concept is expected to address the problem and knowledge gap defined in ☑-item 1. As an example, according to the *'Beyond "Just" Fun of Exergames Framework'* [19], exergame-enhanced interventions can be designed to provide superior adherence to relevant principles of behavior-change, neuroscience, and exercise science, thereby contributing to the maintenance and improvement of overall health in ways that conventional forms of physical activity and therapy may not. Furthermore, it introduces a progressive approach centered around the stepwise introduction of new exergame features to help people overcome physical inactivity and/or sedentary behavior all the way to sustained and purposeful training or rehabilitation to consolidate such behavior changes. Thereby, the purposeful and systematic introduction of exergame features – such as immersion, game narrative, feedback, reward, challenge, and progression mechanisms – provides a unique advantage to enhance the ecologically validity of the exercises and for tailoring and progressing interventions beyond conventional approaches. [19]

Item 3: Intended Context of Use and Overarching Goal of Training or Rehabilitation Concept:

Clearly defining the intended context of use and overarching goal of the DHT-enhanced training or rehabilitation concept at the outset of the project is crucial for aligning the project's objectives and ensuring coherence throughout its co-design and development phases.

Intended Context of Use: The intended context of use defined the environment in which the DHT-enhanced training or rehabilitation concept is intended to be implemented in the long-term. Examples of such settings include primary healthcare, community health services, telemedicine, or similar environments. The intended context of use may significantly influence and/or restrict the design of the DHT-enhanced training or rehabilitation concept. For instance, if the training or rehabilitation is intended to be an adjunct to usual care within primary healthcare, the type, content, and volume of usual care interventions need to be considered. This consideration is central to inform the structuring and integration of the training or rehabilitation concept within the existing care framework and build a robust basis for transforming existing healthcare structures. In the context of Parkinson's disease rehabilitation, as an example, existing treatments such as medications, physical or speech therapy, or functional brain stimulation might limit the available resources (e.g., time) of the intended target populations (i.e., Parkinson's disease patients and healthcare professionals) or may require alignment with the training or rehabilitation concept (e.g., timing of training or rehabilitation delivery in relation to Levodopa medication or functional brain stimulation).

Overarching Goal: The overarching goal of the training or rehabilitation concept defines the primary purpose of the training or rehabilitation and provides a framework to outline specific subgoals regarding which structures and/or functions the training or rehabilitation should impact. It could, for example, aim at health promotion or

disease prevention and management (i.e., the primary, secondary, or tertiary prevention of a specific disease or disorder) through the implementation of structured DHT-enhanced training or rehabilitation programs.

Defining these key aspects helps ensure that the DHT-enhanced training or rehabilitation concept is designed with a clear understanding of the context in which it will be implemented and/or evaluated. Moreover, these clear definitions will guide subsequent decisions, such as defining relevant target populations (☑-item 4), determining the multidisciplinary context in which the co-design and development should take place (☑-item 7), or selecting an appropriate comparator when evaluating efficacy and/or effectiveness in later stages of the project. By defining the intended context of use and the overarching goal of the training or rehabilitation concept at an early stage, all subsequent project actions can be aligned consistently with these.

Item 4: Target Population(s):

To facilitate future implementation, for example in clinical practice or targeted communities, it is important to define (and later involve, see ☑-item 10 and forward) all relevant target populations that will be relevant for the implementation of the training or rehabilitation concept to be developed. Accordingly, our definition of the target population(s) is aligned with the recommendations of the **PRACT**ical planning for **I**mplementation and **S**cale-up (PRACTIS) - Guide [6]. On this basis, we recommend critically reflecting on the relevance of potentially intended target population(s) and make an informed decision on all of the following levels:

- Individual Level:
    - Primary end-users (i.e., persons performing the training or rehabilitation; item 4a)
    - Secondary end-users (i.e., person(s) supporting the delivery of the training or rehabilitation; item 4b)
- Provider Level:
    - Primary providers (i.e., persons prescribing or giving access to the training or rehabilitation concept; item 4c)
    - Secondary providers (i.e., companies or institutions providing access to the technologies used to implement the training or rehabilitation; item 4d)
- Organizational Level:
    - Organizations implementing the training or rehabilitation concept (item 4e)
- Community and/or Systems Level:
    - Communities and/or systems in which the training or rehabilitation concept should be implemented (item 4f)

Should certain intended target populations be deemed irrelevant for a particular project, a rationale should be provided to justify their exclusion. This approach ensures that all relevant interest-holders are considered, promoting successful implementation and scalability of the DHT-enhanced training or rehabilitation concept.

Item 5: Intervention Type

To ensure that the goals of the project align with the project team's expertise, it is important to critically reflect on and make an informed decision to define the intended intervention type at the outset. This involves specifying the type of DHT-enhanced training or rehabilitation, such as physical (e.g., cardiorespiratory, strength), motor (e.g., motor learning, balance), cognitive (e.g., compensatory or restorative cognitive training or rehabilitation), physical-cognitive, motor-cognitive, or multi-domain DHT-enhanced training or rehabilitation; definitions see [19]. Clearly defining the intervention type helps in aligning the project's goals with the specific skills, knowledge, and experience of the team, ensuring that the intervention is designed and implemented effectively. Importantly, no

specifics regarding the design, content, or characteristics of the training or rehabilitation, such as exercise or training variables [20], are to be defined at this stage, as those decisions should not only be based on scientific evidence but also consider the perspectives of intended end-users and are therefore part of Phase 2.

Item 6: Outcome Domain(s)

The outcome domain(s) refer to the specific area that the training or rehabilitation is designed to impact. These outcomes may include but are not limited to global or domain-specific physical and/or cognitive performance, general disease progression (i.e., in the context of secondary prevention), walking ability, balance, risk of falls, activities of daily living (ADL), cardiorespiratory fitness, or quality of life. Critically reflecting and defining the intended outcome domain(s) at the very beginning of the project is important to provide a clear framework for the project and to guide the alignment of all relevant subsequent iterative steps towards the goal of impacting this/these outcome domain(s). This clarity helps in selecting appropriate evaluation tools and in focusing the co-design and development efforts to tailor the training or rehabilitation to achieve the desired impact.

Phase 0.2: Define Requirements on Interest-Holder Involvement, Resources, and Regulatory Environment for the Project:

The second subphase involves the identification of requirements and needs for a complete project consortium that can successfully perform the project. This includes defining:

(i) contextual requirements and required multidisciplinary interest-holders (☑-item 7),

(ii) required resources for each interest holder (☑-item 8; e.g., time, staff, funding, expertise, facilities, and data resources), and

(iii) regulatory requirements (☑-item 9) to successfully complete the project (e.g., institutional, ethics, and good clinical practice regulations, and requirements for collaboration agreements).

Item 7: Contextual Requirements:

Critically reflecting on and making an informed decision to define requirements for the settings and conditions under which the training or rehabilitation concept should be developed and tested, and identifying all required multidisciplinary interest-holders to successfully complete the project is crucial for ensuring that the project will be grounded in a realistic and competent environment. The interest-holders should ideally include:

- **Research Institution(s):** Academic or research institutions that will provide the scientific and methodological expertise necessary for the project.
- **(Healthcare) Provider Institution(s):** Healthcare facilities or organizations that will facilitate access to the target population, provide clinical insights and support, and implement the resulting DHT-enhanced training or rehabilitation concept.
- **Technology Developer(s):** Companies or teams (may be within the research institution(s)) responsible for DHT software and/or hardware development.
- **Technology Provider(s):** Companies or teams (may be within the research institution(s)) responsible for supplying the technological infrastructure (software, hardware, computing power, etc.) and providing support for the implementation of DHT-enhanced training or rehabilitation (e.g., the installation of device(s) and ongoing technological maintenance and support for DHTs).
- **Legal Office(s):** Legal experts who will ensure that all aspects of the project comply with relevant regulations and ethical standards and support efforts toward getting the final DHT-enhanced training or

rehabilitation concept (i.e., the conceptual software) and/or related DHTs (i.e., software and/or hardware) commercialized and certified as medical device.

- **Additional Interest-holders:** Depending on the project's scope, additional interest-holders might include patient advocacy groups, funding bodies, policy makers, and end-user representatives.

Item 8: Required Resources

To ensure the successful execution of the project, it is crucial to describe the required resources relevant to the overall project goals for each of the interest-holders involved. This includes detailing the following aspects:

1. **Time**: The amount of time each required interest-holder (as defined under ☑-item 8) should be available to commit to the project.
2. **Staff**: The personnel required by each interest-holder.
3. **Funding**: The financial resources required for the project.
4. **Expertise**: The specific skills and knowledge required from each interest-holder including technical, clinical, and research expertise.
5. **Facilities**: The physical and technological infrastructure required to execute the project, including laboratories, clinical settings, technological platforms, and data computing facilities.
6. **Data**: Requirements for effective data management practices should be outlined, including data collection, storage, security, and sharing protocols. Additionally, data resources, if any, required for successful execution of the project should be outlined, such as existing datasets, patient records, or research findings that the project might profit from.

Item 9: Regulatory Requirements

Synthesizing key regulatory requirements is helpful to ensure that the project complies with all relevant legal and ethical standards. This includes addressing the following aspects:

1. **Institutional Regulations**: Identify the specific regulations and policies of the institutions to be adhered to in the project. This may include obtaining necessary approvals from institutional review boards, following institutional guidelines for research conduct, and ensuring compliance with internal policies related to but not limited to data management, participant recruitment, collaborations, and resource use.
2. **Ethics**: Identify the specific regulations from the ethics committee regarding ethical standards for research involving human participants. This may include obtaining informed consent from participants (depending on whether data is obtained in pseudonymized or anonymized form in qualitative research), ensuring confidentiality and privacy, minimizing risks, and maximizing benefits.
3. **Collaboration(s)**: Define the regulatory requirements for collaborations with external partners. This includes defining clear requirements for establishing agreements and contracts that outline the roles, responsibilities, liabilities, and expectations of each partner (see ☑-item 14), such as addressing issues related to intellectual property, data sharing, remuneration, sharing of project findings, confidentiality, and conflict resolution.
4. **Medical Device Regulations**: Outline the steps required to patent the final training or rehabilitation concept or obtain medical device certification for the DHTs (i.e., software, hardware). This will ensure that all relevant aspects are considered during the project. This may include adhering to standards set by regulatory bodies such as the Food and Drug Administration in the United States or the European Medicines Agency in Europe. The recently published Common European Classification Grid for digital medical devices is helpful to facilitate the definition of the taxonomy and evidence requirements for assessment of DHTs and offers the possibility to have a common reference on a European level against which national classifications and evidence requirements can be mapped [21]. Key considerations include

device classification, safety and efficacy testing, cost-effectiveness evaluation, data security, labeling, and post market surveillance.

By synthesizing these regulatory requirements, the project can ensure that all legal and ethical standards will be met, thereby protecting participants, maintaining scientific integrity, and facilitating successful implementation and dissemination of the project.

Phase 0.3: Establish a Project Consortium and Define each Interest-Holders' Roles Throughout the Project:

The third subphase relates to the recruitment of relevant interest-holders to establish a project consortium based on the drafted project goals and context from section 1 and the requirements and needs for a complete project consortium outlined in section 2.

Item 10: Project Team and Interest-Holders Representing each Target Population

Ensuring that at least one interest-holder representing each target population (as defined in ☑-item 4) and multidisciplinary context (as defined in ☑-item 7) is available and involved in the project is crucial for its success. This step ensures that the project teams are constituted of members who can competently support the perspectives and needs of all relevant groups throughout the development and implementation of the training or rehabilitation concept. This entails setting up (i) a *project reference committee* (item 10b; recommended item) and including (ii) *project advisors* (item 10b; beneficial item) to continuously guide the *core project team* (item 10a; recommended item). In the case of multinational projects, it is recommended to set up a project reference team and recruit project advisors for each involved country to ensure that local and socio-cultural nuances are sufficiently considered. While their constellation should be tailored to the specific research project, we provide recommendations on key interest-holders and responsibilities of each of these three core groups in Table 3 of the main paper and below. Of note, the involvement of members of the project consortia might change throughout the conduct of a project, depending on the project-specific requirements, needs, or relevance in different phases.

The **core project team** typically consists of the team who initiated the project. As a minimum requirement, this core project team includes of the project coordinators and/or the principal investigator(s) of the project and their (research) teams (as defined under ☑-item 7a), interest-holders from the (healthcare) provider institution(s) (as defined under ☑-item 7b), and, if the technology development is not done within the research team(s), the technology developer(s) (as defined under ☑-item 7c). Given these responsibilities, and according to participation choice points for participatory research process [1], each interest-holder from the (healthcare) provider institution(s) and the technology developer(s) contributing to the core project team must have a minimum of an *"involving"* level, but might also take a *"collaborating"* or *"empowering"* role.

While the constellation of each of these project reference committees and project advisors should be tailored to the specific research project, we recommend that the **project reference committee(s)** includes:

- ✓ ≥ 1 (expert) representative of the primary end-users (e.g., patient advocacy groups or end-user representatives) (☑-item 7f),
- ✓ ≥ 1 (expert) representative of the technology provider(s) or industry (☑-item 7c), and
- ✓ ≥ 2 (expert) representatives of the (healthcare) provider institution(s) (☑-item 7b);

- 1 on organizational level (☑-item 4e), and
- 1 on individual level (☑-item 4c).

At the beginning of the project, the responsibility of this project reference committee is to collaboratively work out and define specific subgoals and progression criteria (☑-item 15) with the project core team and advise them when working out a preliminary time plan for the project (☑-item 16). In later stages of the project, the responsibility of project reference committee(s) is to guide important decisions on the project plan and execution. Most importantly, they will serve as a reference group that predetermines, together with the core project team, the weighting of how input from different interest-holders should be prioritized when making design decisions in Phase 5 (☑-item 28). Additionally, they will determine, together with the core project team, how to prioritize and weight the contribution between the three core viewpoints when making specific design decisions in the case of potential incongruent findings or conflicting results (e.g., when elaborating design and implementation requirements in Phase 2 or integrating the findings from the co-design workshops to derive a list of development tasks to be completed by the technology developer(s) in Phase 5 (☑-item 34)). Given these responsibilities, and according to participation choice points for participatory research process [1], each member of the project reference committee must have a minimum of an *"involving"* level, but might also take a *"collaborating"* or *"empowering"* role.

The **project advisors** may include representatives for each of all remaining defined levels under ☑-item 4 and 10, and may primarily consist of:

- ✓ communities and systems in which the training or rehabilitation concept should be implemented (☑-item 4e),
- ✓ legal office(s) (☑-item 7c), and
- ✓ additional interest-holders, such as funding bodies or policy makers (☑-item 7d);

depending on the project specific requirements and as defined under ☑-item 7.

The responsibility of project advisors is to be available for consulting by the core project team and provide non-binding strategic advice to the core project team on the project plan and implementation, how to deal with potential incongruent findings or conflicting results between the three core viewpoints from which findings should be integrated in each phase, and/or to take and/or refine measures to comply with recommendations on diversity and inclusion, anticipation and reflection, openness and transparency, responsiveness and adaptive change. We recommend that project advisors also include legal experts who will ensure that all aspects of the project comply with relevant regulations and ethical standards and support efforts for commercialization, patenting, or medical device certification of the DHT-enhanced training or rehabilitation concept. Given these responsibilities, and according to participation choice points for participatory research process [1], each project advisor typically has a *"consulting"* role on individual level.

Item 11: Available Resources

To ensure the feasibility of successful execution of the project (see ☑-item 12), it is important to first describe the available resources for the overall project goals for each of the interest-holders involved. This includes detailing the following aspects:

1. **Time**: The amount of time each required interest-holder (as defined under ☑-item 7) is available to commit to the project. Defining the available level of commitment of each interest-holder is key to ensure that the project recruits sufficient interest-holders to ensure the feasibility of the project's execution and that the planning aligns with the allocated time and commitment of each interest-holder and to align expectations among involved partners.
2. **Staff**: The personnel involved from each interest-holder, including their roles, responsibilities, and the level of effort they will contribute. For research staff, we recommend determining authorship roles and responsibilities early on to avoid conflicts and ensure aligned focus.
3. **Funding**: The financial resources available for the project, including budgets allocated by each interest-holder and any external funding sources. Clearly outlining the funding sources and budget allocations helps in managing financial resources effectively and ensures that all aspects of the project are adequately funded. This includes detailing any grants, institutional funding, or industry partnerships that will support the project.
4. **Expertise**: The specific skills and knowledge that each interest-holder brings to the project. This includes technical, clinical, and research expertise. Identifying the expertise of each interest-holder ensures that the project allocates specific responsibilities to those with the best available knowledge and skills, facilitating effective collaboration. This might include expertise in areas such as exergame development, clinical trial design, data analysis, and patient and public involvement.
5. **Facilities**: Describe the physical and technological infrastructure available to support the project, including laboratories, clinical settings, technological platforms, and data computing facilities. This includes detailing the availability of research labs, clinical trial sites, and technological infrastructure such as software and hardware required for developing and testing the training or rehabilitation concept.
6. **Data**: Outline current data management practices, including data collection, storage, security, and sharing protocols. List available data resources, if required, such as existing datasets, patient records, or research findings that the project might profit from. Access to relevant data is crucial for informing the design and evaluation of the training or rehabilitation concept. This may include identifying patient registries or previous research data that can be utilized to enhance the project's robustness and validity.

<u>Item 12: Check Contextual and Resource-dependent Requirements</u>

To mitigate potential risks of project non-completion, compare the available resources for all interest-holders (☑-item 11) with the required resources (☑-item 8) to identify any shortcomings that need to be addressed. Ensuring the availability of necessary resources is crucial for the smooth execution of the project and achieving its goals. Comprehensive resource mapping helps identify potential gaps, optimize resource allocation, and facilitate effective collaboration among interest-holders. In addition to the listed resources, reflecting on any relevant additional project-specific resources and potential risks that may apply to ensure a thorough risk mitigation strategy is recommended.

<u>Item 13: Establish Collaboration Agreements</u>

It is recommended to establish formal collaboration agreements in order to define the roles, responsibilities, rights, and obligations of each interest-holder. Such agreements facilitate the management of expectations and ensure accountability. It is recommended to draft comprehensive collaboration agreements that delineate the:

1. **The degree of involvement** of each collaborator, which might range on the following levels according to the participation choice points for participatory research process [1]:
    a. <u>core project team</u>: *"leading"*, *"guiding"*, *"executing"*, or *"supporting"* roles for members,

   b. project reference committee: *“involving”*, *“collaborating”*, or *“empowering”* role for each member, and
   c. project advisors: *“involving”* or *“consulting”* role for each member).
2. The specific **responsibilities** of each collaborator (e.g., which interest-holder is responsible for which project elements or work packages),
3. The **rights** of each collaborator (e.g., intellectual property, data sharing, sharing of project findings, remuneration), and
4. The **obligations** of each collaborator (i.e., in line with what has been defined under item 9; e.g., liability, compliance with regulatory requirements, confidentiality, conflict resolution).

It is also recommended to regularly revisit and update these agreements as needed to reflect any changes in the project scope or interest-holder roles. Please refer to the *Co-Develop-IT checklist* (Supplementary File 1) for requirements on the minimum level of involvement in each item.

Phase 0.4: Collaboratively Agree on Specific Subgoals and Progression Criteria as well as a Preliminary Time Plan for the Project

Finally, the fourth subphase is dedicated to collaboratively refining and agreeing, specific goals and subgoals of the project (☑-items 14 and 15a; as drafted in phase 0.1) and define *project checkpoints* (☑-item 15) – both based on a consensus process with all interest-holders of the project consortium (i.e., core project team and reference committee and (optionally) project advisors).

☑-item 14: Interest-Holders Refine and Agree on the Overall Project Goals

It is of great importance that all interest-holders involved in the project reach a consensus on the project's overall goals to facilitate the establishment of a unified direction and prevent the emergence of misunderstandings or conflicts at a later stage of the project. To this end, it is recommended that collaborative meetings or focus groups be organized in which all interest-holders contribute to discussing, refining, and reaching consensus on the definition of project goals. These sessions should address any concerns or suggestions and ensure that every interest-holder's perspective is considered. It is recommended to adopt techniques such as empathetic target group analysis [22] to foster a better understanding of the diverse needs and perspectives among interest-holders before starting the co-design or co-creation process [23, 24]. Moreover, the added value for each partner should be clearly communicated between partners to transparently exchange expectations for fruitful collaboration. The agreed-upon goals should be documented and circulated among all participants for final confirmation. This step ensures that everyone is on the same page and committed to the project's success.

☑-item 15: Define Project-specific Subgoals and Progression Criteria per Phase

Defining specific subgoals for each project phase (☑-item 15a) helps to break down the overall project goals into manageable tasks of work packages. This approach facilitates tracking progress and ensures that each phase contributes to the overall goals. We recommend working with all interest-holders to identify and define milestones with project checkpoints for each project phase. Specifically, collaboratively refine and agree on the specific goals and subgoals of the project (☑-items 14 and 15a; as drafted in phase 0.1) and define *project checkpoints* (☑-item 15) that are aligned with the overall goals of the project and its phases – both based on a consensus process with all interest-holders of the project consortium (i.e., core project team and reference committee and (optionally)

project advisors). Each project checkpoint requires a traffic light system-based assessment framework with clear benchmarks (☑-item 15c) and progression criteria (☑-item 15d) to ensure transparency in the assessment of agreed-upon goals (☑-item 15b). The project checkpoints are usually to be positioned towards the end of each phase inform whether the project:

- ✓ can progress to the next phase (= green light),
- ✓ requires further iterative loops with refinements before proceeding to the next phase (= orange light), or
- ✓ requires the establishment of a more robust foundation by regressing to a previous phase (= red light).

Descriptive examples are provided in Supplementary File 2 or in previous literature of the author team in relation to feasibility and user experience testing in preparation for a randomized controlled trial (RCT) [25, 26]. For transparency in the interpretation of project results and progression, we recommended that the traffic light system-based assessment framework be published (as part of a project protocol) or at least pre-registered (e.g., on the Open Science Framework) prior to the start of data collection.

For example, a goal of the co-design process within phase 5 of a project might be to establish interactive *“trial-run”* instructions for an exergame system that are effective and achieves *“acceptable”* usability. Effectiveness is quantified as the percentage of participants being able to correctly perform the familiarization level of the game following the instructions, with 85% success rates as *“acceptable”* effectiveness. *“Acceptable”* usability is defined by a mean score of ≥ 70 on the System Usability Scale [27] from the rating of 20 primary end-users [28]. The quality criterion could be a traffic light system-based assessment framework with quantitative thresholds on the basis of the instructions’ effectiveness outcome and adjective ratings of the System Usability Scale.

The core project team and project reference committee could, for example, agree in the following thresholds: *“okay”* effectiveness rating for success rates between 70 and 79%, *“good”* effectiveness rating for success rates between 80 and 89%, and *“excellent”* effectiveness rating for success rates between 90 and 100%. For usability, the team could, for example, agree on adopting literature-based interpretations, indicating *“okay”* usability (scores between 52 and 72), *“good”* usability (scores between 73 and 84), *“excellent”* usability (scores between 85 and 99), and *“best imaginable”* usability (score of 100) [28]. Specific progression criteria could indicate (i) requiring further iterative major refinements for *“okay”* ratings, (ii) further iterative minor refinements for *“good”* ratings, (iii) conditional progression to the next phase with minor refinements on the basis of the data available in case of the achievement of *“acceptable”* thresholds, and (iv) progression to the next phase without further refinements for *“excellent”* and *“best imaginable”* ratings and the achievement of the *“acceptable”* threshold for the effectiveness of the instructions.

☑-item 16: Collaboratively Agree on Preliminary Project Time Plan

Finally, collaboratively develop and agree on a preliminary project time plan to align interest-holder expectations regarding progress and output per unit of time. This is particularly important for harmonizing the expected rates of progress in public-private partnerships, given that public research institutions may be perceived by the industry as slow-moving *‘oil tankers’*, whereas the industry may be seen by academia as agile *‘speedboats’* rushing toward marketable outputs.

2.3 Phase 1: Identification of Guidelines, Principles, Frameworks, and Theories

Phase 1 is dedicated to identifying guidelines and evidence-based recommendations for the overall goals of the project (☑-item 17) along with principles, frameworks, or theories (☑-item 18). This phase is key to defining a robust backbone and guiding future steps of projects, particularly decisions on the design, characteristics, and content of the DHT-enhanced training or rehabilitation concept to be developed.

☑-item 17: Identify existing Guidelines and Evidence-based Recommendations

Existing guidelines and evidence-based recommendations could be clinical and/or best practice guidelines, consensus statements, or recommendations for future research derived from review articles or original publications. For example, numerous clinical and/or best practice guidelines are available for the treatment and management of specific clinical diseases and/or disorders, such as Parkinson's disease [29-33] or neurocognitive disorders [34, 35]. More recently, a global consensus on optimal exercise recommendations for enhancing healthy longevity in older adults has been published that covers health promotion as well as disease prevention and management (primary through tertiary prevention) for a wide range of different disorders and diseases [16]. For DHT-enhanced training or rehabilitation, specific evidence-based recommendations aimed at establishing a consensus on the optimal design and characteristics of exergame-based training or rehabilitation, for example, have recently been published [18, 19, 36]. These guidelines and recommendations should be taken into account when developing and evaluating novel DHT-enhanced training or rehabilitation concepts.

☑-item 18: Identify Principles, Frameworks, and/or Theories

Principles, frameworks, and/or theories that should be identified and considered could include, for example, general training principles [20, 37], neuroplasticity principles [38, 39], or principles for neurorehabilitation (.e., that integrate motor learning and brain plasticity mechanisms) [40]. Relevant frameworks might include a behavior-change framework applicable to physical activity or training or rehabilitation [41], the guided plasticity facilitation framework [42] for motor-cognitive training or rehabilitation, the adaptive capacity model [43], or the *'Beyond "Just" Fun of Exergames - Framework'* [19] that integrates the previously mentioned principles and frameworks in the context of exergames, or . Specifically, it provides evidence-based best practice recommendations for the selection and implementation of specific exergame features when using exergame technology to advance health promotion and disease prevention (primary through tertiary prevention) [19]. Relevant *theories* might include the Theory of Effort Minimization in Physical Activity [44, 45], OPTIMAL (Optimizing performance through intrinsic motivation and attention for learning) theory of motor learning [46] or the Affective-Reflective Theory of physical inactivity and exercise [47] or other behavior-change theories that support initiation and consolidation of changes in physical activity behavior [48]. Identifying relevant principles, frameworks, and theories is key to defining a robust backbone and guiding future steps of projects, particularly decisions on the design, characteristics and content of the training or rehabilitation concept to be developed.

2.4 Phase 2: Determine Design and Implementation Requirements

The overarching aim of Phase 2 is to delineate a comprehensive set of design and implementation requirements for the DHT-enhanced training or rehabilitation concept. This encompasses a range of considerations to be adhered to throughout the subsequent co-development phases, including:

I. generating *user models* (☑-item 19);
II. defining requirements for *core components* of individually tailored training or rehabilitation concepts (☑-item 20);
III. defining *environmental requirements* (e.g., training or rehabilitation equipment, space requirements, connectivity such as Wi-Fi or Bluetooth; ☑-item 21);
IV. defining *hardware* (☑-item 22) *and software* (☑-item 23) *requirements* for the DHTs.

Elaboration of the design and implementation requirements should be built on Phase 1 by integrating identified guidelines and evidence-based recommendations (☑-item 17) and taking into account relevant principles, frameworks, and theories (☑-item 18). Consider the set of core question provided in Supplementary File 5 along with the checklist facilitating DHT adoption provided by Hamasaki et al. (2025) [49] and a comprehensive (early) Health Technology Assessment to explicitly evaluate the potential value of a health technology [50] to ensure that all potentially relevant aspects are considered. All decisions are to be made by integrating the findings from three core viewpoints:

- A synthesis of published *scientific evidence*;
  together with the findings from performing qualitative research on the perspectives of:
- the *intended primary end-users*, and
- the *intended secondary end-users* and other relevant interest-holders.

The *current state of the scientific evidence* should be derived from conducting an umbrella review, meta-analysis, systematic, scoping, or narrative review based on the established levels of evidence [51]. If these types of high-level evidence were already reported in recent scientific literature, a narrative synthesis of the current state of evidence suffices. The *perspectives of intended primary and secondary end-users and all other relevant interest-holders* should be derived from conducting qualitative research (based on, e.g., semi-structured interviews, focus groups, or a (modified) Delphi approach [52]). Refer to ☑-item 29 on how to set the stage to facilitate reflective dialog and productive workshops and apply these recommendations also to the qualitative research components in this phase (☑-items 19 - 23).

At this stage of a project, we recommend conducting qualitative research with primary end-users separately from the remaining interest-holders. While all interest holders are later on recommended to co-develop solutions together, this structured approach builds the foundation for productive co-development. Specifically, it ensures that primary end-users – who might be hesitant to share their insight in the presence of field experts on DHTs or exercise and rehabilitation approaches – get their own platform to express all their insights fully and in the absence of potential power dynamics that might limit their participation [53]. By aligning data collection on design and implementation requirements to reflect the three core viewpoints from which insights are to be combined ensures that no important insights are missed. Furthermore, it fosters a better understanding of the diverse needs and

perspectives among different interest-holder groups and facilitates that all those perspectives are presented to empower all co-development participants in Phase 5 to contribute fully [23, 24].

To provide a more nuanced understanding of how inclusivity and accessibility can be ensured for all relevant intended end-users, consider examining the current state of evidence and interview/focus group transcripts through the lens of critical discourse analysis [54]. These insights should be aligned with broader frameworks for sustainable innovation and ethical design in digital health [55].

☑-item 19: Generate User Models for Primary and Secondary End-Users

It is crucial to generate detailed user models that reflect the preferences and needs of the targeted user group from a multi-disciplinary perspective, as these can help inform the design of the DHT-enhanced training or rehabilitation concept to optimize user experience. User models should be designed to ascertain the preferences and needs of the target user group from a multidisciplinary perspective, with the objective of defining criteria for optimizing the user experience in the DHT-enhanced training or rehabilitation concept to be developed.

In this regard, user models should consider general aspects such as demographic characteristics, capabilities, personal traits and hobbies, motivators for physical activity or training or rehabilitation, barriers and facilitators for physical activity or training or rehabilitation participation [56]. In the context of gamified DHTs, these considerations should specifically take into account the primary end-users' individual playstyles (e.g., achiever, explorer, or socializer) and personality characteristics (e.g., introverted to extroverted, conscientious, need for autonomy, emotional resilience, openness to experience, motivation type, risk-taking, social interaction preference) [19]. These aspects should later inform the design of the DHT-enhanced training or rehabilitation concept. As an example, in the context of exergame-based training or rehabilitation, these aspects should be considered in the game mechanics and narrative, because research point towards more familiar and plausible exergaming experience (e.g., prior experience, positive emotions) potentially being more effective in engaging older adults in exergame-based training or rehabilitation [19, 56-58].

In addition to these general aspects, user models should consider aspects specific to the project framework defined in Phase 0. These project-specific aspects might include, in the context of a clinical populations as primary end-users as an example, the clinical picture, epidemiology, risk and protective factors with a focus on potentially modifiable risk and protective factors, current prevention and treatment options, treatment preferences, motivators for treatment, or facilitators and barriers of the implementation of current prevention and treatment options or recommendations in general as well as DHT-enhanced training or rehabilitation [59].

In term of motivators for treatment, we recommend that the motivators for training or rehabilitation are coded and analyzed against the background of a theoretical framework (as defined under ☑-item 18), such as the *'Self-determination Theory'* [60]. This theory accounts for the quality of different levels of motivational regulation in physical activity or exercise settings. It is considered useful to gain a better understanding and promote training or rehabilitation motivation, enjoyment, and adherence and has demonstrated considerable efficacy in explaining exercise motivation and behavior [61-65].

☑-items 20 - 23: Define Requirements for Core Components of Training or Rehabilitation Concept

The process of defining requirements for core components of the training or rehabilitation concept (☑-item 20), hardware requirements for DHTs (☑-item 21), environmental requirements at training or rehabilitation location (☑-item 22), and software requirements for DHTs (☑-item 23) must be well aligned with the findings from the previous phases of the project. This alignment is particularly important with regards to the intended context of use and overarching goal of the training or rehabilitation concept (☑-item 3), all target populations (☑-item 4), the intervention type (☑-item 5), the intended outcome domain (☑-item 6), current guidelines and/or evidence-based recommendations (☑-item 17), the specific principles, frameworks, and/or theories forming the backbone of the development of the DHT-enhanced training or rehabilitation concept (☑-item 18), and the user models for the primary (☑-item 19a) and secondary (☑-item 19b) end-users.

To ensure that all potentially relevant aspects are considered, we recommend a set of key questions for each subitem to be taken into account in supplementary file 5. However, this list is not exhaustive and should be adapted to the specific intervention type (☑-item 5) and aligned with current guidelines and/or evidence-based recommendations (☑-item 17), the specific principles, frameworks, and/or theories forming the backbone of the development of the DHT-enhanced training or rehabilitation concept (☑-item 18). For example, the *'Beyond "Just" Fun of Exergames Framework'* [19] introduced a progressive approach centered around the stepwise introduction of new exergame features to help people overcome physical inactivity and/or sedentary behavior all the way to sustained and purposeful training or rehabilitation to consolidate such behavior changes. Its provides best practice recommendations for the purposeful use of exergames as a powerful tool to promote overall health, healthy aging, and disease management with a focus on *"serious"* exergame features, including but not limited to biofeedback, gamified assessment, mini-challenges, streak counters, rhythmic multisensory cueing, monitoring and providing multisensory feedback and rewards on exercise adherence or accuracy of movements, and manipulation of task predictability. [19]

For ☑-item 20b (training or rehabilitation components (exercise and training variables)) specifically, we recommend that all basic exercise and training variables, as defined in [20], be considered as a minimum requirement. This includes (i) exercise intensity and other aspects that influence the targeted and/or optimal training or rehabilitation load (e.g., motor and/or cognitive complexity [18]), (ii) exercise duration, (iii) type of exercise (already defined under ☑-item 5), (iv) training or rehabilitation frequency, (v) training density (more thorough definition and explanation see [66]), and (vi) training or rehabilitation duration. For motor-cognitive training or rehabilitation, further clarification might be needed by defining the type of motor-cognitive training (i.e. as defined by [42] as *'simultaneous-incorporated'* (*"Moving while Thinking"*) and *'simultaneous-additional'* (*"Thinking while Moving"*) motor-cognitive training [42]). Moreover, additional training or rehabilitation components might be relevant, such as the training or rehabilitation volume (per week and in total), specificity of training or rehabilitation, training or rehabilitation administration (i.e., performed individually, performed in a group, or mixed approaches), or the body position and involved movements of the training or rehabilitation (see [18] for an example of which training components we consider relevant for exergame-based training or rehabilitation). Finally,

adherence to principles, frameworks, and/or theories defined under ☑-item 18 must be considered, such as the general training principles [20], neuroplasticity principles [38], or principles for neurorehabilitation (.e., that integrate motor learning and brain plasticity mechanisms) [40], behavior-change frameworks applicable to physical activity or training or rehabilitation [41], the guided-plasticity facilitation framework [42] for motor-cognitive training or rehabilitation, or the *'Beyond "Just" Fun of Exergames - Framework'* [19] that integrated all the before-mentioned principles and framework in the context of exergames.

Moreover, interactions between training or rehabilitation components and key questions are critical and should also be specifically considered (e.g., safety hazards in dependence of the training or rehabilitation location (e.g., clinical setting or at home), type of instructions in dependence on the type of supervision (e.g., one-on-one, remote, or no supervision). This is particularly relevant for ☑-item 20d (individualized progression of the training or rehabilitation) together with the software requirement for DHTs (☑-item 23). Specifically, we recommend, in the context of exergame-based training or rehabilitation and in agreement with the *'Beyond "Just" Fun of Exergames Framework'* [19], defining requirements for a *"game"* module coupled with an *"instructor"* module and a separate *"evaluation"* module. The following recommendations on the instructor and evaluation module also extends to all other forms of DHTs, whereas the game module might be differently framed (e.g., *"delivery module"*, *"user module"*, or similar) given the potential lack of gamification (and interactivity) on other forms of DHTs (such as electronic or mobile health applications).

For the *game module*, software requirements should be defined to allow individually adjusting training or rehabilitation demands in real-time according to in-session metrics (e.g., game performance metrics, physiological response, or rating(s) of perceived exertion). For the *instructor module* software requirements should be defined how the exercise instructor(s) (i.e., secondary end-users, such as therapists, behavior change coaches, medical doctors, researchers, or the primary end-users in case of self-regulated training or rehabilitation) can be optimally designed to support creating and/or managing exercise/training or rehabilitation plans, training periodization and programming, mini exergame bundles, goal setting and monitoring of adherence and fidelity to the exercise/training or rehabilitation plan, (remote) supervision, and/or sending out reminders. For the *evaluation module*, requirements on the type and design of gamified assessments concerned with the regular verification whether the exercise/training or rehabilitation stimulus is sufficient to induce the desired skill-related changes (in the context of exergaming) or near- and far-transfer effects (in the context of exergame-based training or rehabilitation and an exergame-enriched lifestyle) and how this data can be used to inform decisions on individually tailoring, adjusting and progressing the exercises in the instructor module should be worked out.

Based on the project checkpoints, Phase 2 evaluations should be repeated iteratively (= amber light; e.g., due to insufficient coverage of relevant themes as per feedback from the project reference committee) until the green light criteria for project progression are met, at which point the project can progress to Phase 3. If red light criteria are met (e.g., due to a strong mismatch between defined project goals (☑-item 14) and "real-world" needs as per findings from the qualitative studies), it is suggested that the project requires a more robust foundation to be established by returning to Phase 2, as per the progression criteria defined in Phase 0 (☑-item 15).

2.5 Phase 3: Technology Scoping

☑-item 24: Identify and Critically Appraise Current Solutions and Trends

The third phase focuses on identifying (☑-item 24a) and critically appraising (☑-item 24b) existing (partial) DHT solutions in both research and the market that align with the project's overall goals (Phase 0). Apply techniques such as trend analysis and the SWOT (acronym for Strength, Weakness, Opportunities, and Threats) matrix [56] to gain a comprehensive understanding of the current state of knowledge and to identify areas for further improvement. Moreover, assess each identified solution against the design and implementation requirements defined in Phase 2 (☑-item 24c). Understanding the strengths, limitations, and areas for improvements of current solutions in relation to the design and implementation requirements is essential for building robust foundation for advancing the field with credible, innovative, and evidence-based DHT-enhanced training or rehabilitation concepts. Finally, identify and analyze emerging trends (☑-item 24d) to ensure that the project identifies and integrates relevant technological advancement that may be relevant to better fulfill the design and implementation requirements. This is especially important for rapidly evolving technological opportunities, such as augmented or mixed reality, artificial intelligence, bio- or neurofeedback, and brain-computer interfaces [19].

2.6 Phase 4: Define Pathway & Sustainability Strategy

The aim of Phase 4 is to make a strategic decision on the project's development path (☑-item 25) and establish a sustainability strategy for the DHT-enhanced training or rehabilitation concepts to be (further) developed (☑-item 26).

☑-item 25: Determine Starting Point and Choice of Path

This phase first involves determining, on the basis of the findings of Phases 1 - 3, whether the project strives to develop a novel DHT-enhanced training or rehabilitation concept from scratch (*Path 1*) or further develops and builds on existing solutions (*Path 2*).

A purpose-developed software builds the heart of every DHT, whereas the conceptual decisions and algorithmic decision trees provided in a training or rehabilitation concept build the heart of a DHT-enhanced training or rehabilitation concept [19]. Therefore, to maximize scalability and transferability to other application scenarios or use cases, focus on developing training or rehabilitation concepts and software to implement the training or rehabilitation while relying on (i) well-established, off-the-shelf hardware and (ii) ensuring the software is universally applicable with different hardware peripherals. This adheres to the *"training [instead of product] first"* approach [36] and facilitates the sustainability strategy, as it reduces the complexity of providing necessary equipment and improves scalability.

☑-item 26: Work out Sustainability Strategy

We recommend that a sustainability strategy be developed to ensure that the solution (DHT-enhanced training or rehabilitation concept) to be (further) developed will be made available to the intended target populations and remain available after the project's completion. This strategy should address its long-term availability, which could

be provision and maintenance of open-access to software, developing a business plan and outlining a plan to commercialize the solution, or transferring intellectual property rights to an existing company. Depending on this, mechanisms for ongoing maintenance and support, scalability to reach a broader audience or adapt to different contexts, and identifying resources to support the long-term sustainability of solutions are to be worked out. This step is crucial to ensure that the project's outcomes have a lasting impact and continue to benefit the target populations in the long-term.

With these extensive preparatory contextual research steps from Phases 1 - 4, a robust conceptual foundation for the targeted co-development towards successful implementation phases of the project is laid.

2.7 Phase 5: Co-Design, Development, Testing, and Refinement

In Phase 5, this *“generative”* part of the project is initiated. This phase consists of multiple iterative cycles multiple iterative cycles of:

I. *co-designing and developing* prototypes of the DHT-enhanced training or rehabilitation concept (Phases 5.2 to 5.5),
II. short-term user experience *and safety testing* and *validation* of components of the DHT-enhanced training or rehabilitation concept (Phase 5.5), and
III. *refining* these *prototypes* by further co-design and development (Phase 5.6).

This iterative cycle is to be repeated until an *“acceptable”* solution (= all components of the DHT-enhanced training or rehabilitation concepts have been successfully validated, have a good user experience, and are safe) is achieved. The result of this phase is an *“original”* DHT-enhanced training or rehabilitation concept that enters the next phases of longitudinal evaluations.

Phase 5.1: Build a Framework for the Co-Design

To guide the conduct of the co-design workshops, a framework for the co-design should be built by integrating the findings from the previous phases (☑-item 27) and defining how inputs from different interest-holders will be weighted and prioritized during the co-design process (☑-item 28).

☑-item 27: Integrate Findings from Previous Phases

To better focus the co-design workshops on relevant aspects of the DHT-enhanced training or rehabilitation concept, the core project teams should first identify congruent aspects from previous project phases (☑-item 27a) and agree on which of the remaining aspects of the DHT-enhanced training or rehabilitation concepts require co-design procedures (☑-item 27b).

Already congruent aspects refer to (i) components of the DHT-enhanced training or rehabilitation concept that all three core viewpoints agreed on in Phase 2 and that require no further design considerations for implementation, or (ii) elements of the DHTs that already meet the design and implementation requirements defined in Phase 2 (according to ☑-item 24c) in case the project builds on such existing DHTs (as defined under ☑-item 25). Examples of congruent aspects of the components of the DHT-enhanced training or rehabilitation concept might be that the three core viewpoints all agreed that the training or rehabilitation should be conducted at home to allow flexible integration in daily life (☑-item 20a) or that the training or rehabilitation volume recommended by all three core viewpoints was approximately 150 min/week (subitem 20b).

By identifying these elements, the core project team can then elaborate a consensus that the remaining aspects of the DHT-enhanced training or rehabilitation concept require co-design to better focus future project iterations toward fulfilling the overall goal.

☑-item 28: Defining how Inputs from Different Interest-Holders will be Weighted and Prioritized during the Co-Design Process

Co-design workshops are a major challenge as various perspectives and viewpoints from different target populations (as defined under ☑-item 4) and interest-holders (as defined under ☑-item 7) need to be integrated to generate and agree on ideas about the design of the DHT-enhanced training or rehabilitation concept. To better guide these complex procedures, it is recommended to work out and define, together with the project reference committee(s), the weighting of how inputs from different interest-holders is to be prioritized when collaboratively making design decisions (☑-item 28).

For example, it might be agreed that design decisions on the graphical user interface should primarily be driven by primary and secondary end-users, whereas algorithms for individualized tailoring of the training or rehabilitation should mainly rely on scientific evidence with input from researchers and healthcare professionals. Clear definitions of each interest-holder's role and expected contributions helps streamline and guide the complex task of integrating various, potentially conflicting perspectives and viewpoints from different interest-holders – increasing the likelihood of successfully generating conceptual prototypes that fulfill all the design and implementation requirements defined in Phase 2 of the project and all involved contributors can agree on. This is especially relevant in the case of potentially incongruent findings or conflicting results between the three core viewpoints from which findings should be integrated and should help moderate and streamline the co-design workshops to increase the likelihood of successfully generating conceptual prototypes that all involved contributors can agree on and fulfill all the design and implementation requirements defined in Phase 2 of the project.

Phase 5.2: Co-Design Workshops

☑-item 29: Set the Stage to Facilitate Reflective Dialogue and Productive Workshops

Co-design workshops can be challenging as they require participants with various perspectives and viewpoints to collaboratively generate and agree on specific conceptual prototypes. Moreover, the diversity in background, education levels, and experiences of interest-holders can be expected to lead to power dynamics that influence or limit the interactions during the co-design process [53]. Therefore, a stepwise process is recommended to facilitate reflective dialog and productive workshops while mitigating potential power imbalances and thereby set the stage for a well-functioning collaborative effort.

Specifically, start the co-design workshops with a short presentation to share: the rational and overall goals of the project (☑-item 29a); preparatory findings from earlier phases of the project (☑-item 29b); and clarify the planned procedures (☑-item 29c) and expectations (☑-item 29d) with all co-design workshop participants. With respect to the latter, transparently share the weighting and prioritization of input from different interest-holders (☑-item 28) so that all participants have a clear understanding of their expected contributions while mitigating potential power imbalances. Finally, invest time in establishing mutual trust, empathy and comfort with all participants (☑-item 29e). This step is critical to foster a better understanding of the diverse needs and perspectives among interest-holders and facilitate reflective dialogue before starting the co-design or co-creation process [23, 24]. Employ techniques such as empathetic target group analysis [22] to achieve this aim.

☑-item 30: Present Existing (Parts of) Solutions

If the project chooses path 2 – to further develop existing DHT-enhanced training or rehabilitation concepts (☑-item 27) – present these solutions to all workshop participants to provide them with a clear understanding of what the project builds on and what preparatory steps led to the chosen starting point (☑-item 30). The participants should also be given the opportunity to try these existing DHTs to help them identify opportunities for improvements that can be addressed in generative co-design workshops.

☑-item 31: Generate Conceptual Prototypes

Under ☑-item 31, the *"generative"* part of co-design is performed (☑-item 31). During the co-design process, employ techniques such as 6-3-5 brainwriting [67] to ensure that all interest-holders can express their ideas without judgment [23, 24]. To facilitate collaborative idea generation, use different paper- and pencil-based, embodied, or technology-supported prototyping techniques. Simple methods may include but not limited to empathy mapping [22, 68], user journey mapping [69], bodystorming [70, 71], collaborative sketching and drawings for the rapid development of initial concepts and designs [56], or – when building on and further developing existing solutions – the cognitive walkthrough method [72]. Technology-enhanced prototyping may include the use of middleware interfacing software tools that allow for the rapid development of initial concepts and designs to the creation of prototypes of the proposed concepts (e.g., game mechanics, theme) in game engines. Such tools allow early identification of possible modifications that must be performed to facilitate user interaction (e.g., removing the need to press buttons) and are particularly useful for the development of gamified DHTs. [56]

Phase 5.3: Data Synthesis

☑-item 32: Analyze the Qualitative Data

Analyze data generated during the *"generative"* part of co-design, such as audio (and video) recordings combined with generated sketches and drawings or virtual conceptual prototypes, using qualitative research methodologies (☑-item 32), such as qualitative content analysis [73-75]. Use the frameworks defined in phase 1 to guide the interpretation of findings. For example, the *'Beyond "Just" Fun of Exergames'* framework could be useful in this stage to ensure appropriate theoretical backing of DHT designs to relevant principles in motor-cognitive learning, neurorehabilitation, and/or behavior change [19].

☑-item 33: Integrate the Findings from all Co-Design Workshops and Rank-Orders Complementary Solutions

Under ☑-item 33, present the results from each workshop to the project reference committee to collaboratively integrate the findings and rank-order complementary solutions. It is likely that there are incongruent findings or conflicting results between different workshops and complementary solutions for the same aspects of the DHT-enhanced training or rehabilitation concepts from different workshops. In this case, presents these results to the project reference committee(s) and collaboratively make consensus-guided decisions on how to integrate the findings into decisions on the design of the DHT-enhanced training or rehabilitation concepts. Ideally, consider the advice of project advisors as well.

☑-item 34: Derive a List of DHT Development Tasks to be Completed

On the basis of the derived rank-ordering (☑-item 33) and considering the resources available (defined under ☑-item 11), the core project team derives a list of DHT development tasks (☑-item 34).

Phase 5.4: Development

☑-item 35: Develop Testable Prototypes

The core project team then develops testable prototypes of the DHT-enhanced training or rehabilitation concept based on the outcomes from ☑-item 34.

Phase 5.5: Short-Term Testing and Validation

☑-item 36: Short-term User Experience and Safety Testing

The first prototypic components of the DHT-enhanced training or rehabilitation concepts are then thoroughly tested on safety and user experience (☑-item 36), and relevant components of the DHT-enhanced training or rehabilitation concept are validated (☑-item 37).

User experience refers to a complex characteristic that results from the perception of many distinct quality aspects of a product [76] that has been defined as a person's perceptions and responses resulting from the use and/or anticipated use of a product, system, or service [77]. User experience involves a range of different subconstructs, which may be of varying relevance between different contexts and products under evaluation [78-80]. Consequently, the is a large number of different questionnaires available that focus on different aspects of user experience.

One prominently evaluated subconstruct in the field of digital health technologies is usability [80]. Usability can be described as a general quality of the context-specific appropriateness to a purpose of any particular artefact (i.e., a tool or system) considering (i) effectiveness (the ability of users to complete tasks using the system, and the quality of the output of those tasks), (ii) efficiency (the level of resource consumed in performing tasks), and (iii) satisfaction (users' subjective reactions to using the system) [27]. Prominent example for usability questionnaires include the System Usability Scale (SUS) [27] with its multi-language toolkit [81], or its revised version with adjective ratings for better interpretability of the outcomes [28], that mainly focus on the user experience subconstructs of learnability and efficiency of use [76]. Other examples include the User Experience Questionnaire [82] that measures 6 distinct user experience aspects (attractiveness, efficiency, perspicuity, dependability, stimulation, novelty), the (Positive-Item Version) of the Visual Aesthetics of Websites Inventory [83, 84] that measures the visual appeal of a product by 4 subscales (simplicity, diversity, colorfulness, craftsmanship), or the IsoMetrics usability inventory [85] that measures quality aspects described in the ISO 9241 - 210 (i.e, Suitability for the task, Self descriptiveness, Controllability, Conformity with user expectations, Error tolerance, Suitability for individualization, and Suitability for learning).

Given that these questionnaires only assess a selection of subconstruct of user experience, there might be situation where none of the existing questionnaires contains all scales necessary to answer a specific research question and researchers may be tempted to combined different scales. In an attempt to address the dilemma,

Schrepp and Thomaschewski introduced and validated a modular framework that allows users to the creation of user experience questionnaires that fit perfectly to a given research question. The framework contains several scales that measure different user experience aspects, including attractiveness, efficiency, perspicuity, dependability, stimulation, novelty, trust, aesthetics, usefulness, intuitive use, value, trustworthiness of content, quality of content, and haptics. The modular framework allows to construct tailored user experience questionnaires that are perfectly aligned with the specific research question and context of use in a uniform format. While the shortcoming of such a modular questionnaire is the lack of a benchmark, application of this modular user experience is mainly recommended when comparing multiple measurements of the same product over time [76], which perfectly matched the iterative process proposed by the *Co-Develop-IT guideline*.

In any case, user experience evaluations should not be reduced to a specific (selection of) questionnaire(s), but should rather combine the advantages of both quantitative and qualitative research methodologies. Step-by-step instructions and best practices for conducting such a mixed methods approach for user experience evaluations are provided by Campbell (2024) [5], which are recommended to follow. In this regard, it is also recommended to collect specific qualitative data on suggestions on improvements of components of the DHT-enhanced training or rehabilitation concept to optimize user experience and maximize perceived usefulness via semi-structured interviews with individuals with primary and secondary end-users to inform iterative further developments and refinements.

However, the authors also note that this framework does not cover all potential aspects of user experience. Therefore, their scale may be complemented with other scales for some products and new use cases and product types [76]. On example might include gamified DHTs, such as exergames, for which the Exergame Enjoyment Questionnaire [86], the Game Engagement Questionnaire [87], or the Immersive Experience Questionnaire Game Experience Questionnaire [88] provide more specific scales.

Safety of components of the DHT-enhanced training or rehabilitation concept and interaction with the DHTs should be assessed in relation to the characteristics and capabilities of the targeted primary end-users. Therefore, it is recommended to consider the perspectives of both primary and secondary end-users. Particularly, having trained professionals closely observing play-test sessions to assess the safety (e.g., risk of falls or injuries) along with conducting qualitative research with primary and secondary end-users to assess perceived safety and collect suggestions for improvements of the safety of the DHT-enhanced training or rehabilitation concept is suggested to provide a comprehensive view of the safety of components.

☑-item 37: Validate Relevant Components of DHT-enhanced Training or Rehabilitation Concept

It is recommended to thoroughly validate the relevant components of the DHT-enhanced training or rehabilitation concept according to the requirements defined under ☑-item 15b. This may include but is not limited to software algorithms for player movement detection (e.g., [89]), (game) performance metrics (e.g., [90-94]), (gamified) assessments (e.g., [95]), or mechanisms for individualized tailoring (e.g., [90]).

An example of previous research on these topics is that of Guggenberger et al. (2021), who tested the instrumental validity of the motion detection accuracy of a smartphone-based training game in 30 healthy participants compared with a 3D motion analysis system as the gold standard, confirming that the accuracy of the training game's motion

detection is sufficient for use in exergames and to quantify progress in patients' performance [89]. Other examples include (i) the evaluation of construct validity, test-retest reliability, sensitivity to changes in an evaluation module with its computerized, game-based assessment strategy to measure training or rehabilitation effects on motor-cognitive functions [95]; (ii) the validation of exergame performance metrics in relation to clinical neuropsychological reference assessments [90-94]; and (iii) the validation of exergame performance metrics for their use to inform personalized adjustments of the difficulty and complexity of gamified DHTs [90].

☑-item 38: Check Whether the Quality Criteria Defined Under 15c Have Been Reached

At this project checkpoint, for each of the defined project subgoals (as defined under ☑-item 15a), it is to be checked and verified whether the quality criteria (as defined under ☑-item 15c) for progressing to the next step are achieved, or whether additional iterations or even a regression to a previous phase are needed to steer the project towards achieving an *"acceptable"* rating in each of the quality criteria in next iterations. Accordingly, the project is to be progressed according to the progression criteria defined under ☑-item 15d. Adhering to these criteria defined in Phase 0 of the project ensures transparency in the interpretation of the data to align project progression with the collaboratively agreed-upon goals.

Phase 5.6: Iterative Refinements

☑-item 39: Share the Outcomes from Short-Term Testing with all Co-Design Workshop Participants

Before moving forward with the productive parts of additional project iterations to refine existing prototypes until an *"acceptable"* solution is achieved, it is critical to transparently share the results from previous iterations to make sure all co-design participants are informed about which aspects of the DHT-enhanced training or rehabilitation concept require attention during refinements. Furthermore, transparently sharing identified problems or barriers for goal achievement with the current prototypes might help spark creativity of participants to refine these solutions to better address the requirements of primary and secondary end-users.

☑-item 40: Additional Project Iterations until Goal Achievement

The procedures described under ☑-items 31 to 39 are iteratively repeated according to the specific subgoals and progression criteria defined under ☑-item 15a and 15d until an acceptable solution (= all components of the DHT-enhanced training or rehabilitation concept have been successfully validated and have acceptable user experience and good safety levels, as defined under ☑-item 15) is achieved. In this project phase, it is critical to adhere to the progression criteria defined under ☑-item 15d to allow the necessary iterations for achievement of subgoals (as defined under ☑-item 15a) and their quality criteria (as defined under ☑-item 15c) and the project should only be progressed to Phase 6 once all quality criteria are met that allow progressing to the next step as defined under ☑-item 15d. Once this has been achieved, the project moves forward with longitudinal evaluations.

2.8 Phase 6: Feasibility and Longitudinal User Experience Evaluation

For the phases introduced thus far, the checklist provides best practice recommendations that are to a large extent novel, expand existing guidelines or frameworks, and are specific to DHT-enhanced training or rehabilitation. Subsequent phases 6 and 7+, covering longitudinal evaluations and implementation, are more generally applicable and well covered by established guidelines. Therefore, we provide (i) recommendations which established procedures to follow and (ii) which additional elements to consider to make these guidelines better tailored to DHT-enhanced training or rehabilitation.

In phase 6, test the feasibility of the full DHT-enhanced training or rehabilitation concept and the study procedures for subsequent full-scale trials, along with a more in-depth investigation of the user experience and safety of the full DHT-enhanced training or rehabilitation concept (☑-item 41 and 42). This phase is again iterative, meaning that it is repeated according to prespecified progression criteria until a solution with acceptable feasibility and user experience among primary and secondary end-users is achieved (☑-item 43).

Evaluate *feasibility* of the study procedures and the DHT-enhanced training or rehabilitation along with its *user experience* on the basis of the conceptual framework of Eldridge et al. (2016) [2], following the terminology and recommendations of the Medical Research Council guidance [3], and considering mixed-method evaluations of both feasibility and user experience following O'Cathain et al. (2015) [4] and Campbell (2024) [5]. Like in Phase 5, collect complementary qualitative data that provides broader contextual information and obtain data on specific suggestions for improving the feasibility and user experience from both primary and secondary end-users. Extend the above-mentioned guidelines by the following items to accurately reflect the context of evaluations of individually tailored DHT-enhanced training or rehabilitation concepts (☑-item 42):

First, together with the project reference committee, work out and agree on a *traffic light system-based assessment framework* with predetermined progression criteria (☑-item 42a), as elaborating in more detail under ☑-item 15 which laid the foundation for this step. To ensure transparency in the interpretation of the results on feasibility and user experience, we recommend the traffic light system-based assessment framework be published (as part of a study protocol) and pre-registered (e.g., on the Open Science Framework) before starting data collection.

Second, in agreement with the MIDE-framework [56], systematically evaluate and report on *technology performance* (☑-item 42b; e.g., downtime, type and frequency of occurrence of technical problems, effectiveness of supportive strategies for end-users, etc.) to provide a robust basis for further iterative DHT refinements.

2.9 Phases 7⁺: Efficacy, Effectiveness, Implementation, and Economic Evaluation

Finally, perform efficacy, effectiveness, implementation, and economic evaluations of the resulting DHT-enhanced training or rehabilitation concept, depending on the context of the project (☑-item 44). In any context, we advocate for moving beyond efficacy evaluations to address the lingering evidence-to-practice gap – which is in line with the recommendations by Oluwatoyosi et al. (2020) [96]. This could be achieved by effectiveness and implementation

studies following efficacy trials, or with hybrid effectiveness-implementation designs [97-99]. Moreover, assess the DHT-enhanced training or rehabilitation concept's value (risk-benefit profile, cost-effectiveness) for healthcare systems to provide data that allow informed decisions on the uptake of such approaches in future clinical practice guidelines.

For these evaluations, we recommend following the UK Medical Research Council guidance [3] for efficacy and effectiveness terminology and evaluations, the PRACTical planning for Implementation and Scale-up guide [6] for implementation evaluations, and the World Health Organization guide [7] for cost-effectiveness analyses (☑-item 44). Like in phase 6, expand the above-mentioned guidelines to accurately reflect the context of evaluations of individually tailored DHT-enhanced training or rehabilitation concepts (☑-item 45) as follows.

First, while RCTs are considered the gold standard for evaluating efficacy and effectiveness [8], consider *alternative study designs* that may better accommodate the individualized and adaptive nature of tailored DHT-enhanced training or rehabilitation [9] (☑-item 45a). Nahum-Shani et al. (2022) [100] provide a pragmatic framework for selecting such alternative designs that are particularly relevant when interventions are multicomponent or tailored and when there are research questions regarding the timing, sequencing, or responsiveness of different intervention components. For example, a factorial design is a type of randomized trial in which two or more independent variables are manipulated simultaneously, allowing researchers to assess both the main effects of each factor and potential interactions between them. This makes it an efficient approach for studying multiple intervention components within a single trial. Furthermore, the Sequential Multiple Assignment Randomized Trial design includes multiple randomizations, enabling participants to be re-randomized based on their response or adherence to the initial treatment, which is particularly useful when behaviors or conditions evolve slowly over time. In contrast, Micro-Randomized Trials involve frequent, often daily, randomizations to assess the short-term effects of brief interventions – such as prompts or notifications – on rapidly changing outcomes. Micro-Randomized Trials are valuable for real-time optimization and adaptation of DHT-enhanced training or rehabilitation. Despite the promise of these alternative approaches in addressing the need for tailoring and adaptability in digital health, they remain underutilized in health promotion and disease prevention interventions [100].

Second, continue with *mixed methods approach* also at this stage of a project. Specifically, collect complementary participant-reported outcomes and qualitative data on perceived efficacy/effectiveness and acceptance of the implementation of primary and secondary end-users, as well as suggestions on how these could be (further) improved (☑-item 45b). These evaluations can provide insights not ascertainable through physiological, laboratory, clinician-reported, observer-reported, or performance outcomes alone. These evaluations can thereby bring additional value and optimize the impact on clinical practice and health policy. [101]

Supplementary File 3 - First application example:

Project *'Park-MOVE'* - Co-design, development and evaluation of an individually tailored training concept for individuals with Parkinson's disease

3.1 Overview

The Park-MOVE project ultimately aims to reduce the disease burden in individuals with Parkinson's disease (PD), by iteratively contextualizing, co-developing, testing, refining, evaluating, and implementing a novel exergame-based training concept for the secondary to tertiary prevention of PD. The focus of the project is to develop a precision rehabilitation approach that is (i) personalized based on multimodal data (i.e., motor, clinical, neuropsychological and neuroimaging) to inform decision making on the type, content, and characteristics of the training and (ii) individually tailored and progressed according to real-time physiological and exercise performance data. The application example will focus on phases 0 – 2 since the project currently is in phase 2 of implementing the *Co-Develop-IT guideline* with phases 0 and 1 shortly to be completed.

3.2 Phase 0: Definition of Project Framework

Phase 0.1: Define Overall Context and Goals of the Project:

Problem: PD is the fastest growing neurological disorder and therefore urgently requires evidence-based, well-accepted, and effective measures to mitigate this key challenge for aging societies and healthcare systems. [102, 103] (☑-item 1 - problem). To address this problem, the following key elements of the project have been defined:

Target Populations (☑-item 4):

Individual Level:
- Primary End-Users: Individuals with PD
- Secondary End-Users: Healthcare professionals plus relatives and carers of individuals with PD

Provider Level:
- Primary Provider: Healthcare professionals (i.e., physical and occupational therapists)
- Secondary Provider: Swedish healthcare institutions that provide outpatient rehabilitation

Remaining Levels:
- Organizations: Regional public bodies responsible for outpatient rehabilitation

Type of Intervention (☑-item 5): Exergame-based multidomain (physical, motor, and/or cognitive) training

Mode of Delivery (☑-item 3): Hybrid; via combination of:
- ➩ home training with
- ➩ clinical-based supervised training

Focus of the Project: Individually tailoring the training in terms of
- ➩ personalization of the components and start level of the exercises, and
- ➩ individualized progression of the exercises

Outcome Domain(s) (☑-item 6): General disease and symptom progression; core outcomes centered around physical, motor, and cognitive functioning and qualify of life

In the following, we briefly outline the rationale for these choices as well as the research and knowledge gaps to be addressed.

Intervention type: PD affects the central, peripheral, and enteric nervous systems and is characterized by both motor and non-motor symptoms, with gait and cognitive impairments being common manifestations [104-110]. Physical exercise is one of the most promising disease-modifying interventions and is highly recommended for the treatment of PD [111]. However, multidisciplinary and collaborative approaches are recommended when developing individually tailored and technology-enhanced training approaches that incorporate different types of exercise to better address motor and/or cognitive deficits [111]. Therefore, effective interventions for prevention of PD should target physical, motor, and/or cognitive domains. (☑-item 5)

Exergame-based training: Exergames can offer different exercise modalities with the same equipment, i.e. cardiorespiratory, balance, and cognitive exercises and can be designed to enhance the ecological validity of exercises and offer unique advantages for tailoring interventions beyond conventional approaches [19]. Exergames also allow remote supervision and can be used for home-based or hybrid therapy [112]. (☑-item 2)

Intended context of use: Hybrid intervention delivery to combine the advantages of home-based and clinic-based training is recommended to support transferability to clinical practice. [18, 112] (☑-item 3).

Research gap: The current state of evidence suggests that exergame-based training was similarly effective [113] or even slightly better [114-116] compared to conventional exercises to improve physical performance in individuals with PD. However, no conclusion can be drawn on cognitive functioning and quality of life [113]. There is also a large heterogeneity of previously evaluated concepts [113-116], whereas the field of purpose-developed and individually tailored exergame-based training concepts for individuals with PD is still in its infancy, particularly for implementing hybrid intervention delivery (☑-item 1 – knowledge gap).

Relevant target populations: Experts from all related fields along with the primary and secondary end-users as well as organization and experts involved in training implementation and delivery must be included throughout the project to support rigorous co-development and implementation of a high-quality quality intervention (☑-item 4).

Phase 0.2: Define Requirements on Interest-Holder Involvement, Resources, and Regulatory Environment for the Project:

Given the larger project started prior to the establishment of *Co-Develop-IT guideline*, most of the contextual requirements (☑-item 7), required resources (☑-item 8), and regulatory requirements (☑-item 9) were critically assessed before a project consortium was established. Grants have been secured covering the first phases in terms of staff, expertise, facilities, and data. The core project team further decided to rely on exergame technology to enhance the delivery of the intervention, for which additional resources are required, as well as partners on technology development and provision. This could be either industry or academic partners that provide software and hardware developments skills for serious exergame development. They should fulfill the following requirements presented in Table 2.

Table 2: Requirement on interest holder involvement for the *'Park-MOVE'* project.

Requirement	Description
1. Expertise: Criterion 1 (☑-item 8d)	Software development skills and experience in exergame development.
2. Expertise: Criterion 2 (☑-item 8d)	Skills/experience in different types of hardware (e.g., camera, sensor, and mixed reality-based systems) to ensure flexibility in the technology selection (Phase 4), Ideally, driven by academic collaborators.
3. Time and Staff (☑-item 8a and 8b)	One software engineer in Phases 5 and 6 to ensure sufficient engineering time for the project along with one technology expert for the project core group.
4. Funding (☑-item 8c)	Secure funding or sponsorship for requirement 3.

Phase 0.3: Establish a Project Consortium and Define each Interest-holders' Roles Throughout the Project:

To fulfill the needs for a project consortium (☑-items 7 - 9), a multidisciplinary group of researchers has been formed. The research team includes members of multiple research areas as well as an established network of national and international collaborators. Given that many of these researchers have dual appointments with the Swedish healthcare system, we profit from a strong network with healthcare provider institutions and healthcare professionals that will help in the establishment of a project consortium. Based on this, we have established the following:

Core project team (☑-item 10a): The Park-MOVE **research group** consists of team-members with career stages ranging from Professor to PhD student. Roles within the team have been distributed depending on competence and experience, e.g. principal investigator, project coordinator, and relevant expertise related to the project (i.e. physiotherapy, psychology, eHealth and exergame intervention development, and motor-cognitive assessment). In addition, numerous expert collaborators are available for neuropsychology, statistics, neuroimaging, and biomarker assessment for PD. Our **technology partner(s)** will provide the necessary expertise to drive technology development in this project as defined as requirements under item 8. Start-up funding for their initial contributions to the project has been secured.

Verification that the core project team meets all contextual (as defined under ☑-item 7) and resource-dependent (as defined under ☑-item 8) requirements is contingent on securing funding for the contributions of the technology developers as well as project phases 5 and onwards (☑-item 12).

Reference Committee (☑-item 10b):

Members of the reference committee (recruitment is ongoing) will include a minimum of:

- ⇨ 2 PD representatives (☑-item 7f),
- ⇨ 1 - 2 technology representatives (☑-item 7c), and
- ⇨ 3 representatives of the healthcare provider institution(s) (☑-item 7b);
  - 1 on organizational level (☑-item 4e), and
  - 2 on individual level (☑-item 4c).

Project Advisors (☑-item 10c):

Legal, compliance, and innovation offices, as well as other support units, will be involved in establishing to collaboration agreements and support efforts toward getting the final exergame systems and training concept patented, certified as medical device and/or commercialized.

Phase 0.4: Collaboratively Agree on Specific Subgoals and Progression Criteria as well as a Preliminary Time Plan for the Project

The establishments of project checkpoints with the related traffic light system-based assessment framework (☑-item 15) is contingent upon the consensus discussion process between the core project team and the project reference committee, which is planned for fall 2025.

3.3 Phase 1: Identification of Guidelines, Principles, Frameworks, and Theories

Examples of existing guidelines or evidence-based recommendations for the overall goals of the project and principles, frameworks, and theories that the project will be built on are:

Existing guidelines and evidence-based recommendations:

- ✓ Global consensus on optimal exercise recommendations for enhancing longevity in older adults [16],
- ✓ European Physiotherapy guidelines for PD [33, 117]. For specific local context, the Swedish physiotherapy guidelines for PD [118] are considered.
- ✓ Exercise Guidelines for Gait Function in Parkinson's Disease [119],
- ✓ Exercise as medicine in Parkinson's disease [111]
- ✓ Proposed categorization and gold standards for exergame research to impact cognition [36]

Principles, Frameworks, and Theories:

- ✓ Framework for understanding balance dysfunction in Parkinson's disease [120]
- ✓ Aspects from behavior-change frameworks applicable to physical training [41, 48]
- ✓ Beyond *"Just"* Fun of Exergames Framework [19]

3.4 Phase 2: Determine Design and Implementation Requirements

We are working on a narrative synthesis of the literature together with two qualitative studies – one study with individuals with PD (primary end-users) and one focus group study with the remaining interest holders.

Focus Group Study with Intended Secondary End-Users

Four focus groups (8 sessions) have been conducted so far. We included 22 participants, of which six were PD relatives (partners) and 16 were healthcare professionals (one neurologist, six physiotherapists, two nurses, five occupational therapists, one neuropsychologist, and one speech & language pathologist) were recruited across four different hospitals/rehab clinics in Stockholm (study protocol: https://doi.org/10.17605/OSF.IO/KX4PQ). Data analysis will start in the fall 2025 applying qualitative content analysis according to published guidelines [73, 74, 121] with mainly an inductive approach. Planning and reporting of results will follow the consolidated criteria for reporting qualitative research [122]. Data collection is expected to be completed in fall 2025.

Semi-structured Interview Study with Individuals with PD

For the individual interview study with people with PD, similar data collection and analyses methods will be used as in the focus group study, but adapted to semi-structured, individual interviews [123]. We aim to include 15 - 20 individuals with PD in the study based on recommendations from a systematic review [124]. Data collection is expected to be completed in 2025.

Literature Reviews

To define requirements for core components of the DHT-enhanced training concept, we completed three reviews. The **first review**, mapped the design, content, delivery, tailoring, and reporting of different balance training types for individuals with PD. We found multi-modal and sensory-motor integrated interventions to be superior in the number of balance components that were challenged in the training, whereas exergame-based interventions were superior in implementing strategies to monitor and tailor exercise intensity [125]. The **second review**, provided evidence on dose-response relationships of specific exercise and training variables of exergame-based training on cognitive functioning in middle-aged to older adults. Our meta-analyses showed that the effectiveness of exergame-based training was moderated by several training components that have in common that they enhance the ecological validity of the training. The results favored, for example, exercises with stepping movements in a standing position, a combination of training at home and on-site training, and group training [18].

The **third review**, shed light on serious exergame features that enhance the ecological validity of exercises and tailoring interventions beyond conventional approaches. To this end, we reviewed the roles and mechanisms of specific exergame features in supporting adherence to relevant behavior change, neurorehabilitation, and exercise principles. Our results highlighted that numerous *"serious"* exergame features offer unique opportunities beyond conventional approaches to promote behavior change towards sustained physical and/or motor-cognitive training, which are somewhat neglected in contemporary practice. Examples of such features include biofeedback, gamified assessment, mini-challenges, streak counters, rhythmic multisensory cueing, monitoring and providing multisensory feedback and rewards on exercise adherence or accuracy of movements, and manipulation of task predictability [19].

Given that two of these are generic reviews applicable to general middle-age to older adults (review 2) and all types of populations (review 3) we are additionally working on synthesizing the more context- and PD specific literature to extract literature-based design and implementation requirements for specific recommendations on the design of exergame-enhanced interventions in PD.

3.5 Prospective Phases

Table 3 summarizes project steps planned in the prospective phases:

Table 3: Prospective phases of the *'Park-MOVE'* project with corresponding aim and considerations of each step.

Phase	Aim	Comment/reflection
3	Technology Scoping	Based on design and implementation requirements in Phase 2.
4	Define Pathway & Sustainability Strategy	Based on information from phase 3. Our technology partners are capable of developing software and hardware, and a well-functioning prototype of an exergame based on computer vision that is at technology readiness level 4 from another project is available that may build the foundation for this project.
5	Co-Design, Development, Short-Term Testing & Validation, and Refinement	Specific aspects of the Park-MOVE that require validation will be identified, i.e. software algorithms for player movement detection, and exergame performance metrics, etc.
6	Feasibility and Longitudinal User Experience Evaluation	Conduction iterative pilot randomized controlled trials including assessment specific feasibility aspects (scientific, process), safety, user experience, training motivation, etc.
7+	Efficacy, Effectiveness, Implementation, and Economic Evaluation	Will be based on the findings from earlier phases. It will therefore be planned once data on the feasibility and potential effects of the intervention are available.

3.6 Reflection on the Use of the *Co-Develop-IT Guideline* in the *'Park-MOVE'* Project

So far, the *Co-Develop-IT guideline* has provided the project with appropriate structure and guidance of the different steps in the process. Only minor modifications have been necessary. Specifically, Phases 0 to 2 have been performed simultaneously instead of after each other, i.e., we started with data collection in Phase 2 before having reached a green light in the project checkpoint for Phase 1. This was because more time than expected was needed to discuss and reach a consensus on key elements and to decide on and recruit interest holders. We have also struggled with establishing consensus of the requirement to involve technology developer(s)/partners from Phase 0.3 onwards.

Our challenge relates to the process of locking in decisions while still being open to nuancing approaches based on new insight gained. On the one hand, we want to keep an open mind to the design and delivery requirements from the intended end-users to ensure flexibility in adopting different technological solutions when co-developing a serious exergame based on the findings from Phases 2 - 4. On the other hand, we should comply with the requirement to include a technology partner in the core project group – which is necessary to complement our expertise in these phases, which leaves us unsure whom to involve to best support this process. Therefore, it was determined that the technology partner must have skills and experience with different types of software and hardware (e.g., camera, sensor, and mixed reality-based systems) to ensure flexibility in the technology selection (Phase 4), which, however, complicates the process of finding a suitable partner.

Furthermore, concerns have been raised about the requirement of a minimum level of involvement of the technology partner in the core project team. Specifically, we have been reflecting on different options to optimally

profit from and integrate the expertise of technology partners throughout the project. In the first phases, it has been argued that the technology partner(s) might be better suited as advisors or consultants to the project since we want to be open to all solutions, and that the technology partner(s) might be more involved as an equal member(s) of the core project group in the later phases when the design and delivery requirements from the indented users have emerged. On the other hand, expertise of technology developer(s) is necessary to complement our expertise in these phases, particularly in the elaboration of design requirements for the exergame. These reflections and elaborations are ongoing, and a consensus on how and who to involve technology partners has yet to be reached.

Nevertheless, the guideline's requirement is that each phase be completed in order, as each phase builds on the findings of previous phases. Since we are working in parallel, none of the phases have been completed, and we intend to complete them in the correct order in accordance with the guidelines, to date, we have adhered fully to the *Co-Develop-IT guideline*. Nevertheless, some project members have raised concerns that in our context the guideline might not be fully flexible enough in Phase 0. This highlights the need to further test the *Co-Develop-IT guideline* in practice in different co-development projects and contexts to further verify its practical feasibility.

We are optimistic that our extensive contextual preparatory research will pave the way for the successful establishment of our exergame solutions. Specifically, our preliminary data provide a range of insights from the focus group participants that otherwise may have been overlooked or neglected – particularly in terms of requirements for the design and delivery of the intervention to optimally integrate a solution to the existing organizational and personal preferences of healthcare professionals. Considering these elements before starting the co-development process will very likely make a difference by establishing a solution that is more finely tuned to local needs and requirements – thereby facilitating future uptake and scalability in clinical practice.

Supplementary File 4 - Second Application Example:

Project *‘Better Together’*

4.1 Previous projects leading to *'Better Together'*

This application example of the *Co-Develop-IT guideline*, the currently ongoing *‘Better Together’* project, does not start from scratch. The *‘Better Together’* project starts after years of co-creation of an augmented-reality (AR) rehabilitation platform for people with Parkinson’s disease between the Vrije Universiteit (VU) Amsterdam and Strolll Limited (Ltd.), the manufacturer of the AR software [126-128]. Strolll Ltd.'s gamified AR exercise program for supervised in-clinic use and remotely prescribed independent use at home aims to enhance exercise accessibility and engagement to promote long-term adherence to exercise, an essential but challenging part of Parkinson's disease (self-)management [129] (**☑-item 2 – problem**). The gamified exercises are designed with a focus on gait, balance, and fall risk. Our core team (Table 4, **☑-item 10 – core team**) consists of researchers from the department of Human Movement Sciences at VU Amsterdam and clinicians (secondary end-users, who also represent the primary end-users through monthly community visits; **☑-item 4 - target populations**), software developers and designers, and legal and commercial experts from Strolll Ltd..

As part of phase 6 of the *Co-Develop-IT guideline*, our core team’s researchers evaluated feasibility and potential effects [127, 128]. During both clinical trial 1 [127] and 2 [128], people with Parkinson’s disease trained with the AR glasses for 6 weeks independently at home. During clinical trial 2, the exercise program was remotely managed by clinicians in fifteen Dutch primary care practices. Furthermore, our core team’s researchers evaluated the acceptability of the exercise program qualitatively with both primary and secondary end users [130]. Insights from these evaluations revealed, among other findings, that both individuals with Parkinson’s disease and clinicians perceived a lack of cognitive engagement within the exercises. This prompted a critical reflection on our earlier co-design decisions, particularly the emphasis placed on motor function training. Through a series of explorational discussions with cognitive science experts from Aachen University and ETH Zurich, our core team acknowledged the need to revise our project framework to better align with the identified user needs and to incorporate expertise from the cognitive domain to move towards a more holistic, multi-domain rehabilitation approach. That is, motor and cognitive training are *‘Better Together’* as the simultaneous training of motor and cognitive functions can produce synergistic effects that exceed the benefits of training these domains in isolation (i.e., 1 + 1 = 3) [19, 131-139] (**☑-item 2 – knowledge gap**).

In this application example, we will outline our *‘Better Together’* contextual research steps for phases 0 to 2. Given the established public–private partnership of the core team and the extensive development, feasibility, and usability testing of the AR software, the choice of technology in Phase 3 and the decision in Phase 4 to build on existing solutions (rather than starting from scratch) are already largely defined. Nevertheless, in Phase 3 the core team will critically review available motor-cognitive training solutions – both technology-enhanced and non-technology-enhanced – based on the requirements identified in Phase 2. In Phase 4, we will determine to what extent cognitive elements can be integrated into existing motor exercises or whether entirely new exercises are

required. Sustainability also remains a central focus. For instance, we addressed long-term hardware availability by ensuring the software is device agnostic and runs on multiple platforms (Microsoft HoloLens 2 and Magic Leap 2), which proved valuable given that HoloLens 2 development is ending and multiple other tech companies are expected to soon launch AR glasses meeting the a-priori defined technology requirements. We further support sustainability through a Strolll Ltd. help-desk to ensure a consistent user experience, and by embedding the intervention in the healthcare system through effectiveness-implementation research [98] - testing the intervention in real-world care settings at an early stage.

4.2 Phase 0: Definition of the project framework

To define the overall context and goals of the project, we set up an initial *'Better Together'* reference committee of researchers and clinicians in the cognitive and motor-cognitive field (Table 4 - **☑-item 10 the reference committee**) and verified the knowledge gap in science and clinical practice regarding the synergistic effects of simultaneous motor-cognitive training (**☑-item 2 – knowledge gap**).

Table 4. The core team and *'Better Together'* reference committee..

The Core Team	Two researchers from the department of Human Movement Science at the Vrije Universiteit Amsterdam (VU) and the department of Nutrition and Movement Sciences at Maastricht University. The principal investigator is also the scientific advisor of Strolll Limited for one day a week.<br><br>Clinicians (secondary end-users, who also represent the primary end-users through monthly community visits; **☑-item 4 – target populations**), software developers and designers, and legal and commercial experts from Strolll Limited.
The Better Together Reference Committee	Researchers and clinicians affiliated with ETH Zurich (Department of Health Sciences and Technology) and OST – Eastern Switzerland University of Applied Sciences (Department of Physiotherapy), Karolinska Institutet (Department of Neurobiology, Care Sciences and Society), RWTH Aachen University (Department of Neurology), Ruhr University Bochum (Neuropsychological Therapy Centre) and University San Raffaele (Division of Neuroscience).

In line with this knowledge gap, the *'Better Together'* project team identified an implementation gap, that is, a lack of collaboration between these two fields. The *'Better Together'* project, has three goals (**☑-item 15a - project-specific subgoals**). The first goal is to outline the state-of-the-art in clinical practice to improve or maintain motor and cognitive functioning. The second goal is to elaborate on *why* bridging the gap between these two fields will advance research, development and clinical practice to improve or maintain motor and cognitive functioning in in-clinic, home or combined settings (**☑-item 3 - intended context of use**) and move towards providing multidomain (simultaneous physical/motor and cognitive) training (**☑-item 5 – intervention type**). The third goal is the end product of phase 0 and 1 (the synthesis of the evidence); the publishing of a so-called *"call-to-action"* paper (**☑-item 15b - achievement of the project-specific subgoals**), which includes an elaboration of the first and second goal as well as practical recommendations on *how* to bridge the gap between cognitive and motor fields (the *"call-to-action"*). Ultimately, the core team's overarching goal is to co-develop (with the *'Better Together'* reference committee), test, refine, evaluate and implement motor-cognitive exercises as part of Strolll Ltd.'s AR software (**☑-item 3 – the overarching goal**). The core team and the *'Better Together'* reference committee broadened their scope from primary end-users with Parkinson's disease to the aging (whether pathological or non-pathological)

population, following the WHO model of intrinsic capacity that encourages the maintenance of functional ability rather than the treatment of disease [140] (☑**-item 4 – target populations**). Secondary end-users include clinicians with cognitive and/or motor expertise. Primary and secondary providers include clinicians with cognitive and/or motor expertise in primary or secondary healthcare facilities, respectively. Finally, the core team and the *'Better Together'* reference committee will discuss **outcome domains** of the multidomain training (e.g., a combined motor-cognitive outcome measure) resulting from the synthesis of the evidence (phase 1) and consensus discussions (☑**-item 6 – outcome domain**).

The core team and the *'Better Together'* reference committee critically reflected on the representative group of interest-holders and the defined required resources and the regulatory environment under which the DHT-enhanced training or rehabilitation concept should be co-developed and tested. The **required multidisciplinary interest-holders** to successfully complete the *'Better Together'* project were identified (☑**-item 7**) and **required resources** were specified (☑**-item 8**). The project includes the involvement of:

- researchers and clinicians with expertise in cognitive and/or motor (rehabilitation) fields, all investing their time and expertise (See Table 4 - ☑**-item 7a and 8a and d**);
- primary and secondary healthcare facilities in the UK and The Netherlands to co-design, evaluate, test and refine motor-cognitive AR exercises, in collaboration with the core team and the *'Better Together'* reference committee, all investing their time, expertise and facilities **(☑-item 7b, 8a, d, f**);
- designers and developers from Strolll Ltd. for the development, testing and refinement of the motor-cognitive AR exercises and technical support and maintenance, as well as for supplying the (already established) technological AR platform infrastructure (AR glasses, routers, data storage, cybersecurity, et cetera), investing time, staff, expertise, data and facilities **(☑-item 7c and d, 8a, b, d, e, f)**;
- legal officers from Strolll Ltd. to address regulatory aspects and actions related to (identifying the need for) updating Strolll Ltd.'s medical device certification (CE, UKCA, FDA) for incorporating motor-cognitive AR exercises in a safe and effective manner (☑**-item 7e, 8a, b, d, f**).
- No additional interest-holders were identified at this stage (☑**-item 7f)**.

Funding to construct the call-to-action paper was primarily in kind. To strengthen collaboration and learn from the *'Better Together'* reference committee members, funding for research visits was provided by a PhD trajectory travel grant (☑**-item 8c - funding).** The time plan included biweekly meetings from February to September 2025 – with the goal to get the paper published in the months after (☑**-item 16 – preliminary project time plan)**.

Key regulatory requirements to be followed in the *'Better Together'* project (☑**-item 9 – regulatory requirements**) build on an existing collaboration agreement between the VU and Strolll Ltd. (i.e., since 2021 upon receipt of the joint research and development grant and transfer of intellectual property; ☑**-item 9c - collaborations**). From a medical device regulatory perspective, Strolll AR neurorehabilitation platform is software as a medical device certified under CE, UK-CA and FDA regulations (**item 9d – medical device regulations**), and requires adjustments before clinical and performance claims regarding cognitive and/or motor benefits due to cognitive-motor AR exercises can be made, as the intended use (type of training) and intended users (population) will change. The required clinical evaluation to assess the risk-benefit of combined cognitive-motor training in AR is ongoing in parallel to the *'Better Together'* project (☑**-item 9 – regulatory environment)**.

4.3 Phase 1: Identification of Guidelines, Principles, Frameworks, and Theories

The *'Better Together'* group identified guidelines, principles, frameworks and theories from both the motor and cognitive fields to outline the state-of-the-art in clinical practice to improve or maintain motor and cognitive functioning and to elaborate on why bridging the gap between these two disciplines will advance research, development and clinical practice to improve or maintain motor and cognitive functioning in in-clinic, home or combined settings and move towards providing multidomain (physical, motor, and/or cognitive) training.

Examples are:

- Guided plasticity facilitation framework [42]
- WHO model of intrinsic capacity [140]
- Motor learning principles [40]
- Beyond *"Just"* Fun of Exergame Framework [19]
- Somato-Cognitive Action Network [141]
- Motor-cognition [142]
- Action-observation and/or motor imagery [143]

4.4 Phase 2: Determine Design and Implementation Requirements

In response to the identified implementation gap in clinical practice, the Better Together reference committee recognized the importance of not only synthesizing and communicating the scientific rationale for integrating motor and cognitive domains (the *why*), but also to provide insights on *how* to bridge motor and cognitive domains (i.e., the call-to-action). This extends beyond clinical settings and includes implications for research and industry. These recommendations will be the building blocks for the design and implementation requirements for Strolll Ltd.'s AR motor-cognitive exercises. The synthesis of the evidence will be combined with the insights from our primary and secondary end-users collected through qualitative research [130] and continuous Parkinson UK community visits by Strolll Ltd.'s clinical and design experts to collect feedback on user experience and usability.

4.5 Reflection on the Use of the *Co-Develop-IT Guideline* in the *'Better Together'*

With its clear structure and point-by-point checklist, the *Co-Develop-IT guideline* enabled the project core team to critically reflect on and further professionalize our workflow, highlighting its potential value for both science and industry. Importantly, the guideline is flexible in that it allows projects to enter at any phase, to follow the *"short-track option"* items and to build upon existing products in phase 4, making it possible to revisit earlier steps without starting over entirely. The guideline's clarity and flexibility hold promise for a time-efficient process, supporting rapid yet thorough progress towards real-world impact. However, when applying the guideline within a public–private partnership, it is sometimes necessary to balance scientific rigor with industrial interests and timelines, and to find a pragmatic middle ground to move forward. For example, in our case, the development of prototype motor–cognitive exercises runs in parallel with the evidence synthesis, meaning that phases are not followed strictly sequentially as recommended. This reflects a common dynamic in public-private partnerships, where companies often operate like speedboats – navigating fast-changing technologies and markets – while academia resembles an oil tanker – slower to maneuver due to regulatory frameworks but offering thoroughness and reflection. Such differences highlight both the challenges and opportunities of jointly operationalizing co-development guidelines.

Supplementary File 5 - Key Questions to be Considered under *Co-Develop-IT-Checklist* Items 20 – 23

Table 5 provides recommended key questions to be considered under *Co-Develop-IT-Checklist* Items 20 – 23.

Table 5: Recommended Key Questions to be considered under *Co-Develop-IT Checklist* Items 20 – 23.

***Co-Develop-IT-Checklist* Item:**	**Recommended Key Questions:**
Requirements for core components of the training or rehabilitation concept;	
☑-Item 20a:<br>Training or rehabilitation location and time	At which training or rehabilitation location is the training or rehabilitation preferably taking place - in a clinical setting, in a community setting, at home, or a mixed setting by combination of some of these? Are the intended primary end-users capable of traveling to and performing training or rehabilitation in these specific settings? What is the preferred time of day for the training or rehabilitation to be optimally planned? What requirements on the training or rehabilitation concept are critical to allow the implementation of this preference (e.g., is support in traveling to the specific training or rehabilitation location or technical support in the set-up of the technology used for implementing the training or rehabilitation at home needed? What is the availability of the persons/staff supervising the training or rehabilitation in case of supervised training or rehabilitation sessions?).
☑-Item 20b:<br>Training or rehabilitation components (exercise and training variables)	Which training or rehabilitation components in terms of exercise and training variables as well as other training or rehabilitation components should preferably be chosen to promote the effectiveness of the training or rehabilitation? What are the requirements for primary and secondary end-users to be able to adhere to these training or rehabilitation components?
☑-Item 20c:<br>Personalization of the training or rehabilitation	What general principles should the personalization of training or rehabilitation follow? Which characteristics of primary end-users (e.g., cognitive, physical, psychosocial functioning) should be considered for personalization of the training or rehabilitation? Which principles and elements should be considered when developing algorithms and/or decision trees for decisions on personalization of the training or rehabilitation? Which characteristics of the training or rehabilitation should be personalized (e.g., training or rehabilitation type, specificity of the training or rehabilitation in terms of cognitive, motoric, or other training or rehabilitation elements)? What requirements should markers for determining and controlling decisions on personalization fulfill? What general principles should be followed when implementing these algorithms and/or decision trees and what principles should be followed for evaluating the success of personalization? By whom should personalization be controlled?
☑-Item 20d:<br>Individualized progression of the training or rehabilitation	What general principles should the individualized progression of training or rehabilitation follow? Which principles and elements should be considered when developing algorithms and/or decision trees for decisions on individualized progression of the training or rehabilitation? Which characteristics of the training or rehabilitation should be individually progressed (e.g., training or rehabilitation type, specificity of the training or rehabilitation in terms of cognitive, motoric, or other training or rehabilitation elements)? What requirements should markers for determining and controlling decisions on individualized progression fulfill? What general principles should be followed when

	implementing these algorithms and/or decision trees and what principles should be followed for evaluating the success of individualized progression? By whom should individualized progression be controlled?
☑-Item 20e:<br>Safety	What safety hazards (e.g., risk of falls due to the training or rehabilitation demands, risk of falls due to tripping hazards like lying around cables or similar) can be identified in the travel to, set-up, during, and in-between training or rehabilitation sessions? What are the requirements on safety measures to prevent adverse events during training or rehabilitation or in-between training or rehabilitation sessions? Is there a need for and what are the requirements to implement safety measures to manage and protect sensitive data?
☑-Item 20f:<br>Familiarization with training or rehabilitation concept and technologies	Is a guided familiarization to the training or rehabilitation concept and the technology(ies) needed for primary and secondary end-users? Which aspects require to be addressed when familiarizing primary and secondary end-users with the technology(ies) as well as the training or rehabilitation concept? How and by whom should the familiarization be preferably delivered?
☑-Item 20g:<br>Instructions	What are the requirements on the instruction of primary and secondary end-users to ensure fidelity to a technology-enhanced training or rehabilitation concept? Which aspects should the instructions cover? What is the preferred delivery mode (e.g., one-on-one or self-guided instructions delivered via verbal explanations, text instructions, video instructions, trial run instructions, or practical demonstrations in person, remotely, via the technology that will be used to implement the training or rehabilitation) of these instructions?
☑-Item 20h:<br>Feedback and rewards	What are the requirements on the type and delivery of real-time feedback to primary and secondary end-users? What are the requirements on the type and delivery of delayed (e.g., after each session) feedback to primary and secondary end-users? What are the requirements on the type and delivery of rewards to primary and secondary end-users?
☑-Item 20i:<br>Supervision and guidance	Is supervision and guidance throughout the training or rehabilitation needed? What level of supervision is needed? Who should preferably supervise the training or rehabilitation (e.g., trained professionals, relatives, a mix of those)? What is the preferred delivery mode of supervision and guidance? What should supervision and guidance entail?
**Hardware requirements for technologies to implement the training or rehabilitation concept**	
☑-Item 21a:<br>Hardware requirements to promote accessibility by target populations	What might be possible barriers in the use of the hardware of the technologies and how can these be designed and/or what should the technologies offer to overcome these barriers?
☑-Item 21b:<br>Hardware requirements to promote user experience by target populations	What measures are required and/or what should the hardware of the technologies offer to promote user experience (e.g., ease of use, acceptability, enjoyment)? What hardware features are required and/or what should the technologies offer to be usable for the target population? What hardware features are required and/or what should the technologies offer to promote user enjoyment and facilitate training or rehabilitation motivation?

☑-Item 21c:<br>Hardware requirements to mitigate or deal with potential technical problems	What measures are required and/or what should the hardware of technologies offer in the case of technical problems or difficulties in handling the technologies to support primary and secondary end-users?
**Environmental requirements at training or rehabilitation location**	
☑-item 22a: Training or Rehabilitation Equipment	What training or rehabilitation equipment is available at the intended training or rehabilitation location(s)? Who should provide eventual additional equipment? Are primary end-users or secondary end-users required to provide parts of the required equipment themselves (e.g., screens, walking aids)?
☑-item 22b: Space Requirements	How much space is required for implementing the intervention type? What requirements need to be met at the training or rehabilitation locations for the technology(ies) to be implemented? Should any constraints in the training or rehabilitation environment (e.g., safety measures such as handrails or ensuring that there are no hard objects within the potential drop-zone) be considered in the co-design phase?
☑-item 22c: Connectivity	What are the requirements on type of connectivity (e.g., Wi-Fi, Bluetooth, cables)? In case of Wi-Fi: Who should provide connectivity plans (i.e., part of usage feed of technologies, primary end-users expected to provide this themselves)? What requirements should be met to ensure connectivity in rural areas or other areas with potentially limited connectivity?
**Software requirements for technologies to implement the training or rehabilitation concept**	
☑-Item 23a:<br>Software requirements to allow implementation of defined intervention type	Which contents should the technologies be able to offer to optimally implement the defined intervention type? What are requirements on general design principles for the user interface to be followed when designing the software to promote ecological validity and specificity of the training or rehabilitation?
☑-Item 23b:<br>Software requirements to promote accessibility by target population	What might be possible barriers in the use of the software of the technologies and how can these be designed and/or what should the technologies offer to overcome these barriers?
☑-Item 23c:<br>Software requirements to promote user experience by target populations	What measures are required and/or what should the software of the technologies offer to promote user experience (e.g., ease of use, acceptability, enjoyment)? What are design requirements for the software to be usable for the target population? What are general design requirements for the user interface to promote task comprehension, ease of use, and acceptability of using the technologies? What software features are required and/or what should the technologies offer to promote user enjoyment and facilitate training or rehabilitation motivation?
☑-Item 23d:<br>Software requirements to mitigate or deal with potential technical problems	What measures are required and/or what should the software of technologies offer in the case of technical problems or difficulties in handling the technologies to support primary and secondary end-users?
☑-Item 23e:<br>Software requirements to provide instruction	What are the requirements to the software to allow implementing the requirements on providing instructions as defined under ☑-item 20g?
☑-Item 23f:<br>Software requirements to provide feedback and rewards	What are the requirements to the software to allow implementing the requirements on providing feedback and rewards as defined under ☑-item 20g? What design principles should the feedback mechanisms follow to reward and correct end-users in real-time?

	What design principles should the feedback mechanisms follow when delivering performance progressions feedback?
☑-Item 23g:<br>Software requirements to allow personalization of training or rehabilitation	What principles should be followed to allow the implementation of the requirements derived in step 3 in terms of the technology? What technological features should the technologies offer to optimally implement the requirements on personalization of the training or rehabilitation as defined under ☑-item 20c? Which design principles should be followed for optimal control, ease of use, and acceptance of the control features that allow personalization of training or rehabilitation?
☑-Item 23h:<br>Software requirements to allow individualized progression of training or rehabilitation	What principles should be followed to allow the implementation of the requirements derived in step 3 in terms of the technology? What technological features should the technologies offer to optimally implement the requirement on individualized progression of training or rehabilitation as defined under ☑-item 20d? Which design principles should be followed for optimal control, ease of use, and acceptance of the control features that allow individualized progression of training or rehabilitation?
☑-Item 23i:<br>Software requirements to allow monitoring adherence and training or rehabilitation fidelity	What features should the software offer to allow monitoring training or rehabilitation adherence (e.g., attendance adherence, duration adherence)? What features should the software offer to allow monitoring training or rehabilitation fidelity (e.g., to the prescribed training or rehabilitation components or considering movement quality)? How should the software be designed to optimize integration in clinical workflows? What are essential privacy and security measures for personal data?

References

1. Vaughn LM, Jacquez F. Participatory research methods–choice points in the research process. Journal of participatory research methods. 2020;1(1). doi: https://doi.org/10.35844/001c.13244.
2. Eldridge SM, Lancaster GA, et al. Defining Feasibility and Pilot Studies in Preparation for Randomised Controlled Trials: Development of a Conceptual Framework. PLoS One. 2016;11(3):e0150205. doi: https://doi.org/10.1371/journal.pone.0150205.
3. Skivington K, Matthews L, et al. A new framework for developing and evaluating complex interventions: update of Medical Research Council guidance. BMJ. 2021;374:n2061. doi: https://doi.org/10.1136/bmj.n2061.
4. O'Cathain A, Hoddinott P, et al. Maximising the impact of qualitative research in feasibility studies for randomised controlled trials: guidance for researchers. Pilot and Feasibility Studies. 2015;1(1):32. doi: https://doi.org/10.1186/s40814-015-0026-y.
5. Campbell JL. User Experience Research and Usability of Health Information Technology. Auerbach Publications; 2024 URL: https://doi.org/10.1201/9781003460886.
6. Koorts H, Eakin E, et al. Implementation and scale up of population physical activity interventions for clinical and community settings: the PRACTIS guide. International Journal of Behavioral Nutrition and Physical Activity. 2018;15(1):51. doi: https://doi.org/10.1186/s12966-018-0678-0.
7. Edejer TT-T, Edejer TT-T. Making choices in health: WHO guide to cost-effectiveness analysis. World Health Organization; 2003 URL: https://iris.who.int/handle/10665/42699.
8. Hariton E, Locascio JJ. Randomised controlled trials – the gold standard for effectiveness research. BJOG: An International Journal of Obstetrics & Gynaecology. 2018;125(13):1716–1716. doi: https://doi.org/10.1111/1471-0528.15199.
9. Daniels K, Quadflieg K, et al. Mobile health interventions for active aging: a systematic review and meta-analysis on the effectiveness of physical activity promotion. mHealth. 2025;11. doi: https://doi.org/10.21037/mhealth-24-41.
10. López-Gil JF, Calatayud J, et al. Trends in adherence to physical activity guidelines from 1997 to 2018 among adults with obesity: An analysis from the US National Health Interview Survey. Obesity Reviews. 2024;n/a(n/a):e13866. doi: https://doi.org/10.1111/obr.13866.
11. Bull FC, Al-Ansari SS, et al. World Health Organization 2020 guidelines on physical activity and sedentary behaviour. Br J Sports Med. 2020;54(24):1451. doi: https://doi.org/10.1136/bjsports-2020-102955.
12. Mantri S, Fullard ME, et al. Physical Activity in Early Parkinson Disease. Journal of Parkinson's disease. 2018;8:107–111. doi: https://doi.org/10.3233/JPD-171218.
13. Benka Wallén M, Franzén E, et al. Levels and Patterns of Physical Activity and Sedentary Behavior in Elderly People With Mild to Moderate Parkinson Disease. Phys Ther. 2015;95(8):1135–1141. doi: https://doi.org/10.2522/ptj.20140374.
14. Colberg SR, Sigal RJ, et al. Physical Activity/Exercise and Diabetes: A Position Statement of the American Diabetes Association. Diabetes Care. 2016;39(11):2065–2079. doi: https://doi.org/10.2337/dc16-1728.
15. Kanaley JA, Colberg SR, et al. Exercise/Physical Activity in Individuals with Type 2 Diabetes: A Consensus Statement from the American College of Sports Medicine. Medicine & Science in Sports & Exercise. 2022;54(2). doi: https://doi.org/10.1249/MSS.0000000000002800.
16. Izquierdo M, de Souto Barreto P, et al. Global consensus on optimal exercise recommendations for enhancing healthy longevity in older adults (ICFSR). The Journal of nutrition, health and aging. 2025:100401. doi: https://doi.org/10.1016/j.jnha.2024.100401.
17. Chew NWS, Ng CH, et al. The global burden of metabolic disease: Data from 2000 to 2019. Cell Metabolism. 2023;35(3):414–428.e413. doi: https://doi.org/10.1016/j.cmet.2023.02.003.
18. Manser P, Herold F, et al. Components of Effective Exergame-based Training to Improve Cognitive Functioning in Middle-Aged to Older Adults - A Systematic Review and Meta-Analysis. Ageing Research Reviews. 2024;99. doi: https://doi.org/10.1016/j.arr.2024.102385.

19. Manser P, de Bruin ED, et al. Beyond "Just" Fun: The Role of Exergames in Advancing Health Promotion and Disease Prevention. Neuroscience & Biobehavioral Reviews. 2025:106260. doi: https://doi.org/10.1016/j.neubiorev.2025.106260.

20. Herold F, Müller P, et al. Dose–Response Matters! – A Perspective on the Exercise Prescription in Exercise–Cognition Research. Frontiers in psychology. 2019;10(2338). doi: https://doi.org/10.3389/fpsyg.2019.02338.

21. Boers M, Rochereau A, et al. Classification grid and evidence matrix for evaluating digital medical devices under the European union landscape. npj Digital Medicine. 2025;8(1):304. doi: https://doi.org/10.1038/s41746-025-01697-w.

22. Cairns P, Pinker I, et al. Empathy maps in communication skills training. The Clinical Teacher. 2021;18(2):142–146. doi: https://doi.org/10.1111/tct.13270.

23. Rohner SL, Stadtmann MP, et al. Co-creation for the development and implementation of a competence centre for mental health in Eastern Switzerland: a participatory approach. BMC Psychiatry. 2025;25(1):254. doi: https://doi.org/10.1186/s12888-025-06703-9.

24. Mental Health Europe. Guidelines for Co-Creation in Mental Health. 2023.

25. Manser P, Poikonen H, et al. Feasibility, usability, and acceptance of "Brain-IT"—A newly developed exergame-based training concept for the secondary prevention of mild neurocognitive disorder: a pilot randomized controlled trial. Frontiers in Aging Neuroscience. 2023;15. doi: https://doi.org/10.3389/fnagi.2023.1163388.

26. Wallin A, Franzén E, et al. A highly challenging balance training intervention for people with multiple sclerosis: a feasibility trial. Pilot and Feasibility Studies. 2023;9(1):41. doi: https://doi.org/10.1186/s40814-023-01265-7.

27. Brooke J. SUS: A quick and dirty usability scale. Usability Eval Ind. 1995;189.

28. Bangor A, Kortum P, et al. Determining What Individual SUS Scores Mean: Adding an Adjective Rating Scale. Journal of Usability Studies. 2009.

29. Höglinger G, Bähr M, et al. Diagnosis and treatment of Parkinson´s disease (guideline of the German Society for Neurology). Neurological Research and Practice. 2024;6(1):30. doi: https://doi.org/10.1186/s42466-024-00325-4.

30. Osborne JA, Botkin R, et al. Physical Therapist Management of Parkinson Disease: A Clinical Practice Guideline From the American Physical Therapy Association. Phys Ther. 2022;102(4):pzab302. doi: https://doi.org/10.1093/ptj/pzab302.

31. Grimes D, Fitzpatrick M, et al. Canadian guideline for Parkinson disease. Canadian Medical Association Journal. 2019;191(36):E989. doi: https://doi.org/10.1503/cmaj.181504.

32. NICE - National Institute for Health and Care Excellence. Parkinson's disease in adults - NICE guideline. 2017.

33. Keus S, Munneke M, et al. European physiotherapy guideline for Parkinson's disease. The Netherlands: KNGF/ParkinsonNet. 2014.

34. Chen Y-X, Liang N, et al. Diagnosis and Treatment for Mild Cognitive Impairment: A Systematic Review of Clinical Practice Guidelines and Consensus Statements. Frontiers in Neurology. 2021;12. doi: https://doi.org/10.3389/fneur.2021.719849.

35. Veronese N, Soysal P, et al. Physical activity and exercise for the prevention and management of mild cognitive impairment and dementia: a collaborative international guideline. European geriatric medicine. 2023;14(5):925–952. doi: https://doi.org/10.1007/s41999-023-00858-y.

36. Torre MM, Temprado J-J. Effects of Exergames on Brain and Cognition in Older Adults: A Review Based on a New Categorization of Combined Training Intervention. Frontiers in Aging Neuroscience. 2022;14. doi: https://doi.org/10.3389/fnagi.2022.859715.

37. American College of Sports Medicine. ACSM's Guidelines for Exercise Testing and Prescription. Wolters Kluwer; 2025 URL: https://acsm.org/education-resources/books/guidelines-exercise-testing-prescription/.

38. Kleim Jeffrey A, Jones Theresa A. Principles of Experience-Dependent Neural Plasticity: Implications for Rehabilitation After Brain Damage. Journal of Speech, Language, and Hearing Research. 2008;51(1):S225–S239. doi: https://doi.org/10.1044/1092-4388(2008/018).

39. Kleim JA. Neural plasticity and neurorehabilitation: Teaching the new brain old tricks. Journal of Communication Disorders. 2011;44(5):521–528. doi: https://doi.org/10.1016/j.jcomdis.2011.04.006.

40. Maier M, Ballester BR, et al. Principles of Neurorehabilitation After Stroke Based on Motor Learning and Brain Plasticity Mechanisms. Frontiers in Systems Neuroscience. 2019;13. doi: https://doi.org/10.3389/fnsys.2019.00074.

41. Rhodes RE, McEwan D, et al. Theories of physical activity behaviour change: A history and synthesis of approaches. Psychology of Sport and Exercise. 2019;42:100–109. doi: https://doi.org/10.1016/j.psychsport.2018.11.010.

42. Herold F, Hamacher D, et al. Thinking While Moving or Moving While Thinking - Concepts of Motor-Cognitive Training for Cognitive Performance Enhancement. Frontiers in Aging Neuroscience. 2018;10(228). doi: https://doi.org/10.3389/fnagi.2018.00228.

43. Raichlen DA, Alexander GE. Adaptive Capacity: An Evolutionary Neuroscience Model Linking Exercise, Cognition, and Brain Health. Trends in Neurosciences. 2017;40(7):408–421. doi: https://doi.org/10.1016/j.tins.2017.05.001.

44. Cheval B, Boisgontier MP. The Theory of Effort Minimization in Physical Activity. Exercise and Sport Sciences Reviews. 2021;49(3). doi: https://doi.org/10.1249/JES.0000000000000252.

45. Gerber M, Cheval B, et al. Psycho-physiological foundations of human physical activity behavior and motivation: Theories, systems, mechanisms, evolution, and genetics. Physiological Reviews. 2025. doi: https://doi.org/10.1152/physrev.00021.2024.

46. Wulf G, Lewthwaite R. Optimizing performance through intrinsic motivation and attention for learning: The OPTIMAL theory of motor learning. Psychon Bull Rev. 2016;23(5):1382–1414. doi: https://doi.org/10.3758/s13423-015-0999-9.

47. Brand R, Ekkekakis P. Affective–Reflective Theory of physical inactivity and exercise. German Journal of Exercise and Sport Research. 2018;48(1):48–58. doi: https://doi.org/10.1007/s12662-017-0477-9.

48. El Kirat H, van Belle S, et al. Behavioral change interventions, theories, and techniques to reduce physical inactivity and sedentary behavior in the general population: a scoping review. BMC Public Health. 2024;24(1):2099. doi: https://doi.org/10.1186/s12889-024-19600-9.

49. Hamasaki T, Briand C, et al. Digital health technology adoption factors: a rapid review of systematic reviews and checklist development. Disability and Rehabilitation: Assistive Technology. 2025:1–18. doi: https://doi.org/10.1080/17483107.2025.2526175.

50. Grutters JPC, Bouttell J, et al. Defining early health technology assessment: building consensus using Delphi technique. Int J Technol Assess Health Care. 2025;41(1):e34. doi: https://doi.org/10.1017/s0266462325100123.

51. Burns PB, Rohrich RJ, et al. The Levels of Evidence and Their Role in Evidence-Based Medicine. Plastic and Reconstructive Surgery. 2011;128(1). doi: https://doi.org/10.1097/PRS.0b013e318219c171.

52. Taylor E. We Agree, Don't We? The Delphi Method for Health Environments Research. HERD: Health Environments Research & Design Journal. 2020;13(1):11–23. doi: https://doi.org/10.1177/1937586719887709.

53. Farr M. Power dynamics and collaborative mechanisms in co-production and co-design processes. Critical Social Policy. 2017;38(4):623–644. doi: https://doi.org/10.1177/0261018317747444.

54. Gavi B, Daniel H. Analysing Health Communication - Discourse Approaches. Springer Nature Link: Springer Nature; 2021 URL: https://link.springer.com/book/10.1007/978-3-030-68184-5.

55. Marta B, Maria Laura I, et al. Healthcare in the Digital Age - Perspectives for Sustainable Innovation and Assessment. Springer Nature Link: Springer Nature; 2025 URL: https://doi.org/10.1007/978-981-96-1437-0.

56. Li Y, Muñoz J, et al. Multidisciplinary Iterative Design of Exergames (MIDE): A Framework for Supporting the Design, Development, and Evaluation of Exergames for Health. International Conference on Human-Computer Interaction: Springer; 2020. p. 128–147.doi: https://doi.org/10.1007/978-3-030-50164-8_9

57. Manser P, Adcock-Omlin M, et al. Design Considerations for an Exergame-Based Training Intervention for Older Adults With Mild Neurocognitive Disorder: Qualitative Study Including Focus Groups With Experts and Health Care Professionals and Individual Semistructured In-depth Patient Interviews. JMIR Serious Games. 2023;11:e37616. doi: https://doi.org/10.2196/37616.

58. Hadjipanayi C, Banakou D, et al. Virtual reality exergames for enhancing engagement in stroke rehabilitation: A narrative review. Heliyon. 2024;10(18). doi: https://doi.org/10.1016/j.heliyon.2024.e37581.

59. Manser P, de Bruin ED. Making the Best Out of IT: Design and Development of Exergames for Older Adults With Mild Neurocognitive Disorder - A Methodological Paper. Front Aging Neurosci. 2021;13:734012. doi: https://doi.org/10.3389/fnagi.2021.734012.

60. Deci EL, Ryan RM. Overview of self-determination theory: An organismic dialectical perspective. 2002 URL: https://psycnet.apa.org/record/2002-01702-001.

61. Hagger M, Chatzisarantis N. Self-determination Theory and the psychology of exercise. International Review of Sport and Exercise Psychology. 2008;1(1):79–103. doi: https://doi.org/10.1080/17509840701827437.

62. Wilson PM, Mack DE, et al. Understanding Motivation for Exercise: A Self-Determination Theory Perspective. Canadian Psychology-Psychologie Canadienne. 2008;49(3):250–256. doi: https://doi.org/10.1037/a0012762.

63. Hagger MS, & Chatzisarantis, N. L. D. . Intrinsic motivation and self-determination in exercise and sport. Intrinsic motivation and self-determination in exercise and sport. Champaign, IL, US: Human Kinetics; 2007 URL: https://psycnet.apa.org/record/2007-05407-000.

64. Murcia JAM, Roman MLD, et al. Peers' influence on exercise enjoyment: A self-determination theory approach. Journal of Sports Science and Medicine. 2008.

65. Teixeira PJ, Carraca EV, et al. Exercise, physical activity, and self-determination theory: a systematic review. Int J Behav Nutr Phys Act. 2012;9(1):78. doi: https://doi.org/10.1186/1479-5868-9-78.

66. Herold F, Zou L, et al. Beyond FITT: addressing density in understanding the dose–response relationships of physical activity with health—an example based on brain health. European Journal of Applied Physiology. 2025. doi: https://doi.org/10.1007/s00421-025-05858-3.

67. Wilson C. Chapter 2 - Brainwriting. Brainstorming and Beyond. Boston: Morgan Kaufmann; 2013 URL: https://www.sciencedirect.com/science/article/pii/B9780124071575000026.

68. Gray D, Brown S, et al. Gamestorming: A playbook for innovators, rulebreakers, and changemakers. " O'Reilly Media, Inc."; 2010 URL: https://gamestorming.com/.

69. Chang W-L, Shao Y-C. Co-creating User Journey Map – A Systematic Approach to Exploring Users' Day-to-Day Experience in Participatory Design Workshops. In: Kurosu M, Hashizume A, editors. Human-Computer Interaction. Cham: Springer Nature Switzerland; 2023. p. 3–17.doi: https://doi.org/10.1007/978-3-031-35596-7_1

70. Segura E, Vidal L, et al. Bodystorming for movement-based interaction design. Human Technology. 2016;12(2):193–251. doi: https://doi.org/10.17011/ht/urn.201611174655.

71. Schleicher D, Jones P, et al. Bodystorming as embodied designing. Interactions. 2010;17(6):47–51. doi: https://doi.org/10.1145/1865245.1865256.

72. Mahatody T, Sagar M, et al. State of the Art on the Cognitive Walkthrough Method, Its Variants and Evolutions. International Journal of Human–Computer Interaction. 2010;26(8):741–785. doi: https://doi.org/10.1080/10447311003781409.

73. Mayring P. Qualitative content analysis: A step-by-step guide. 2021 URL: https://uk.sagepub.com/en-gb/eur/qualitative-content-analysis/book269922.

74. Krippendorff K. Content Analysis: An Introduction to Its Methodology. Fourth Edition ed. Thousand Oaks, California: 2019 https://methods.sagepub.com/book/content-analysis-4e.

75. Mayring P. Qualitative content analysis: theoretical foundation, basic procedures and software solution. 2014. doi: https://doi.org/10.1007/978-94-017-9181-6_13.

76. Schrepp M, Thomaschewski J. Design and validation of a framework for the creation of user experience questionnaires. IJIMAI. 2019;5(7):88–95. doi: https://doi.org/10.9781/ijimai.2019.06.006.

77. Johnson RM. User Experience Research and Usability of Health Information Technology. Technical Communication Quarterly. 2025:1–4. doi: https://doi.org/10.1080/10572252.2025.2455553.

78. Winter D, Hinderks A, et al. Welche UX Faktoren sind für mein Produkt wichtig? Mensch und Computer 2017-Usability Professionals: Gesellschaft für Informatik eV; 2017.doi: https://doi.org/10.18420/muc2017-up-0002

79. Winter D, Schrepp M, et al. Faktoren der User Experience-Systematische Übersicht über produktrelevante UX-Qualitätsaspekte. Mensch und Computer 2015–Usability Professionals: De Gruyter Oldenbourg; 2015. p. 33–41.doi: https://doi.org/10.1515/9783110443882-005

80. Perez FMP, Bellei EA, et al. Decoding user experience in exergames: A systematic scoping review of assessment methods. MethodsX. 2025;14:103054. doi: https://doi.org/10.1016/j.mex.2024.103054.

81. Gao MYZ, Kortum P, et al. Multi-Language Toolkit for the System Usability Scale. International Journal of Human-Computer Interaction. 2020;36(20):1883–1901. doi: https://doi.org/10.1080/10447318.2020.1801173.

82. Laugwitz B, Held T, et al. Construction and Evaluation of a User Experience Questionnaire. Berlin, Heidelberg: Springer Berlin Heidelberg; 2008. p. 63–76.doi: https://doi.org/10.1007/978-3-540-89350-9_6

83. Perrig SAC, von Felten N, et al. Development and Validation of a Positive-Item Version of the Visual Aesthetics of Websites Inventory: The VisAWI-Pos. International Journal of Human–Computer Interaction. 2024;40(20):6622–6646. doi: https://doi.org/10.1080/10447318.2023.2258634.

84. Moshagen M, Thielsch MT. Facets of visual aesthetics. International Journal of Human-Computer Studies. 2010;68(10):689–709. doi: https://doi.org/10.1016/j.ijhcs.2010.05.006.

85. Gediga G, Hamborg K-C, et al. The IsoMetrics usability inventory: An operationalization of ISO 9241-10 supporting summative and formative evaluation of software systems. Behaviour & Information Technology. 1999;18(3):151–164. doi: https://doi.org/10.1080/014492999119057.

86. Fitzgerald A, Huang S, et al. The Exergame Enjoyment Questionnaire (EEQ): An Instrument for Measuring Exergame Enjoyment. 2020 URL: https://scholarspace.manoa.hawaii.edu/bitstream/10125/64158/1/0336.pdf.

87. Brockmyer JH, Fox CM, et al. The development of the Game Engagement Questionnaire: A measure of engagement in video game-playing. Journal of Experimental Social Psychology. 2009;45(4):624–634. doi: https://doi.org/10.1016/j.jesp.2009.02.016.

88. Jennett C, Cox AL, et al. Measuring and defining the experience of immersion in games. International Journal of Human-Computer Studies. 2008;66(9):641–661. doi: https://doi.org/10.1016/j.ijhcs.2008.04.004.

89. Guggenberger B, Jocham AJ, et al. Instrumental Validity of the Motion Detection Accuracy of a Smartphone-Based Training Game. International Journal of Environmental Research and Public Health. 2021;18(16). doi: https://doi.org/10.3390/ijerph18168410.

90. Kaiser W, de Bruin ED, et al. Domain-Specific Evaluation of Exergame Metrics Among Older Adults With Mild Neurocognitive Disorder: Secondary Analysis of 2 Randomized Controlled Trials. JMIR Serious Games. 2025;13:e65878. doi: https://doi.org/10.2196/65878.

91. Guimarães V, Sousa I, et al. Using shoe-mounted inertial sensors and stepping exergames to assess the motor-cognitive status of older adults: A correlational study. DIGITAL HEALTH. 2023;9:20552076231167001. doi: https://doi.org/10.1177/20552076231167001.

92. Konstantinidis EI, Bamidis PD, et al. Physical Training In-Game Metrics for Cognitive Assessment: Evidence from Extended Trials with the Fitforall Exergaming Platform. Sensors. 2021;21(17). doi: https://doi.org/10.3390/s21175756.

93. Petsani D, Konstantinidis E, et al. Digital Biomarkers for Well-being Through Exergame Interactions: Exploratory Study. JMIR Serious Games. 2022;10(3):e34768. doi: https://doi.org/10.2196/34768.

94. Litz E, Ball C, et al. Validation of a Motor-Cognitive Assessment for a Stepping Exergame in Older Adults: Use of Game-Specific, Internal Data Stream. Games Health J. 2020;9(2):95–107. doi: https://doi.org/10.1089/g4h.2019.0081.

95. Wiloth S, Lemke N, et al. Validation of a Computerized, Game-based Assessment Strategy to Measure Training Effects on Motor-Cognitive Functions in People With Dementia. JMIR Serious Games. 2016;4(2):e12. doi: https://doi.org/10.2196/games.5696.

96. Oluwatoyosi BAO, Rachel SR, et al. Dissemination and implementation research in sports and exercise medicine and sports physical therapy: translating evidence to practice and policy. BMJ Open Sport & Exercise Medicine. 2020;6(1):e000974. doi: https://doi.org/10.1136/bmjsem-2020-000974.

97. Landes SJ, McBain SA, et al. An introduction to effectiveness-implementation hybrid designs. Psychiatry Res. 2019;280:112513. doi: https://doi.org/10.1016/j.psychres.2019.112513.

98. Curran GM, Bauer M, et al. Effectiveness-implementation Hybrid Designs: Combining Elements of Clinical Effectiveness and Implementation Research to Enhance Public Health Impact. Medical Care. 2012;50(3). doi: https://doi.org/10.1097/MLR.0b013e3182408812.

99. Curran GM, Landes SJ, et al. Reflections on 10 years of effectiveness-implementation hybrid studies. Frontiers in Health Services. 2022;Volume 2 - 2022. doi: https://doi.org/10.3389/frhs.2022.1053496.

100. Nahum-Shani I, Dziak JJ, et al. MCMTC: A Pragmatic Framework for Selecting an Experimental Design to Inform the Development of Digital Interventions. Frontiers in Digital Health. 2022;Volume 4 - 2022. doi: https://doi.org/10.3389/fdgth.2022.798025.

101. Crossnohere NL, Schuster ALR, et al. Patient-reported outcome measures add value as clinical trial endpoints. Nature Medicine. 2025. doi: https://doi.org/10.1038/s41591-025-03906-1.

102. Dorsey ER, Elbaz A, et al. Global, regional, and national burden of Parkinson's disease, 1990–2016: a systematic analysis for the Global Burden of Disease Study 2016. The Lancet Neurology. 2018;17(11):939–953. doi: https://doi.org/10.1016/S1474-4422(18)30295-3.

103. Tysnes O-B, Storstein A. Epidemiology of Parkinson’s disease. Journal of Neural Transmission. 2017;124(8):901–905. doi: https://doi.org/10.1007/s00702-017-1686-y.

104. Postuma RB, Berg D, et al. MDS clinical diagnostic criteria for Parkinson's disease. Movement disorders. 2015;30(12):1591–1601. doi: https://doi.org/10.1002/mds.26424.

105. Baiano C, Barone P, et al. Prevalence and Clinical Aspects of Mild Cognitive Impairment in Parkinson's Disease: A Meta-Analysis. Movement disorders. 2020;35(1):45–54. doi: https://doi.org/10.1002/mds.27902.

106. Severiano e Sousa C, Alarcão J, et al. Frequency of dementia in Parkinson's disease: A systematic review and meta-analysis. Journal of the Neurological Sciences. 2022;432:120077. doi: https://doi.org/10.1016/j.jns.2021.120077.

107. Gibson LL, Weintraub D, et al. Risk of Dementia in Parkinson's Disease: A Systematic Review and Meta-Analysis. Movement disorders. 2024;n/a(n/a). doi: https://doi.org/10.1002/mds.29918.

108. Aarsland D, Kurz MW. The Epidemiology of Dementia Associated with Parkinson's Disease. Brain Pathology. 2010;20(3):633–639. doi: https://doi.org/10.1111/j.1750-3639.2009.00369.x.

109. Cong S, Xiang C, et al. Prevalence and clinical aspects of depression in Parkinson’s disease: A systematic review and meta-analysis of 129 studies. Neuroscience & Biobehavioral Reviews. 2022;141:104749. doi: https://doi.org/10.1016/j.neubiorev.2022.104749.

110. Broen MPG, Narayen NE, et al. Prevalence of anxiety in Parkinson's disease: A systematic review and meta-analysis. Movement disorders. 2016;31(8):1125–1133. doi: https://doi.org/10.1002/mds.26643.

111. Langeskov-Christensen M, Franzén E, et al. Exercise as medicine in Parkinson’s disease. Journal of Neurology, Neurosurgery & Psychiatry. 2024:jnnp–2023–332974. doi: https://doi.org/10.1136/jnnp-2023-332974.

112. Herold F, Theobald P, et al. The Best of Two Worlds to Promote Healthy Cognitive Aging: Definition and Classification Approach of Hybrid Physical Training Interventions. JMIR Aging. 2024;7:e56433. doi: https://doi.org/10.2196/56433.

113. Gallou-Guyot M, Nuic D, et al. Effectiveness of home-based rehabilitation using active video games on quality of life, cognitive and motor functions in people with Parkinson’s disease: a systematic review. Disabil Rehabil. 2022;44(26):8222–8233. doi: https://doi.org/10.1080/09638288.2021.2022780.

114. Hernandez-Martinez J, Ramos-Espinoza F, et al. Effects of active exergames on physical performance in older people: an overview of systematic reviews and meta-analysis. Frontiers in Public Health. 2024;12. doi: https://doi.org/10.3389/fpubh.2024.1250299.

115. Zhang J, Luximon Y, et al. Effectiveness of exergaming-based interventions for mobility and balance performance in older adults with Parkinson’s disease: systematic review and meta-analysis of randomised controlled trials. Age and Ageing. 2022;51(8):afac175. doi: https://doi.org/10.1093/ageing/afac175.

116. Wang D, Cui WJ, et al. Effectiveness of different exercises in improving postural balance among Parkinson's disease patients: a systematic review and network meta-analysis. Frontiers in Aging Neuroscience. 2023;15. doi: https://doi.org/10.3389/fnagi.2023.1215495.

117. Radder DLM, Lígia Silva de Lima A, et al. Physiotherapy in Parkinson’s Disease: A Meta-Analysis of Present Treatment Modalities. Neurorehabil Neural Repair. 2020;34(10):871–880. doi: https://doi.org/10.1177/1545968320952799.

118. Nilsson M, Franzén E, et al. Svenska riktlinjer för fysioterapi vid Parkinsons sjukdom. 2018.

119. Ni M, Hazzard JB, et al. Exercise Guidelines for Gait Function in Parkinson’s Disease: A Systematic Review and Meta-analysis. Neurorehabil Neural Repair. 2018;32(10):872–886. doi: https://doi.org/10.1177/1545968318801558.

120. Schoneburg B, Mancini M, et al. Framework for understanding balance dysfunction in Parkinson's disease. Movement disorders. 2013;28(11):1474–1482. doi: https://doi.org/10.1002/mds.25613.

121. Denzin NK, Lincoln YS. The SAGE handbook of qualitative research. Fifth edition ed. SAGE handbook of qualitative research 5. Los Angeles, SAGE; 2018 URL: https://us.sagepub.com/en-us/nam/the-sage-handbook-of-qualitative-research/book242504.

122. Tong A, Sainsbury P, et al. Consolidated criteria for reporting qualitative research (COREQ): a 32-item checklist for interviews and focus groups. Int J Qual Health Care. 2007;19(6):349–357. doi: https://doi.org/10.1093/intqhc/mzm042.

123. Dicicco-Bloom B, Crabtree BF. The qualitative research interview. Med Educ. 2006;40(4):314–321. doi: https://doi.org/10.1111/j.1365-2929.2006.02418.x.

124. Hennink M, Kaiser BN. Sample sizes for saturation in qualitative research: A systematic review of empirical tests. Social Science & Medicine. 2022;292:114523. doi: https://doi.org/https://doi.org/10.1016/j.socscimed.2021.114523.

125. Wallin A, Franzén E, et al. Balance Exercise Interventions in Parkinson's Disease: A Systematic Mapping Review of Components, Progression, and Intensity. Parkinsonism & Related Disorders. 2025:107310. doi: https://doi.org/10.1016/j.parkreldis.2025.107310.

126. Hardeman LES, Geerse DJ, et al. Remotely prescribed and monitored home-based gait-and-balance therapeutic exergaming using augmented reality (AR) glasses: protocol for a clinical feasibility study in people with Parkinson's disease. Pilot Feasibility Stud. 2024;10(1):54. doi: https://doi.org/10.1186/s40814-024-01480-w.

127. Hardeman LES, Geerse DJ, et al. Remotely prescribed, monitored, and tailored home-based gait-and-balance exergaming using augmented reality glasses: a clinical feasibility study in people with Parkinson's disease. Frontiers in Neurology. 2024;15:1373740. doi: https://doi.org/10.3389/fneur.2024.1373740.

128. Hoogendoorn EM, Geerse DJ, et al. Cueing-assisted gamified augmented-reality home rehabilitation for gait and balance in people with Parkinson's disease: feasibility and potential effectiveness in the clinical pathway. Zenodo2025.doi: https://doi.org/10.5281/zenodo.15655001

129. Lindop F, Skelly R. Parkinson's Disease: A Multidisciplinary Guide to Management. Elsevier; 2022 URL: https://shop.elsevier.com/books/parkinson-s-disease-a-multidisciplinary-guide-to-management/lindop/978-0-7020-8261-0.

130. Hardeman LES, van Benten E, et al. Do people with Parkinson's disease find a home-based augmented-reality gait-and-balance exercise program acceptable?: a qualitative approach. JMIR Preprints2025.doi: https://doi.org/10.2196/preprints.70802

131. Rieker JA, Reales JM, et al. The Effects of Combined Cognitive-Physical Interventions on Cognitive Functioning in Healthy Older Adults: A Systematic Review and Multilevel Meta-Analysis. Frontiers in Human Neuroscience. 2022;16. doi: https://doi.org/10.3389/fnhum.2022.838968.

132. Torre MM, Temprado J-J. A Review of Combined Training Studies in Older Adults According to a New Categorization of Conventional Interventions. Frontiers in Aging Neuroscience. 2022;13. doi: https://doi.org/10.3389/fnagi.2021.808539.

133. Zhao X, Huang X, et al. The relative effectiveness of different combination modes for exercise and cognitive training on cognitive function in people with mild cognitive impairment or Alzheimer's disease: a network meta-analysis. Aging & Mental Health. 2022:1–11. doi: https://doi.org/10.1080/13607863.2022.2026879.

134. Meng Q, Yin H, et al. The effect of combined cognitive intervention and physical exercise on cognitive function in older adults with mild cognitive impairment: a meta-analysis of randomized controlled trials. Aging Clinical and Experimental Research. 2022;34(2):261–276. doi: https://doi.org/10.1007/s40520-021-01877-0.

135. Gómez-Soria I, Marin-Puyalto J, et al. Effects of multi-component non-pharmacological interventions on cognition in participants with mild cognitive impairment: A systematic review and meta-analysis. Arch Gerontol Geriatr. 2022;103:104751. doi: https://doi.org/10.1016/j.archger.2022.104751.

136. Yang C, Moore A, et al. Effectiveness of Combined Cognitive and Physical Interventions to Enhance Functioning in Older Adults With Mild Cognitive Impairment: A Systematic Review of Randomized Controlled Trials. Gerontologist. 2020;60(8):633–642. doi: https://doi.org/10.1093/geront/gnz149.

137. Gallou-Guyot M, Mandigout S, et al. Cognitive and physical impact of cognitive-motor dual-task training in cognitively impaired older adults: An overview. Neurophysiol Clin. 2020;50(6):441–453. doi: https://doi.org/10.1016/j.neucli.2020.10.010.

138. Biazus-Sehn LF, Schuch FB, et al. Effects of physical exercise on cognitive function of older adults with mild cognitive impairment: A systematic review and meta-analysis. Arch Gerontol Geriatr. 2020;89:104048. doi: https://doi.org/10.1016/j.archger.2020.104048.

139. Weng W-H, Yeh N-C, et al. Effects of motor-cognitive training on cognitive function and gait performance in older adults with dementia: a systematic review and meta-analysis. Scientific Reports. 2025;15(1):24915. doi: https://doi.org/10.1038/s41598-025-09582-y.

140. Bautmans I, Knoop V, et al. WHO working definition of vitality capacity for healthy longevity monitoring. Lancet Healthy Longev. 2022;3(11):e789–e796. doi: https://doi.org/10.1016/s2666-7568(22)00200-8.

141. Hara M, Murakawa Y, et al. Feasibility of Somato-Cognitive Coordination Therapy Using Virtual Reality for Patients with Advanced Severe Parkinson's Disease. J Parkinsons Dis. 2024;14(4):895–898. doi: https://doi.org/10.3233/jpd-240011.

142. Binkofski F, Fink GR, et al. Introduction: higher motor cognition - from basic neuroscience to apraxia. Neuroimage. 2007;36 Suppl 2:T1. doi: https://doi.org/10.1016/j.neuroimage.2007.03.013.

143. Eaves DL, Riach M, et al. Motor Imagery during Action Observation: A Brief Review of Evidence, Theory and Future Research Opportunities. Frontiers in Neuroscience. 2016;10. doi: https://doi.org/10.3389/fnins.2016.00514.
